# Exploiting Orthogonal C–C Cross-Coupling Reactions for Chemistry-on-the-Complex: Modular Assembly of 2,6-Di(quinolin-8-yl)pyridine Ruthenium(II) Photosensitizer Triads

**DOI:** 10.1021/acs.inorgchem.3c03380

**Published:** 2024-02-19

**Authors:** Alexander Kleine, Ulrich S. Schubert, Michael Jäger

**Affiliations:** † Laboratory of Organic and Macromolecular Chemistry (IOMC), Friedrich Schiller University Jena, Humboldstr. 10, 07743 Jena, Germany; ‡ Center for Energy and Environmental Chemistry Jena (CEEC Jena), Friedrich Schiller University Jena, Philosophenweg 7a, 07743 Jena, Germany

## Abstract

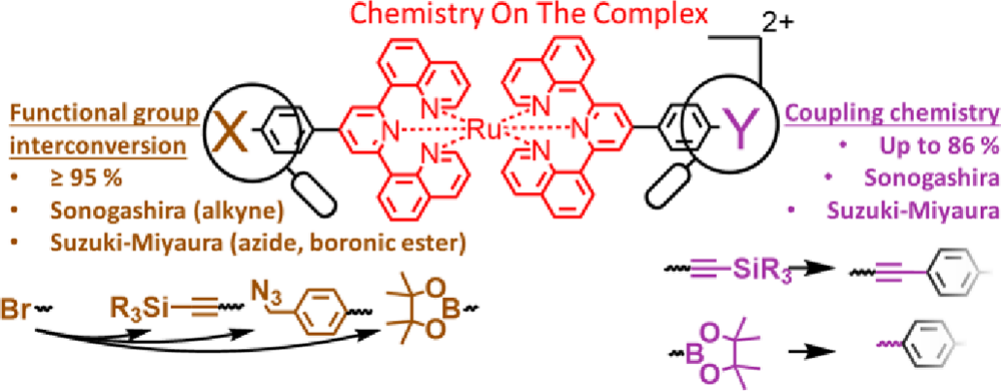

In this work, we present a concise modular assembly strategy using one universal heteroleptic 2,6-di(quinolin-8-yl)pyridine-based ruthenium(II) complex as a starting building block. Extending the concept from established ligand modifications and subsequent complexation (*classical route*), the later appearing chemistry-on-the-complex methodology was used for late-stage syntheses, *i.e.*, assembling discrete building blocks to molecular architectures (here: dyad and triads). We focused on Suzuki–Miyaura and Sonogashira cross-couplings as two of the best-known C–C bond forming reactions. Both were performed on one building block complex bearing a bromine and TIPS-protected alkyne for functional group interconversion (bromine to TMS-protected alkyne, a benzyl azide, or a boronic acid pinacol ester moiety with ≥95% isolated yield and simple purification) as well as building block assemblies using both a triarylamine-based donor and a naphthalene diimide-based acceptor in up to 86% isolated yield. Additionally, the developed purification *via* automated flash chromatography is simple compared to tedious manual chromatography for ruthenium(II)-based substrates in the *classical route*. Based on the preliminary characterization by steady-state spectroscopy, the observed emission quenching in the triad (55%) serves as an entry to rationally optimize the modular units *via* chemistry-on-the-complex to elucidate energy and electron transfer.

## Abstract 2

The present work shows the investigation of photosensitizer triad syntheses *via*chemistry-on-the-complex. The heteroleptic bis(di(quinoline-8-yl)pyridine) ruthenium(II)-based parental photosensitizer was transformed in terms of functional group interconversion from bromide (to azide, protected alkyne, boronic acid ester), and coupling chemistry to introduce both electron-rich and -deficient moieties with simple workup procedures after chemistry-on-the-complex throughout.

## Introduction

Ruthenium(II) complexes based on 2,6-di(quinolin-8-yl)pyridine (dqp) combine advantageous geometrical features of 2,2′:6′,2″-terpyridine-based complexes (tpy-based) with beneficial photophysical properties as found for 2,2′-bipyridine-based complexes (bpy-based). Hence [Ru(dqp)_2_]^2+^-based complexes are ideally suited to mimic vectorial light-driven charge separation processes in nature: In such assemblies, the photosensitizer (P) bears both electron-acceptor (A) and -donor (D) units to form linear D-P-A architectures.^1^ The high efficacy of solar-to-chemical-energy conversion was demonstrated for small-molecule arrays,^2^ and recently, also for polymers^3,4^–leading to long-lived charge-separated states.^4^

**Scheme 1:**
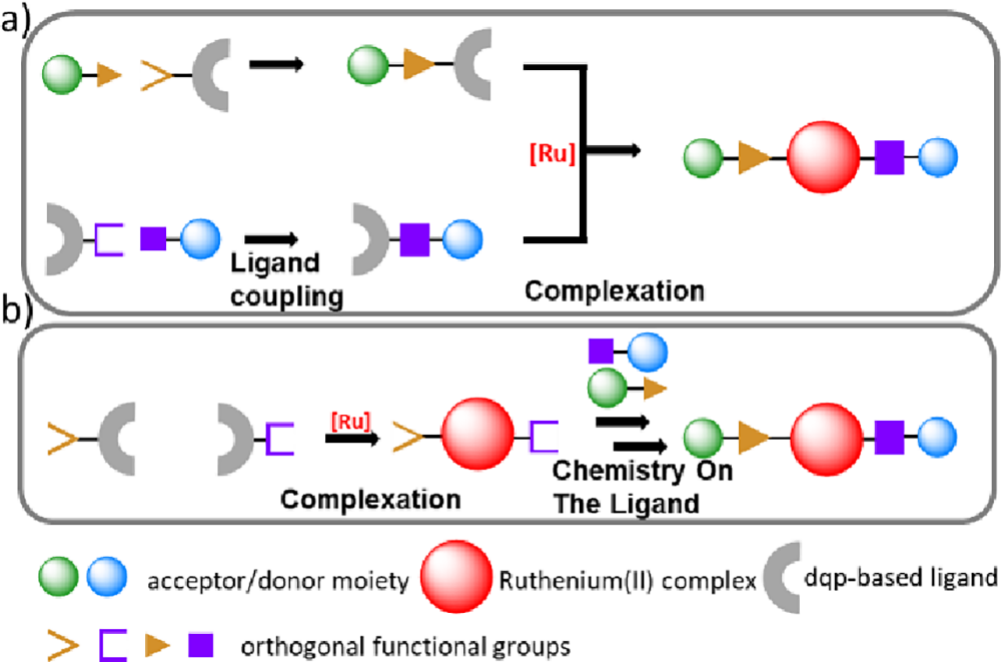
Schematic Representation of D-P-A Triad Syntheses *via* (a) the Classical Route or (b) the Chemistry-on-the-Complex Route

Fueled by the rapid progress to unravel the functional requirements, the synthetic methodologies to provide access to sophisticated systematic assemblies impose, to date, a severe practical challenge. For the introduction of donor- and/or acceptor-moieties, generally the *classical route* is pursued (Scheme 1a), *i.e.*, the ligands’ functional groups (shown in orange/purple) are used to attach the desired redox-active units (shown in blue/green) prior to final stepwise complexation.^2,5^ Although the early steps benefit from high yields and established purification procedures applying standard organic chemistry protocols, the subsequent stepwise complexation imposes harsh reaction conditions. This leads to implications with respect to the introduction of sensitive functional moieties and the corresponding side reactions thereof. In addition, customized purification protocols are often required based on manual column chromatography and ion-exchange steps, which, in turn, hamper the progress of the field in practical terms. Specific to the family [Ru(dqp)_2_]^2+^, the formation of coordination isomers (*meridionalvs* detrimental *cis-* or *trans-facial*) is most challenging, as the aqueous conducting salt-containing manual column chromatography is often unselective toward the same charge and size of larger structures, while crystallization protocols become uncompetitive. At this stage, a preestablished ancestral Ru(II) complex with simple purification appears optimal (*vide supra*).

**Scheme 2:**
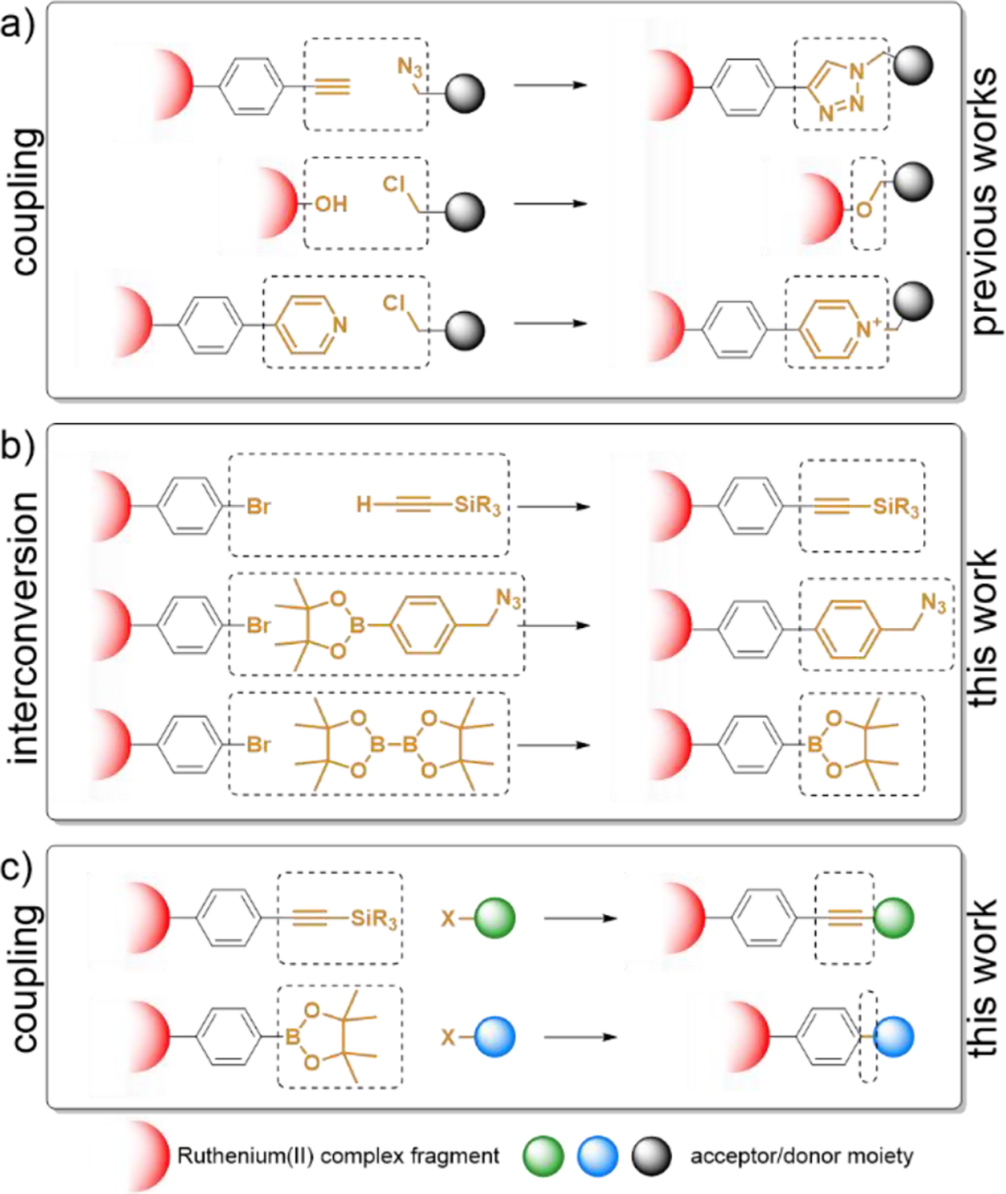
Schematic Representation of Synthetic Linkage Strategies for [Ru(dqp)_2_]^2+^-Based Complexes Using Chemistry-on-the-Complex (a) Previous coupling strategies. (b) Our strategy for the introduction of various functional groups *via* interconversion and (c) subsequent coupling with cross-coupling chemistry.

Recently, various reports demonstrated the versatility of the chemistry-on-the-complex approach (Scheme 1b), *i.e.*, where ligands with orthogonal functional groups are FIRST introduced *via* stepwise complexation prior to the coupling of the requested redox-active units. The success of such an approach requires efficient diverse functional group chemistries as well as reliable and simple purification methods. Notably, this combination was demonstrated for nonconjugated linkages *via* ester^6^ and ethers^3,4,7−10^ descending from a hydroxy group on the complex, triazoles formed from alkynes on the complex,^3,4,9^ or *N*-substituted pyridinium linkers from pyridine-units on the complex (Scheme 2a).^9^ In light of previously reported coupling reactions that were already successfully demonstrated for [Ru(tpy)_2_]^2+^ or [Ru(bpy)_3_]^2+^ complexes,^11^ the chemistry-on-the-complex approach can unambiguously circumvent synthetic limitations, given that the diversification of compounds becomes easier and the purification after complexation is simplified and generalized. For example, the complexation step requires the solubility of both the highly polar Ru-source (typically a +2 or +3 solvento-complex) and the organo-soluble ligand. In the case of the building block approach, optimized conditions were applied to maximize the yield of the universal Ru building block and to remove coordination byproducts. In contrast, the conventional route to assemble the D–P–A triad in the final step requires the coordination of the larger ligand fragment as well as the potentially sensitive donor or acceptor sites. In addition, the inevitable coordination byproducts must be removed in that case. On the other hand, if the final coordination step is devoid of such byproducts, it offers the possibility to save precious Ru-complexes. In addition, the usage of a bifunctional Ruthenium building block *via* chemistry-on-the-complex benefits from a sufficient organo-solubility that permits to directly apply conventional C–C coupling conditions (*vide infra*). In essence, the conventional route benefits from simpler syntheses of the full ligand framework and may save rare Ru but faces challenges to ensure an efficient final coordination step (*vide supra*). The chemistry-on-the-complex will be shown to benefit from one universal precursor for diversification and the ability to directly apply typical C–C coupling conditions without the need of chromatographic purification which is very challenging to separate byproducts that originate from side reactions during complexation and differ only in the functional group pattern (*vide infra*). In view of the fundamental interest to design and assemble D–P–A systems for energy- and electron-transfer, the presented methodology offers a complementary approach to conventional routes and, thus, is believed to shorten and simplify the synthetic efforts.

In this contribution, we embark to utilize the recent progress on modern (multi)functional ligand syntheses, *i.e.*, based on Suzuki–Miyaura cross-couplings^12^ or optimized Kröhnke- and Skraup-type syntheses.^5^ Despite harsh conditions for complexation, the range of conceivable functional group patterns^5,13^ served as an entry toward one orthogonal universal *bis*-functionalized [Ru(dqp)_2_]^2+^-based parental complex for chemistry-on-the-complex. Notably, these facile chemical transformations after the complexation step avoid reoptimization and severe purification challenges with respect to the conventional approach (transformation before complexation). We will show the introduction, interconversion, and utilization of the orthogonal functional groups *via* Suzuki–Miyaura and/or Sonogashira cross-coupling reactions (Scheme 2b,c). These transformations represent two of the most versatile methodologies that render this method amenable to related fields such as charge accumulation,^14^ artificial photosynthesis,^15^ singlet O_2_ generation,^16,17^ hydrogen generation,^18^ photodynamic therapy,^19,20^ or cellular imaging.^21^

## Results and Discussion

Introduced in detail above, improved synthetic methods are highly desired to, *e.g.*, easily introduce sensitive donor and acceptor moieties. In the past (Scheme 2a), such reactions required the individual syntheses of two corresponding ligands and the stepwise coordination afterward, which, in turn, limited the accessible set of functionalities due to excessively tedious synthetic efforts. To overcome this constraint, we embarked to investigate the introduction of orthogonal functional groups *via* synthetically simple and robust methods, starting from ONE single parental ligand (Scheme 2b) for the descending photosensitizer (P) building block, which is then diversified by chemistry-on-the-complex.

**Scheme 3:**
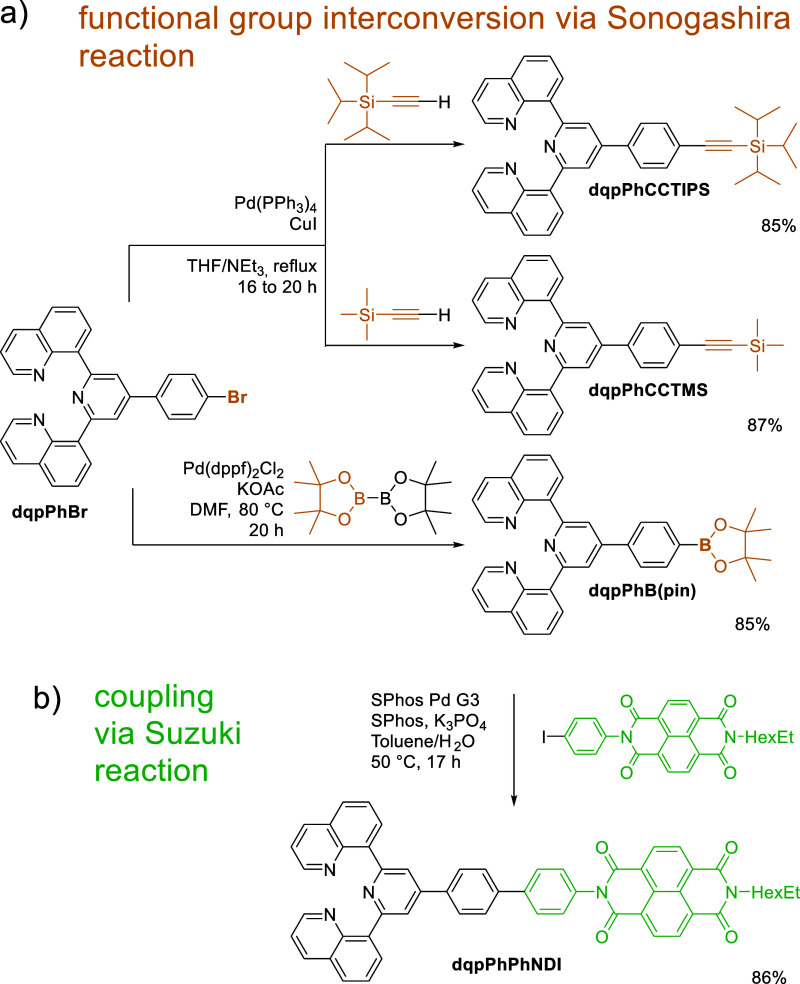
Schematic Representation of the Syntheses of (a) Ligands Bearing a Functional Group Starting from dqpPhBr and (b) Exemplary Conversion of a Functional Group

Specifically, we focus on both Sonogashira and Suzuki–Miyaura cross-coupling chemistries as universal C–C bond forming reactions, as both are widely utilized for organic compounds. As illustrated in Scheme 3, we started from the established 4-bromophenyl-dqp (dqpPhBr). As the bromo group is prone to serve for C–C cross-coupling, it can also be readily interconverted to functional groups, *i.e.*, a boronic acid ester *via* Suzuki–Miyaura reaction, or a protected alkyne *via* Sonogashira reaction. To assess the orthogonality criterion of the latter, we report on the scope of orthogonal protecting groups for alkyne groups. Two of the most-versatile ones are trimethylsilyl (TMS), which is readily deprotected under mild alkaline conditions in protic solvents, and tri(*iso*-propyl)silyl (TIPS), which requires more forcing conditions (fluoride or strong alkaline conditions) for effective deprotection.

In the following, the results of the reaction attempts are evaluated by NMR spectroscopy, ESI-ToF mass spectrometry, size-exclusion chromatography (SEC), and UV–vis spectroscopy. Note, that the used size-exclusion chromatography setup records absorption spectra (270 to 600 nm) throughout the measurement and, thus, enables for spectral differentiation of compounds. The [Ru(dqp)_2_]^2+^-based complexes can be identified through their characteristic MLCT-band between 500 and 600 nm, while organic compounds often express characteristic signals at shorter wavelengths. Hence, modern SEC analysis serves to augment classical adsorption chromatography due to discernment of specimen with comparable polarity but different hydrodynamic volumes. Importantly, the elution volumes help to assess the volume-to-molar-mass ratio of the compounds, in particular to identify limiting side reactions (*vide infra*).

In the following, the heteroleptic [Ru(dqpPh**X**)(dqpPh**Y**)]^2+^ complexes will be abbreviated according to their functional groups (**X**/**Y**) for the sake of simplicity.

### Ligand Syntheses

Implementing our idea of a *bis*-functional [Ru(dqp)_2_]^2+^ complex, we started from 4-bromophenyl-dqp (dqpPhBr). Despite moderate yields, dqpPhBr can be readily synthesized in two steps starting from abundant starting materials.^5^ From this parental ligand, trimethylsilyl- (TMS) and tri(*iso*-propyl)silyl-protected (TIPS) alkyne decorated ligands were prepared, which can serve for Sonogashira cross-couplings or copper-catalyzed azide–alkyne cycloaddition reactions (Scheme 3a, dqpPhCCTIPS and dqpPhCCTMS). In addition, a boronic acid ester derivative for Suzuki–Miyaura cross-couplings was prepared in comparably good yield of around 85% (Scheme 3a, dqpPhB(pin)) including automated flash column chromatography.

For comparison to the chemistry-on-the-complex approach (*vide infra*), a naphthalene diimide electron acceptor unit was introduced in good yields resembling those of the classical route (Scheme 3b). These synthetic protocols including general purification procedures are either literature-known (in case of dqpPhCCTIPS),^3^ or easily transmitted from similar reactions on other organic substrates.

### Stepwise Complexation

**Scheme 4:**
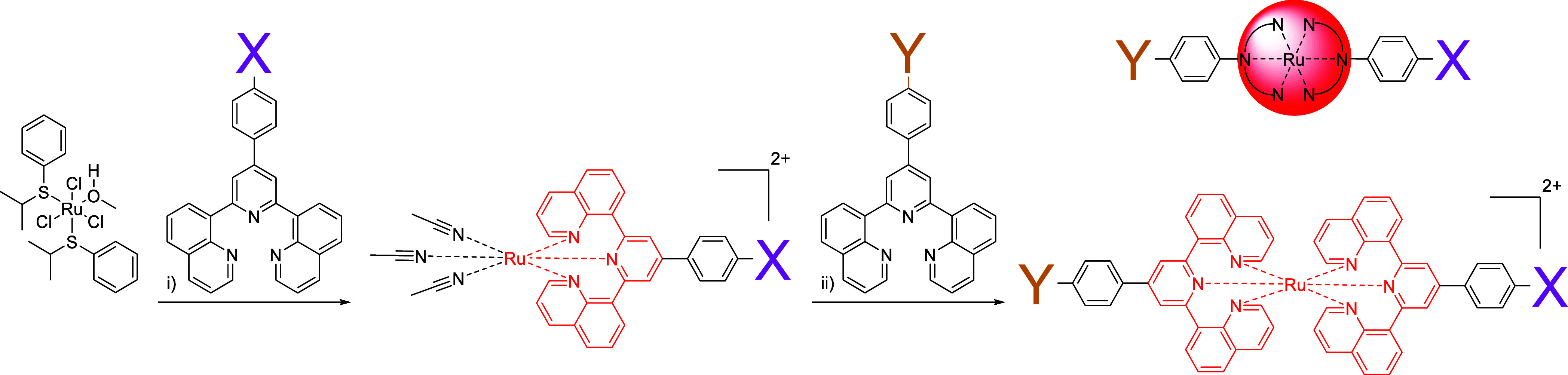
Schematic Representation of the Two-Fold Complexation of Orthogonal Functionalized dqp Ligands on a Ru(II) Core X (purple) and Y (yellow) are functional groups according to Table 1. Note, the simplified schematic representation of the complex used hereafter. (i) 1. ligand, microwave irradiation, CH_3_CN, 130 °C, 2.5 h; 2. H_2_O, EtOH, and AgNO_3_, 80 °C, overnight. (ii) Ligand, DMF, 120 °C, 2 days.

With the synthesized ligands in hand, selected heteroleptic ruthenium complexes were prepared stepwise (Scheme 4 and Table 1). Following the classical route, we introduced one ligand with a functional group (dqpPhBr) and the prefunctionalized acceptor-ligand dqpPhPhNDI (Table 1, entry A; see also Scheme 3), isolating [Ru(dqpPh**Br**)(dqpPh**PhNDI**)]^2+^ in 40% yield but requiring extensive purification. In addition to precipitation in diethyl ether to remove organic residuals, manual column chromatography with salt-containing aqueous eluents (CH_3_CN/H_2_O/KNO_3(aq. sat.)_ 40:4:1) was required to isolate the product after anion metathesis to hexafluorophosphate. This finding is in line with the substantial efforts made in the complexation steps.

**Table 1:** Syntheses of bis-Functionalized [Ru(dqp)_2_]^2+^ Complexes According to Scheme 4

entry	X	Y	product	yield^a^
A	**Br**	**PhNDI**	[Ru(dqpPh**Br**)(dqpPh**PhNDI**)]^2+^	40%
B	**-CC-TIPS**	**-CC-TMS**	[Ru(dqpPh**CCTIPS**)(dqpPh**CCTMS**)]^2+^	not isolatable
C	**Br**	**-CC-TIPS**	[Ru(dqpPh**Br**)(dqpPh**CCTIPS**)]^2+^	36%

a Total yield after both complexation steps. NDI is *N*-(2′-ethylhexyl)-naphthalene-1,4,5,8-tetracarboxyl acid diimide-*N*′**-yl.

Subsequently, we tested the introduction of orthogonally deprotectable alkyne ligands, *i.e.*, dqpPhCCTIPS in the first step and dqpPhCCTMS in the second step (Table 1, entry B). Although the first complexation step yields the targeted complex [Ru(dqpPh**CCTIPS**)(CH_3_CN)_3_]^2+^, the second step with dqpPhCCTMS leads to decomposition of the alkyne group during the harsh reaction conditions (120 °C), while the TIPS-protected alkyne stays intact. Hence, we targeted the stepwise coordination of dqpPhBr and then dqpPhCCTIPS, which yielded the bifunctional complex [Ru(dqpPh**Br**)(dqpPh**CCTIPS**)]^2+^ (Table 1, entry C). In this universal heteroleptic complex for chemistry-on-the-complex, the bromo-terminus serves for C–C coupling reactions, while the alkyne group can be utilized after deprotection. The removal of facial isomers is achieved *via* manual column chromatography on silica with salt-containing aqueous eluents (*e.g.*, CH_3_CN/H_2_O/KNO_3(aq. sat.)_ 40:4:1), followed by anion exchange to hexafluorophosphate. Due to incomplete separation, impure fractions were further purified via successive crystallization steps. In total, a yield of 36% over both steps was obtained that is comparable to that of entry A. More importantly, such extensive purification is performed only once for the complexation steps (*vide infra*).

### Chemistry-on-the-Complex: Functional Group Interconversion

**Scheme 5:**
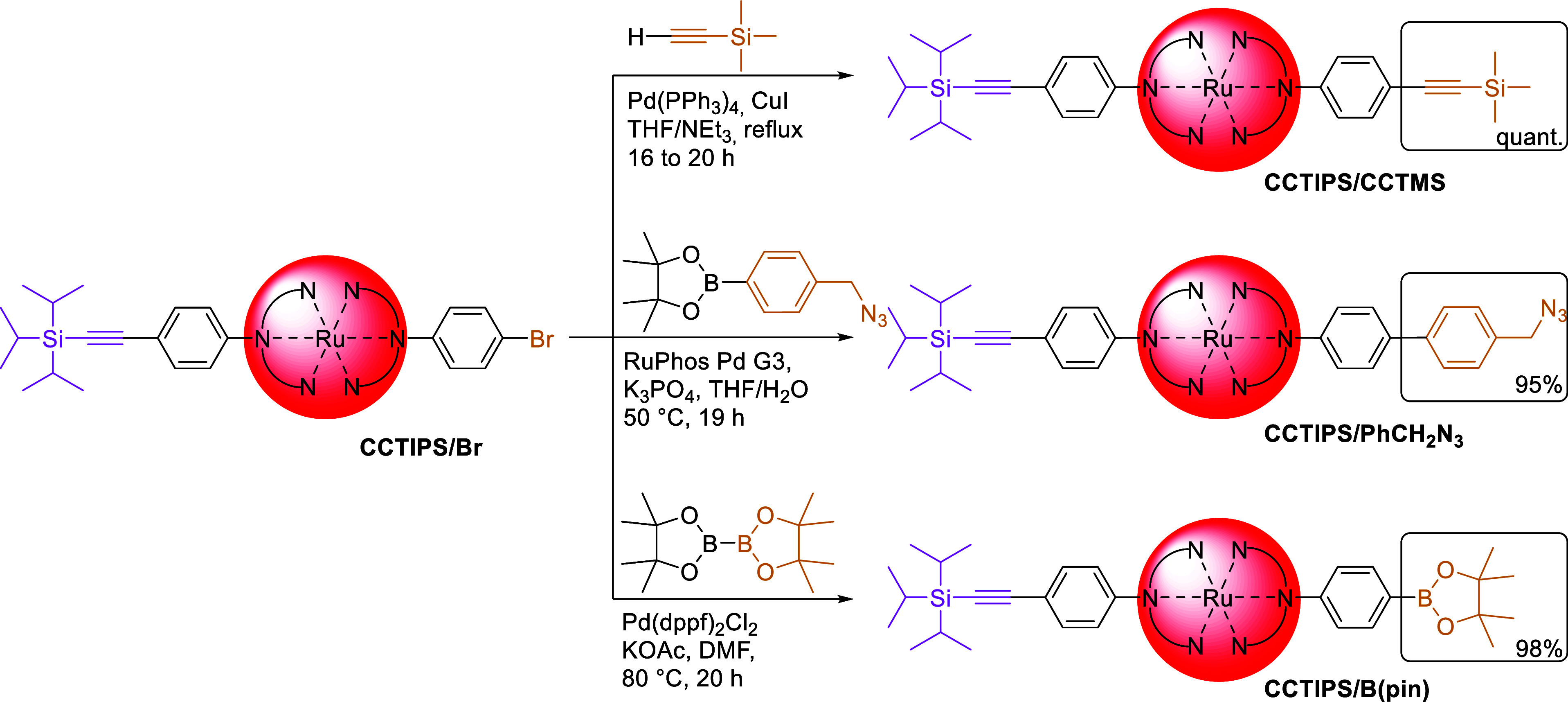
Schematic Representation of the Cross-Coupling Chemistry-on-the-Complex for Functional Group Interconversion Starting from [Ru(dqpPh**CCTIPS**)(dqpPh**Br**)]^2+^ (CCTIPS/Br) for conversion of bromide into protected alkyne *via* Sonogashira cross-coupling (top), or into either benzyl azide (middle) or boronic acid pinacol ester (bottom) *via* Suzuki–Miyaura cross-coupling. Note the analogy to the chemistry on the ligand before complexation (Scheme 3a).

Continuing with the heteroleptic CCTIPS/Br complex, we tested the interconversion of the bromo-group similar to the classical route but using chemistry-on-the-complex (Scheme 5). We introduced a trimethylsilyl-protected alkyne (-CCTMS) *via* Sonogashira cross-coupling to yield the CCTIPS/CCTMS functionalized complex–previously inaccessible *via* complexation–in nearly quantitative yield. In addition, we exploited the Suzuki–Miyaura cross-coupling with both 4-(azidomethyl)phenylboronic acid pinacol ester (-PhCH_2_N_3_), and with bis(pinacolato)diboron under anhydrous conditions (-B(pin)) to introduce either an azide group amenable for copper-catalyzed azide–alkyne cycloaddition (CuAAC) reactions or the boronic ester for further Suzuki–Miyaura cross-coupling. Both functional group interconversions proceeded in excellent yields of 95% and 98%, respectively. Note that both the interconversion into the protected alkyne (CCTMS) and the boronic ester (B(pin)) on the complex proceeded in slightly higher yields compared to the respective cross-couplings performed on the ligand beforehand (≥98% *vs* 85%, Scheme 3a). More importantly, a simplified purification method consisting only of washing with water and precipitation into diethyl ether without the need for column chromatography was applied. The simple and reliable purification procedure is a main advantage of these syntheses compared to the separate stepwise complexation of these ligands, leading to laborious product mixtures in need of excessive purification processes. In addition, sensitive functional groups such as the trimethylsilyl-protected alkyne can be introduced, demonstrating the versatility of the chemistry-on-the-complex approach.

### Chemistry-on-the-Complex: Alkyne Deprotection

**Figure 1:**
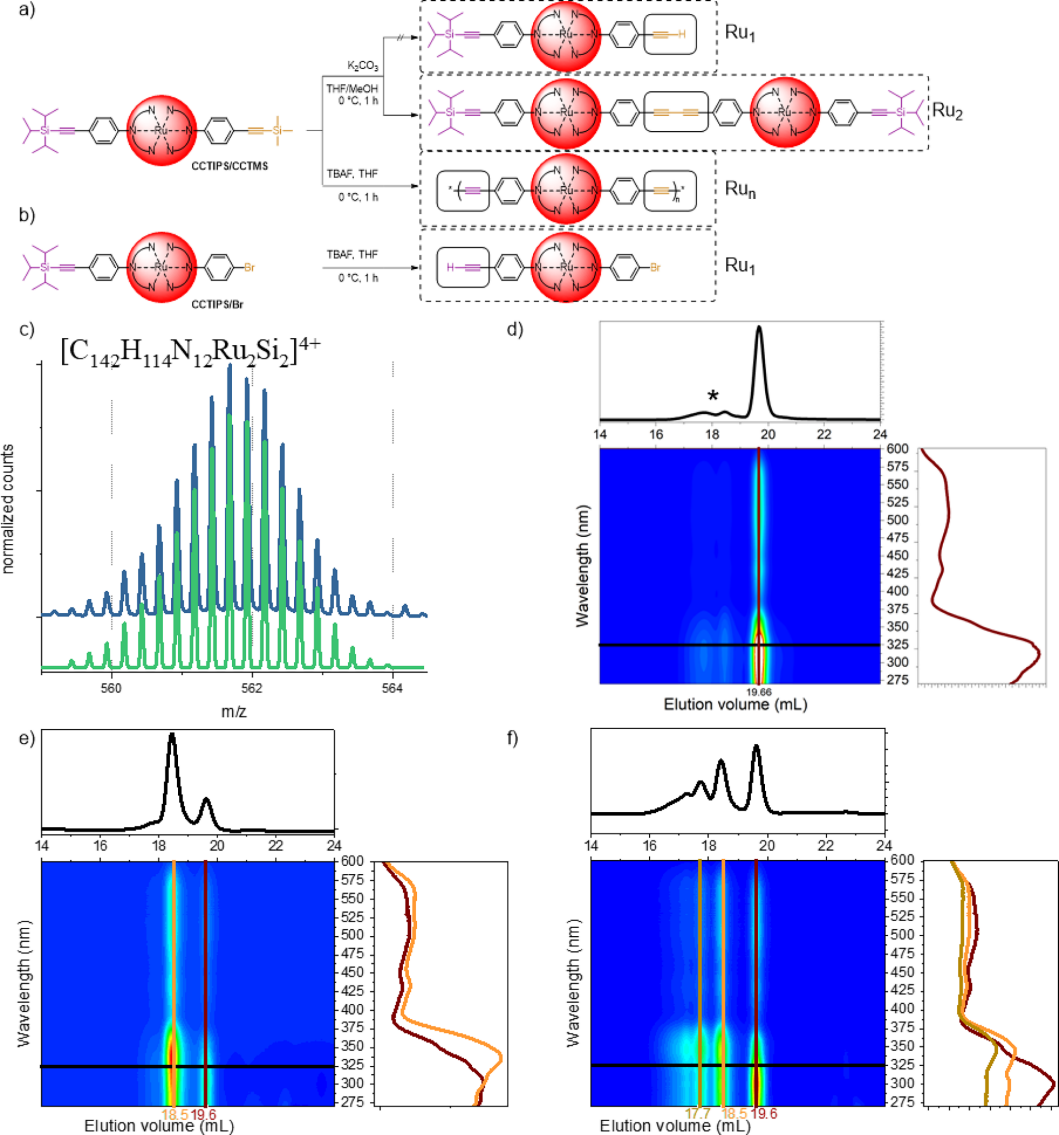
Schematic representation of the deprotection of (a) [Ru(dqpPh**CCTIPS**)(dqpPh**CCTMS**)]^2+^ (CCTIPS/CCTMS) under basic conditions (top, middle) or with fluoride (bottom) and (b) [Ru(dqpPh**CCTIPS**)(dqpPh**Br**)]^2+^ (CCTIPS/Br) with fluoride. Dashed boxes indicate that only crude products were analyzed, instead of isolated compounds. (c) Section of the ESI-ToF mass spectrum after the conversion of the CCTIPS/CCTMS functionalized Ru-complex with potassium carbonate. Green isotope pattern is the calculated pattern for [C_142_H_114_N_12_Ru_2_Si_2_]^4+^ ([Ru(dqpPh**CCTIPS**)(dqpPhCC–CCPhdqp)Ru(dqpPh**CCTIPS**)]^4+^; ***Ru*_2_**). (d) SEC elugram before deprotection of silyl group(s). The asterisk marks dimeric contaminations due to unintended deprotection (see also Figure S35). (e) SEC elugram after deprotection with carbonate (mainly ***Ru*_2_**) or (f) with fluoride (***Ru*_*n*_**). SEC: DMAc + 0.08% NH_4_PF_6_. Traces on top of SEC elugrams are extracted at a 350 nm detection wavelength. Traces right to the elugrams are extracted from UV–vis spectra at selected elution volumes of monomeric (red), dimeric (orange), or oligomeric (brown) complex species.

After the successful synthesis of [Ru(dqpPh**CCTIPS**)(dqpPh**CCTMS**)]^2+^, we tested the deprotection of the masked alkyne under a nitrogen atmosphere (Figure 1a). In the case of deprotection under mild basic conditions, one would expect a one-sided deprotection (only -CCTMS to -CCH, top). Instead, a dimer is formed upon oxidative coupling. The butadiyne linkage (Figure 1a, middle) was identified by isotope simulation of the ESI-ToF mass spectrometry data (Figure 1c). Besides the MS characterization, we applied size-exclusion chromatography (SEC) for reaction monitoring of these syntheses. While the CCTIPS/CCTMS functionalized complex elutes after 19.6 mL (Figure 1d), the deprotection reaction mixture reveals the new main species at 18.5 mL elution volume (Figure 1e) with increased intensity compared to the starting material. The smaller elution volume hints toward an increased molar mass due to the larger hydrodynamic volume of the dimeric compound.

Matching this side reaction, the simultaneous deprotection of both alkynes with tetrabutyl ammonium fluoride forms butadiyne-bridged oligomeric structures (Figure 1a, bottom). Besides the monomeric (19.6 mL, red line) and dimeric species (18.5 mL, orange line), further oligomers in lower amounts (17.7 mL and less, brown line) can be detected. Note that insoluble parts indicate even larger oligomers were obtained. We assign these Glaser-related side reactions in both deprotection reactions to residual copper salts from the prior Sonogashira reaction (Scheme 5). Matching this assignment, the deprotection of [Ru(dqpPh**CCTIPS**)(dqpPh**Br**)]^2+^ with fluoride (Figure 1b) proceeds without such side reaction and the free alkyne can be assigned both *via*^1^H NMR and SEC (Figures S17 and S47 in the Supporting Information; no isolation performed). In this case, the ligand was functionalized *via* the classical route, and residual copper salts were removed upon automated column chromatography at this stage. In view of our goal to establish a simple protocol, we deliberately omitted the possibility to remove copper salts by column chromatography, which will be more tedious and less efficient in the case of aqueous–organic eluents required for Ru(II) complexes (*vide supra*). However, the consequent and excessive removal of copper residuals could be promising in future studies but is beyond the scope of this work. Likewise, the formation of butadiyne linkages may be attractive to form metallo-oligomers and -polymers containing [Ru(dqp)_2_]^2+^ units in the main chain.

### Chemistry-on-the-Complex: Introduction of Acceptor and Donor Moieties

As the bisalkyne complex led to side reactions upon deprotection, we focused on the boronic ester, resulting from the interconversion of the bromo-group into the pinacol ester in high yields (*vide supra*).

The commercial availability of boronic acid derivatives, even growing, is still limited. In contrast, respective halides are often easily available and cheaper. Thus, we decided to use the pinacol boronic ester functionalized complex ([Ru(dqpPh**B(pin)**)(dqpPh**CCTIPS**)]^2+^; *vide supra*) and different halides instead of *vice versa*. Even if the functionalization on the complex requires one more synthetic step, it subsequently enables the functionalization with halides instead of boronic acid derivatives, which, in many cases, must also be synthesized likewise. Thus, the applicability of the pinacol boron substituent on the complex is more versatile compared to the bromo-group when dealing with Suzuki–Miyaura cross-couplings.

**Scheme 6:**
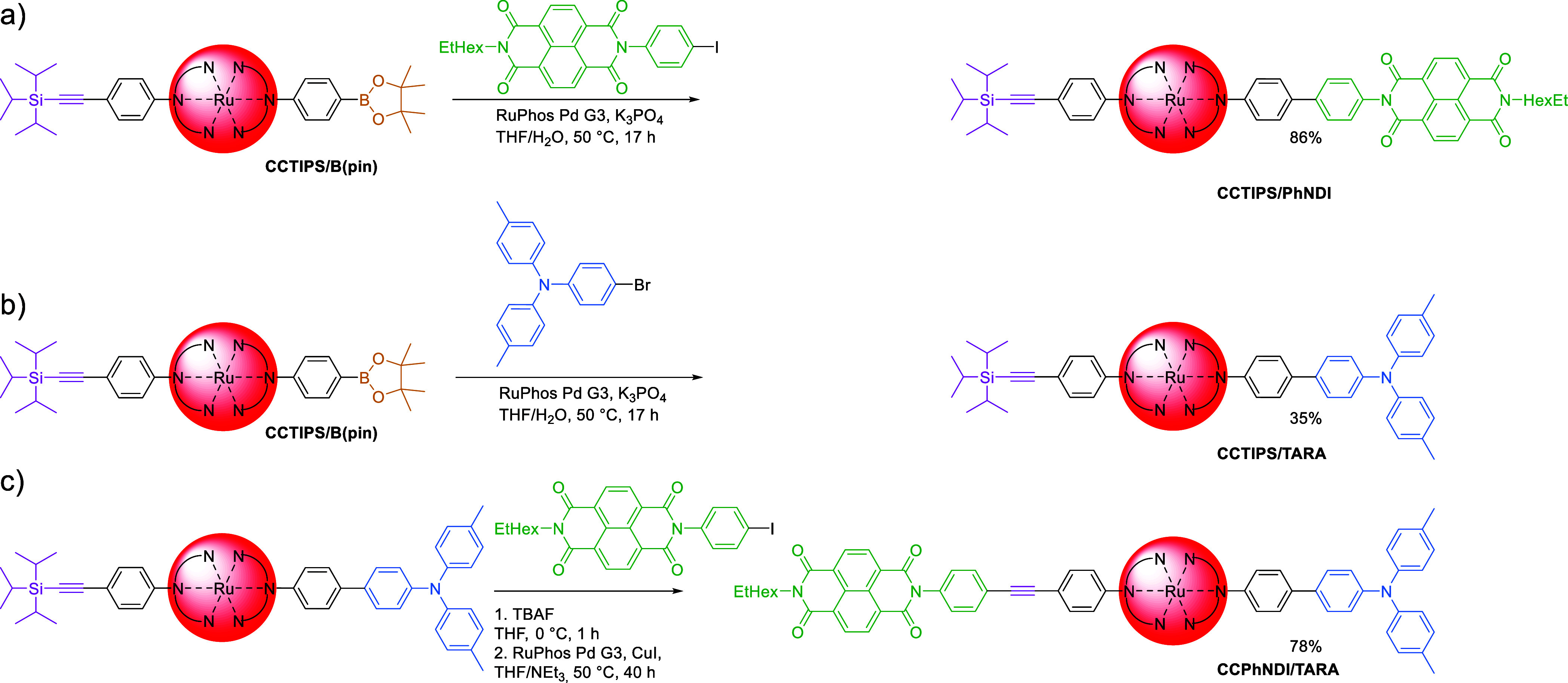
Schematic Representation of the Functionalization of [Ru(dqpPhCCTIPS)(dqpPhB(pin))]^2+^ (CCTIPS/B(pin)) *via* Suzuki–Miyaura Cross-Coupling (a, b) Followed by Sonogashira Cross-Coupling (c)

**Figure 2:**
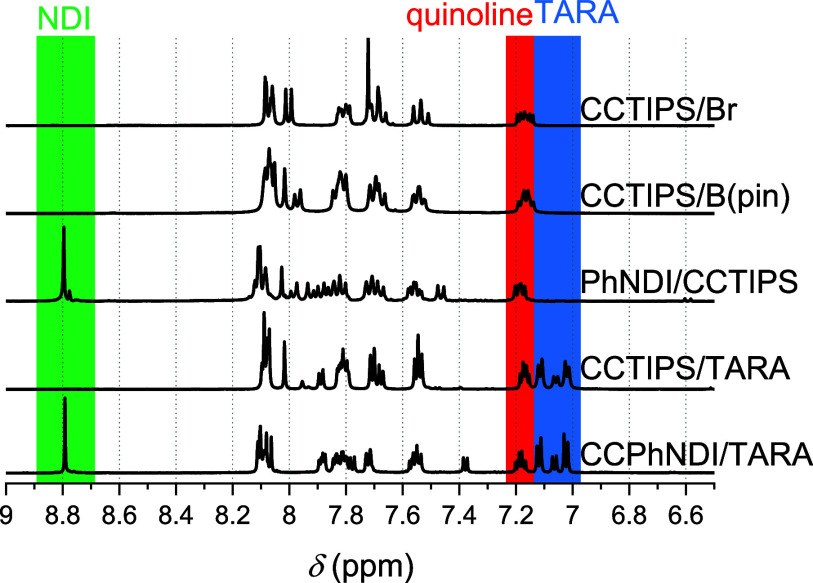
Sections of ^1^H NMR spectra (all in CD_2_Cl_2_) of CCTIPS/Br (300 MHz), CCTIPS/B(pin) (400 MHz), PhNDI/CCTIPS (400 MHz), CCTIPS/TARA (600 MHz), and CCPhNDI/TARA (600 MHz) (top to bottom). Green box highlights signals assigned to the NDI moiety, the red box to the characteristic signal of a quinoline-proton in a meridional complex, and the blue box to the TARA moiety.

To show the applicability of [Ru(dqpPh**B(pin)**)(dqpPh**CCTIPS**)]^2+^, we performed Suzuki–Miyaura coupling with NDIPhI (Scheme 6a). The Suzuki–Miyaura cross-coupling resulted in the desired product in good yield (86%). The development of the converted functional group can be easily followed by ^1^H NMR spectroscopy (Figure 2). After the conversion of the bromo group, a characteristic signal of the pinacol’s methyl groups results (12*H*, see Figure S20) while the TIPS-signals (21*H*, see Figure S11*vs*Figure S20) as well as dqp signals (*e.g.*, 4*H*, red box) remain with matching integrals. Subsequent introduction of the NDI moiety leads to a loss of the pinacol signals, while a characteristic NDI signal around 8.8 ppm (green box) is observed. Still, the respective signals assigned to dqp and TIPS remain observable. In addition, the product mixture required only little purification, *i.e.*, aqueous washing and precipitation in diethyl ether. Likewise, a similar reaction performed *via* the classical route (Scheme 3) results in comparable yields (both 86%) of the desired product but includes slightly more purification *via* automated column chromatography (CH_2_Cl_2_/MeOH). Furthermore, the introduction on the complex appears to be quantitative in the isolated pure product. As the chosen naphthalene diimide-based halide was synthesized separately, the boronic acid derivatives must be synthesized as well. Thus, the usage of the NDI-boronic acid ester and the bromo-decorated complex would require the same number of steps. The almost quantitative conversion and simple purification processes (precipitation and washing) of the boronic acid pinacol ester on the complex and after the aryl introduction represent clear advantages to introduce the boronic ester on the complex instead of performing such modifications on the ligands and then the complexation step(s).

In contrast to the electron-poor substrate NDIPhI, the respective Suzuki–Miyaura cross-coupling was performed with the electron-rich triarylamine-derivative TARABr (TARABr is 4-bromo-4′,4″-dimethyltriphenylamine) as the opposite extreme. It yielded only low amounts (35%, Scheme 6b) and required additional column chromatography for isolation. Nevertheless, column chromatography was performed with an automated flash system and managed to separate the product species with organic eluents (CH_2_Cl_2_/MeOH) instead of aqueous systems for respective classical route syntheses (*vide supra*). Again, the isolated product was assigned by exchange of characteristic signals in the ^1^H NMR spectrum (Figure 2), as the signal for the boronic ester disappears and the respective signals for TARA (4*H*, 2*H*, and 4*H*, blue box) emerge. We assign the contrary behavior of NDIPhI and TARABr by the lowered rate of oxidative addition for electron-rich *vs* electron-neutral/-poor substrates^22^ and for bromides compared to iodides.^23^

Subsequently, the alkyne of CCTIPS/TARA was deprotected with fluoride (Scheme 6c), followed by a Sonogashira cross-coupling with NDIPhI. The reaction performed yielded the desired product CCPhNDI/TARA in 78% yield. The product formation is also supported by the ^1^H NMR spectroscopy data (Figure 2), *i.e.*, the signal for TIPS disappears, while the signal for NDI appears (green box). Again, flash column chromatography on silica with organic solvent mixtures (CH_2_Cl_2_/MeOH) was sufficient to isolate the product (beside precipitation). In contrast, a similar reaction with TARABr starting from the PhNDI/CCTIPS complex does not lead to the desired isolated PhNDI/CCTARA. Again, we explain this by the lowered reactivity of electron-rich bromides *vs* electron-poor iodides in cross-coupling reactions (*vide supra*).

### Photophysical Characterization

**Figure 3:**
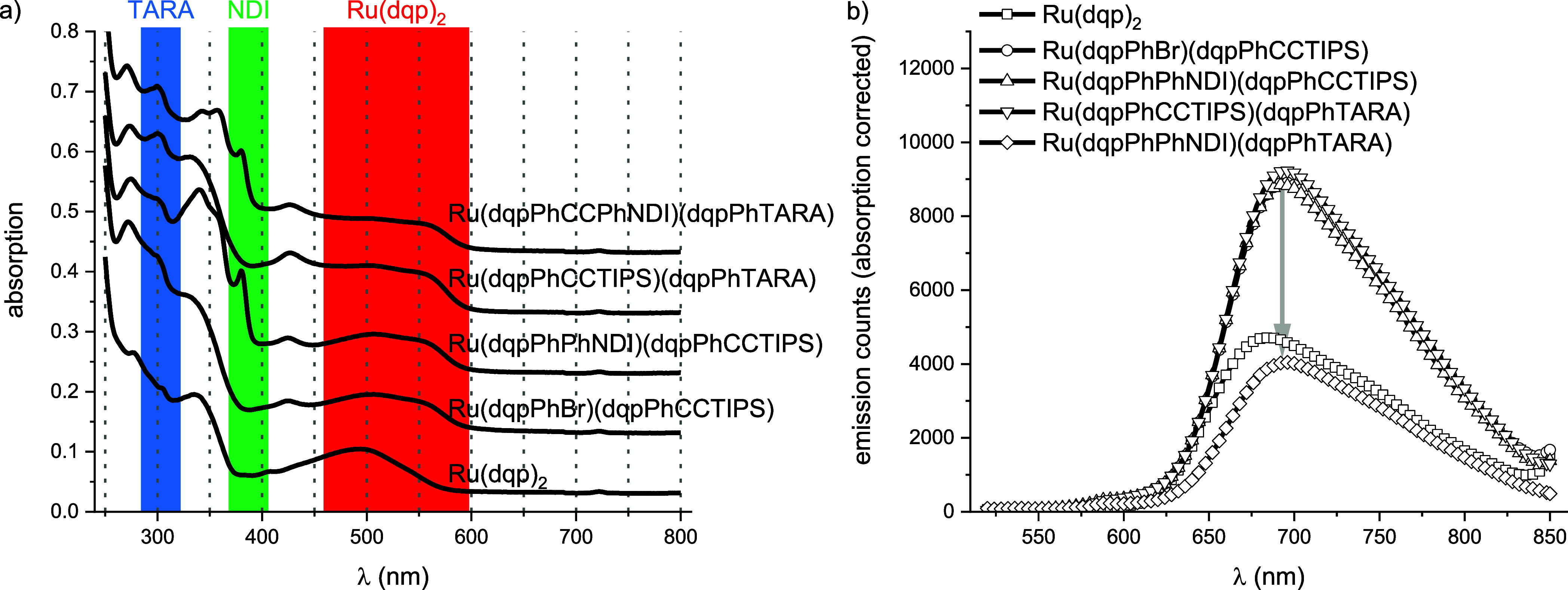
(a) Stacked absorption spectra of selected [Ru(dqp)_2_]^2+^-based complexes in dry CH_2_Cl_2_. The blue box marks characteristic TARA-signals, the green box marks characteristic NDI-signals, and the red box indicates characteristic [Ru(dqp)_2_]^2+^-based MLCT bands. (b) Emission spectra of selected [Ru(dqp)_2_]^2+^-based complexes with excitation at 500 nm in dry CH_2_Cl_2_. The gray arrow indicates the emission quenching for the triad [Ru(dqpPhCCPhNDI)(dqpPhTARA)]^2+^ (CCPhNDI/TARA) compared with CCTIPS/TARA or CCTIPS/Br.

To verify the applicability of our building block for the synthesis of useful architectures, we tested the photophysical properties of the building block complex (CCTIPS/Br), the two descending dyads (CCTIPS/TARA and PhNDI/CCTIPS) and the triad (CCPhNDI/TARA) (Figure 3). For comparison the nonfunctionalized prototype [Ru(dqp)_2_]^2+^ is included, which is known to show less red-shifted absorption and emission.^5,13^ In case of all functionalized complexes, the absorption spectra reveal the characteristic ^1^MLCT bands from around 450 nm up to 600 nm (Figure 3a, red box), while the acceptor-containing dyads and triad reveal a characteristic band for NDI at 380 nm (green box),^3^ and the donor-containing ones show a characteristic band for TARA at 300 nm (blue box).^3^ Steady-state emission spectra were recorded from solutions of absorbance values of around 0.1 at 500 nm. The emission intensities were normalized according to the absorption intensities at 500 nm for comparison. In all cases, emission maxima were found around 695 nm. Interestingly, the parental CCTIPS/Br complex and the dyads exhibit virtually identical emission intensities, whereas the acceptor-photosensitizer-donor triad revealed a reduced emission of 55% (Figure 3b). This finding is tentatively assigned to the inherently low driving force for primary charge separation. Notably, previous studies on polymer-bound NDI acceptors showed efficient charge separation,^9^ but the assemblies differ markedly in terms of the linkage motif, the mutual spatial orientations, the number of potential acceptor sites and minimum distance between the closest acceptor site and the Ru core due to conformational freedom of the polymer backbone. In the meantime, Wenger and co-workers reported a detailed study on the oxidative quenching of a similar Ru(dqp)_2_-based assembly with a NDI acceptor, which showed only 6% of charge separation.^24^ Their findings corroborate our result and general interpretation, and thus, a more detailed study is in due course but beyond the scope of this study. Nonetheless, the much higher quenching extent of the triad with respect to the dyads indicates the formation of a charge-separated state.

## Conclusions

In this contribution, late-stage diversification *via* the chemistry-on-the-complex was explored starting from a single bromide-functionalized ligand (dqpPhBr) to produce a systematic series of heteroleptic ruthenium(II) complexes. In this modular approach, the interconversion by C–C cross-coupling reactions was investigated at the complex. The introduction of the bromo-group into the protected alkyne (dqpPhCCTMS) *via* Sonogashira cross-coupling proceeded smoothly but resulted in alkyne–alkyne homocoupling upon desilylation, attributed to residual trace amounts of copper. Alternatively, the interconversion of the bromo-group into the boronic acid pinacol ester (dqpPhB(pin)) *via* Suzuki–Miyaura cross-coupling proceeded in a high yield. Hence, a universal bifunctional [Ru(dqp)_2_]^2+^ complex was synthesized bearing a protected alkyne group (-PhCCTIPS) and a boronic acid ester group (-PhB(pin) and was utilized to introduce electron donor or acceptor units in the subsequent orthogonal chemistry-on-the-complex reactions.

In comparison to the conventional route *via* ligand modification and a final complexation step, the complementary chemistry-on-the-complex strategy revealed very high yields (up to 95%) and a significantly simplified purification protocol (aqueous anion exchange and precipitation in diethyl ether) instead of column chromatography onto silica gel with aqueous salt-containing mobile phases. These findings are beneficial to compete with the conventional route in terms of cost-effectiveness because Ru is introduced earlier in the synthesis scheme and large losses would be detrimental. The orthogonal diversification of [Ru(dqpPh**B(pin)**)(dqpPh**CCTIPS**)]^2+^ was shown by Suzuki–Miyaura cross-coupling with a 4-bromo-*N*,*N*-diphenylaniline, followed by deprotection of the alkyne and Sonogashira cross-coupling with an *N*-4-iodophenyl-naphthalene diimide. *Via* this route, a donor-photosensitizer-acceptor (D–P–A) triad was isolated by flash column chromatography with organic solvents, demonstrating the benefit *vs* manual column chromatography with salt-containing aqueous eluent mixtures that is typically required for any complexation step. An exemplarily photophysical characterization by steady state spectroscopy revealed preserved emission energies and quantum yields for dyads, while the corresponding triad showed a reduced emission by about 55%. More importantly, this methodology enables the systematic variation and photophysical characterization of donor and acceptor units to reveal the photophysical details. Hence, the chemistry-on-the-complex provides a robust complementary approach for the modular design and synthesis of D–P–A systems and beyond.

## Supporting Information Available

Detailed syntheses and instrumental details (SEC, ESI-ToF-MS, and NMR) (PDF)

## ABBREVIATIONS

B(pin): boronic acid pinacol ester
CH_2_Cl_2_: dichloromethane
CH_3_CN: acetonitrile
CV: column volume
DMF: *N*,*N*-dimethylformamide
dppf: 1,1′-bis(diphenylphosphino)ferrocene
dqpPhR: 2,6-di(quinolin-8-yl)-4-(4′-*R*-phenyl)-pyridine
L Pd G3: Buchwald’s third-generation catalyst, where L is the phosphine
Na_2_-EDTA: ethan-1,2-diyldiamine-*N,N,N′,N*′**-tetraacetic acid
NDIPhI: *N*-(4-iodophenyl)-*N′*-(2′-ethylhexyl)-naphthalene-1,4,5,8-tetracarboxylic acid diimide
NEt_3_: triethylamine
PPh_3_: triphenylphosphine
RuPhos: dicyclohexyl-(2′,6′-diisopropoxy-(2,1′-biphenyl))phosphine
SPhos: 2′,6′-dimethoxybiphen-1-yl-di(cyclohexyl)phosphine
TARA-Br: triarylamine 4-bromo-*N*,*N*-di(4′-tolyl)aniline
TBAF: tetrabutylammonium fluoride
THF: tetrahydrofuran
TIPS: tri(*iso*-propyl)silyl
TMS: trimethylsilyl
