# Supplementary material for: Exploiting Orthogonal C–C Cross-Coupling Reactions for Chemistry-on-the-Complex: Modular Assembly of 2,6-Di(quinolin-8-yl)pyridine Ruthenium(II) Photosensitizer Triads
Source: Inorg Chem. 2024 Feb 19;63(9):4053–62. doi: 10.1021/acs.inorgchem.3c03380 (PMC10915800; doi:10.1021/acs.inorgchem.3c03380)
Supplement: Supplementary file 1 — ic3c03380_si_001.pdf [file ic3c03380_si_001.pdf]

Supporting Information

Exploiting Orthogonal C-C Cross-Coupling Reactions for *Chemistry-on-the-Complex:* Modular Assembly of 2,6-Di(quinoline-8-yl)pyridine Ruthenium(II) Photosensitizer Triads

Alexander Kleine,[a] *Ulrich S. Schubert,*[a,b,]* *and Michael Jäger*[a,b,]*

[a]Laboratory of Organic and Macromolecular Chemistry (IOMC), Friedrich Schiller University Jena, 07743 Jena, Germany.

[b] Center for Energy and Environmental Chemistry Jena (CEEC Jena), Friedrich Schiller University Jena, 07743 Jena, Germany.

Corresponding Authors:
Ulrich S. Schubert − Email: ulrich.schubert@uni-jena.de
Michael Jäger − Email: michael.jager.iomc@uni-jena.de

Content

Instrumentation 3
Materials 3
Synthetic procedures 3
NMR data 12
ESI-ToF MS data 26
SEC data 34
Absorption and emission spectra 42
References 46

Instrumentation

SEC was performed utilizing a Shimadzu series 10 SCL-10A vp, equipped with degasser (DGU-14A), pump (LC-10AD vp), autosampler (SIL-10AD vp), overn (CTO-10AC vp), and RI- (RID-10A) and PDA-detector (SPD-10MA vp). The column used is a phenomenex phenogel (100,000 Å/1,000 Å, 10 µm particle size) and DMAc + 0.08 wt% NH_4PF_6 was used as eluent.

NMR spectra were recorded on a 400 MHz Bruker ACANCE III NMR spectrometer equipped with a BBO or BBFO probe, on a 300 MHz Bruker Fourier NMR spectrometer equipped with Dual $^1H/^{13}C$ probe, or on a 600 MHz Bruker Avance III NMR spectrometer equipped with a TCPI probe. All samples were measured in deuterated solvents at 300 K. Chemical shifts are reported in parts per million (ppm, δ scale) relative to the residual solvent signal.

ESI-ToF mass spectrometry was performed on a Bruker micrOTOF QII, or a timsTOF mass spectrometer by syringe injection.

Absorption spectroscopy was performed in 10×10 mm Hellma Analytics quartz glass cuvettes on a Perkin Elmer Lambda 750 in dry solvents against blank samples.

Emission spectroscopy was performed in 10×10 mm Hellma Analytics quartz glass cuvettes on a Jasco FP-8300 fluorescence spectrometer equipped with an ETC-815 Peltier thermostatted single cell holder after calibration with a Jasco ESC-142 calibrated WI Light Source and Jasco VDK-140 Validaiton Kit 1 in dry solvents.

Materials

All chemicals and solvents were purchased from abcr, Acros Organics, Alfa Aesar, Apollo Scientific, Fluka, fluorochem, Sigma-Aldrich, TCI, or VWR and used without further purification if not stated otherwise.

4-(4'-Bromophenyl)-2,6-diquinoline-8'-diylpyridine,[1] 4-(4'-(triisopropylsilyl-ethynyl)phenyl)-2,6-diquinoline-8'-diylpyridine,[2] [(iPrSPh)$_2$(MeOH)RuCl$_3$],[3] [Ru(dqpPhBr)(CH$_3$CN)$_3$]$^{2+}$ (starting from [(iPrSPh)$_2$(MeOH)RuCl$_3$]),[4] 4-(azidomethyl)phenylboronic acid pinacol ester,[5] and RuPhos Pd G3[6] were prepared according to literature procedures. dqpPhCCTMS and [Ru(dqpPhCCTIPS)(CH$_3$CN)$_3$]$^{2+}$ were already investigated by Lennart Skodda during his bachelor thesis which is not publicly available. Thus, both substances are incorporated here with permission.

Note, that dicationic species carry two PF_6^- anions which are not shown throughout for clarity.

Synthetic procedures

N-(2'-Ethylhexyl)-*N*'-(4'-iodophenyl)-naphthalene-1,4,5,8-tetracarboxylic diimide (**NDIPhI**).

Synthesis according to an adopted literature procedure.[7]

Naphthalene-1,4,5,8-tetracarboxylic acid dianhydride (0.80 g, 2.98 mmol) and 2-ethylhexylamine (0.49 mL, 2.99 mmol) were combined in a microwave vial. The vial was sealed and dry DMF (20 mL) was added. The vial was purged with a nitrogen flow for 10 min and for 1 min subjected to ultrasound sonification. The vial was placed in the microwave reactor and heated for 5 min at 75 °C, then 15 min at 140 °C. After the vial cooled to room temperature, the solvent was evaporated, the crude product was suspended in acetone (15 mL) and added dropwise in stirred hydrochloric acid (aqueous, 1 M, 150 mL). The precipitate was filtered off and washed with H_2O (3×75 mL) to yield 1.725 g of the crude monoimide. The crude monoimide (0.804 g, 2.119 mmol) and 4-iodoaniline (0.467 g, 2.132 mmol) were

combined in a microwave vial. The vial was sealed and the atmosphere inside replaced with nitrogen. Dry DMF (20 mL) and dry NEt_3 (0.3 mL, 2.152 mmol) were added, and the mixture stirred and subjected to ultrasound sonification yielding to dissolution of most particles. The vial was placed in the microwave reactor and heated to 40 °C for 5 min, then 140 °C for 15 min. After the vial cooled to room temperature, the solvent was evaporated, and the crude product was suspended in acetone (15 mL). The mixture was added dropwise in stirred hydrochloric acid (aqueous, 1 M, 150 mL), washed with H_2O, and dried under reduced pressure. The crude product was dissolved in CH_2Cl_2 and dried over Na_2SO_4. The solution was filtered, and the solvent was evaporated. The crude product was subjected to flash column chromatography (120 g SiOH-cartridge, hexane/CH_2Cl_2 50:50 to 10:90) to yield the isolated bright yellow solid (0.248 g, 20%).

^{1}H-NMR (400 MHz, CD_2Cl_2): δ = 8.78 (m, 4*H*, NDI-*H*), 7.90 (d, 2*H*, Ph-*H*, *J* = 8.47 Hz), 7.08 (d, 2*H*, Ph-*H*, *J* = 8.57 Hz), 4.15 (m, 2*H*, -NCH_2-), 1.95 (m, 1*H*, -C*H*-), 1.50-1.20 (m, 8*H*, -CH_2-), 0.94 (t, 3*H*, -CH_3, *J* = 7.41 Hz), 0.88 (t, 3*H*, -CH_3, *J* = 7.01 Hz) ppm.

^{13}C-APT-NMR (100 MHz, CD_2Cl_2): δ = 163.17 (s, 2*C*, -*C*(O)-), 162.90 (s, 2*C*, -*C*(O)-), 138.90 (s, 2*C*, Ph*C*H), 134.43 (s, 1*C*, Ph*C*), 131.61 (s, 2*C*, NDI*C*H), 131.22 (s, 2*C*, NDI*C*H), 130.57 (s, 2*C*, Ph*C*H), 127.15 (s, 2*C*, NDI*C*), 126.97 (s, 2*C*, NDI*C*), 126.53 (s, 2*C*, NDI*C*), 95.11 (s, 1*C*, Ph*C*), 44.81 (s, 1*C*, -N*C*H_2-), 38.06 (s, 1*C*, -*C*H-), 30.82 (s, 1*C*, -*C*H_2-), 28.75 (s, 1*C*, -*C*H_2-), 24.17 (s, 1*C*, -*C*H_2-), 23.16 (s, 1*C*, -*C*H_2-), 14.21 (s, 1*C*, -*C*H_3-), 10.72 (s, 1*C*, -*C*H_3-) ppm.

Elemental analysis ($C_{28}H_{25}IN_2O_4$): calc.: 57.94% C, 4.34% H, 4.83% N; found: 58.00% C, 4.31% H, 4.59% N.

HR-ESI-MS ($[C_{28}H_{25}IN_2O_4+K]^+$): calc.: 619.0491; found: 619.0464; error: 4.3 ppm.

4-(4’-(Trimethylsilyl-ethynyl)phenyl)-2,6-diquinoline-8’-diylpyridine (**dqpPhCCTMS**).

Synthesis according to an adopted literature procedure.[8]

dqpPhBr (1.00 g, 2.05 mmol), $Pd(PPh_3)_4$ (0.12 g, 0.10 mmol), and copper(I) iodide (0.02 g, 0.08 mmol) were combined in a flask equipped with reflux condenser and the atmosphere was replaced by nitrogen. Dry THF (20 mL) and triethylamine (10 mL) were added before addition of trimethylsilyl acetylene (0.60 g, 6.11 mmol). The mixture was placed in a pre-heated oil bath at 70 °C for 19 h. After cooling to room temperature, the mixture was poured on H_2O (25 mL) and extraction was performed with CH_2Cl_2 (3×25 mL). The combined organic phases were dried over Na_2SO_4, filtered, and the solvent of the filtrate evaporated under reduced pressure. The crude product was purified via flash column chromatography (120 g SiOH-cartridge, CH_2Cl_2/EtOAc 80:20) to yield the product (0.46 g, 45%).

^{1}H-NMR (300 MHz, $CDCl_3$): δ = 9.01 (dd, 2*H*, qu*H*, $^3J_{H\text{-}H}$ = 4.16 Hz, $^4J_{H\text{-}H}$ = 1.82 Hz), 8.31 (s, 2*H*, py*H*), 8.30-8.28 (dd, 2*H*, qu*H*), 8.25 (ds, 2*H*, qu*H*), 7.90 (dd, 2*H*, qu*H*, $^3J_{H\text{-}H}$ = 8.17 Hz, $^4J_{H\text{-}H}$ = 1.44 Hz), 7.78 (d, 2*H*, Ph*H*, $^3J_{H\text{-}H}$ = 8.47 Hz), 7.68 (m, 2*H*, qu*H*), 7.58 (d, 2*H*, Ph*H*, $^3J_{H\text{-}H}$ = 8.44 Hz), 7.46 (dd, 2*H*, Ph*H*, $^3J_{H\text{-}H}$ = 8.29 Hz, $^4J_{H\text{-}H}$ = 4.17 Hz), 0.26 (s, 9*H*, -Si(CH_3)$_3$) ppm.

^{13}C-NMR (100 MHz, $CDCl_3$): δ = 157.57 (s, 2*C*, py*C*), 150.45 (s, 2*C*, qu*C*H), 146.30 (s, 1*C*, Ph*C*), 146.12 (s, 2*C*, qu*C*), 139.48 (s, 2*C*, qu*C*), 139.37 (s, 1*C*, py*C*), 136.54 (s, 2*C*, qu*C*H), 132.58 (s, 2*C*, Ph*C*H), 131.66 (s, 2*C*, qu*C*H), 128.79 (s, 1*C*, qu*C*H), 128.74 (s, 1*C*, qu*C*H), 127.49 (s, 2*C*, Ph*C*H), 126.72 (s, 2*C*, qu*C*H), 123.68 (s, 2*C*, py*C*H), 123.48 (s, 1*C*, Ph*C*), 121.14 (s, 2*C*, qu*C*H), 104.93 (s, 1*C*, -*C*CSi), 95.66 (s, 1*C*, -C*C*Si), 0.08 (s, 3*C*, -Si(*C*H_3)$_3$) ppm.

HR-ESI-MS ($[C_{34}H_{27}N_3Si+H]^+$): calc.: 506.2047; found: 506.2051; error: –0.8 ppm.

4-(4’-(Boronic acid pinacol ester)phenyl)-2,6-diquinoline-8’-diylpyridine (**dqpPhB(pin)**).

Synthesis according to an adopted literature procedure.[9]

dqpPhBr (0.150 g, 0.307 mmol), bis(pinakolato)diboron (0.087 g, 0.343 mmol), Pd(dppf)Cl_2 (6.8 mg, 9.3 µmol), and potassium acetate (0.091 g, 0.927 mmol) were combined in a microwave vial, dry DMSO was added (3 mL), the vial purged with a nitrogen flow (5 min), and the vial placed in a pre-heated oil

bath at 80 °C for 6 h. After cooling to room temperature, toluene (50 mL) was added and the organic phase washed with H_2O (5×40 mL). The organic phase was dried over Na_2SO_4 and the solvent evaporated yielding the pure product (0.139 g, 85%).

^{1}H-NMR (400 MHz, $CDCl_3$): δ = 8.99 (dd, 2*H*, qu*H*, *J* = 4.10 Hz, *J* = 1.66 Hz), 8.32 (s, 2*H*, py*H*), 8.27 (dd, 2*H*, qu*H*, *J* = 7.16 Hz, *J* = 1.24 Hz), 8.24 (dd, 2*H*, qu*H*, *J* = 8.28 Hz, *J* = 1.52 Hz), 7.94 (d, 2*H*, Ph*H*, *J* = 8.08 Hz), 7.89 (d, 2*H*, qu*H*, *J* = 8.12 Hz), 7.83 (d, 2*H*, Ph*H*, *J* = 8.08 Hz), 7.67 (dd, 2*H*, qu*H*, *J* = 7.66 Hz), 7.45 (dd, 2*H*, qu*H*, *J* = 8.26 Hz, *J* = 4.15 Hz), 1.36 (s, 12*H*, -C*H*$_3$) ppm.

^{13}C-APT-NMR (100 MHz, $CDCl_3$): δ = 157.57 (s, 2*C*, py*C*), 150.46 (s, 2*C*, qu*C*H), 147.17 (s, 1*C*, Ph*C*), 146.20 (s, 2*C*, qu*C*), 142.12 (s, 1*C*, Ph and/or py*C*), 139.67 (s, 2*C*, qu*C*), 136.51 (s, 2*C*, qu*C*H), 135.44 (s, 2*C*, Ph*C*H), 131.66 (s, 2*C*, qu*C*H), 128.82 (s, 2*C*, qu*C*), 128.68 (s, 2*C*, qu*C*H), 127.05 (s, 2*C*, Ph*C*H), 126.73 (s, 2*C*, qu*C*H), 123.96 (s, 2*C*, py*C*H), 121.15 (s, 2*C*, qu*C*H), 84.05 (s, 2*C*, -BO*C*-), 25.02 (s, 4*C*, -*C*H$_3$) ppm.

HR-ESI-MS ($[C_{35}H_{30}BN_3O_2+H]^{2+}$): calc.: 536.2504 m/z; found: 536.2489 m/z; error: 3.9 ppm.

4-(4’-(Trimethylsilyl-ethynyl)phenyl)-2,6-diquinoline-8’-diylpyridine (**dqpPhPhNDI**).

dqpPhB(pin) (33.2 mg, 0.062 mmol), **NDIPhI** (36.2 mg, 0.062 mmol), SPhos Pd G3 (8.5 mg, 0.011 mmol), SPhos (48 mg, 0.028 mmol), and potassium phosphate (106.7 mg, 0.503 mmol) were combined in a microwave vial and the atmosphere inside was replaced with nitrogen. Dry toluene (2 mL) and H_2O (0.1 mL) were added and the vial was placed in a pre-heated oil bath (50 °C) under stirring overnight. After cooling to room temperature, the reaction mixture was put on H_2O (25 mL) and extracted with CH_2Cl_2 (2×25 mL). The combined organic phases were washed with brine (50 mL), dried over Na_2SO_4, filtered and the solvent was evaporated. The crude product was subjected to flash column chromatography (25 g SiOH-cartridge, CH_2Cl_2/MeOH 96:3, then gradient to 50:50) yielding the pure product (46 mg, 86%).

^{1}H-NMR (300 MHz, $CDCl_3$): δ = 9.04 (dd, 2*H*, qu*H*, *J* = 4.17 Hz, *J* = 1.77 Hz), 8.84 (d, 2*H*, NDI*H*, *J* = 7.62 Hz), 8.80 (d, 2*H*, NDI*H*, *J* = 7.65 Hz), 8.41 (s, 2*H*, py*H*), 8.34 (dd, 2*H*, qu*H*, *J* = 7.17 Hz, *J* = 1.38 Hz), 8.27 (dd, 2*H*, qu*H*, *J* = 8.32 Hz, *J* = 1.71 Hz), 7.96 (d, 2*H*, Ph*H*, *J* = 8.32 Hz), 7.92 (dd, 2*H*, qu*H*, *J* = 8.18 Hz, *J* = 1.34 Hz), 7.85 (d, 2*H*, Ph*H*, *J* = 8.44 Hz), 7.79 (d, 2*H*, Ph*H*, *J* = 8.35 Hz), 7.70 (t, 2*H*, qu*H*, *J* = 7.67 Hz), 7.48 (dd, 2*H*, qu*H*, *J* = 8.27 Hz, *J* = 4.19 Hz), 7.43 (d, 2*H*, Ph*H*, *J* = 8.47 Hz), 4.17 (m, 2*H*, -NC*H*$_2$-), 1.97 (m, 1*H*, -C*H*-), 1.55-1.12 (m, 8*H*, -C*H*$_2$-), 0.96 (t, 3*H*, -C*H*$_3$, *J* = 7.40 Hz), 0.89 (t, 3*H*, -C*H*$_3$, *J* = 7.02 Hz) ppm.

^{13}C-APT-NMR (100 MHz, $CDCl_3$): δ = 163.28 (s, 2*C*, -*C*(O)-), 163.21 (s, 2*C*, -*C*(O)-), 157.44 (s, 2*C*, py*C*), 150.47 (s, 2*C*, qu*C*H), 146.72 (s, 1*C*, Ph*C*), 146.17 (s, 2*C*, qu*C*), 141.71 (s, 1*C*, Ph*C*), 140.54 (s, 1*C*, Ph*C*), 139.48 (s, 2*C*, qu*C*), 138.89 (s, 1*C*, py*C*), 136.57 (s, 2*C*, qu*C*H), 134.01 (s, 1*C*, Ph*C*), 131.73 (s, 2*C*, qu*C*H), 131.57 (s, 2*C*, NDI*C*H), 131.23 (s, 2*C*, NDI*C*H), 129.02 (s, 2*C*, Ph*C*H), 128.83 (s, 2*C*, qu*C*), 128.75 (s, 2*C*, qu*C*H), 128.44 (s, 2*C*, Ph*C*H), 128.26 (s, 2*C*, qu*C*H), 127.95 (s, 2*C*, Ph*C*H), 127.20 (s, 2*C*, NDI*C*), 127.09 (s, 2*C*, qu*C*), 127.00 (s, 2*C*, NDI*C*), 126.77 (s, 2*C*, qu*C*H), 126.75 (s, 2*C*, NDI*C*), 123.85 (s, 2*C*, py*C*H), 121.16 (s, 2*C*, qu*C*H), 44.82 (s, 1*C*, -O*C*H$_2$-), 38.08 (s, 1*C*, -*C*H-), 30.85 (s, 1*C*, -*C*H$_2$-), 28.77 (s, 1*C*, -*C*H$_2$-), 24.19 (s, 1*C*, -*C*H$_2$-), 23.17 (s, 1*C*, -*C*H$_2$-), 14.21 (s, 1*C*, -*C*H$_3$), 10.74 (s, 1*C*, -*C*H$_3$) ppm.

HR-ESI-MS ($[C_{57}H_{43}N_5O_4+H]^+$): calc. 862.3388 m/z; found: 862.3387 m/z; error: 0.1 ppm.

[Ru(dqpPhCCTIPS)(CH_3CN)$_3$]$^{2+}$.

Synthesis according to an adopted literature procedure.[10]

[(iPrSPh)$_2$(MeOH)RuCl$_3$] (0.27 g, 0.51 mmol), **dqpPhCCTIPS** (0.29 g, 0.50 mmol), and CH_3CN (6 mL) were combined in a microwave vial. The flask was sealed with a rubber septum and the mixture purged with a nitrogen flow for 10 min. The vial was placed in a microwave reactor at 120 °C for 10 h. After cooling to room temperature, H_2O (2 mL), EtOH (4 mL), and $AgNO_3$ (0.27 g, 1.56 mmol) were added and the sealed vial was placed in a pre-heated oil bath at 80 °C. for 25 h. After cooling to room temperature, the mixture was filtered over a Celite plug, and the Celite washed with CH_3CN (15 mL). After

evaporation of the solvent, the crude product was purified by column chromatography (SiOH, $CH_3CN/H_2O/KNO_3$ (aq., sat.) 40:4:1). After solvent evaporation, $NaPF_6$ (aq., 10 g/L, 100 mL) was added and the aqueous layer extracted with CH_2Cl_2 (100 mL, then 2×30 mL). The combined organic phases were washed with H_2O, dried over Na_2SO_4, filtered, and the solvent was evaporated yielding the yellow-brown solid (0.40 g, 74%).

^{1}H-NMR (300 MHz, CD_3CN): δ = 9.09 (dd, 2*H*, qu*H*, $^3J_{H\text{-}H}$ = 5.15 Hz, $^4J_{H\text{-}H}$ = 1.43 Hz), 8.69 (dd, 2*H*, qu*H*, $^3J_{H\text{-}H}$ = 7.46 Hz, $^4J_{H\text{-}H}$ = 1.13 Hz), 8.62 (dd, 2*H*, qu*H*, $^3J_{H\text{-}H}$ = 8.30 Hz, $^4J_{H\text{-}H}$ = 1.40 Hz), 8.29 (dd, 2*H*, qu*H*, $^3J_{H\text{-}H}$ = 8.17 Hz, $^4J_{H\text{-}H}$ = 0.99 Hz), 8.20 (s, 2*H*, py*H*), 7.94 (m, 4*H*, qu*H*+Ph*H*), 7.68 (m, 4*H*, qu*H*+Ph*H*), 2.45 (s, 3*H*, NCC*H*$_3$), 1.96 (s, 6*H*, NCC*H*$_3$), 1.16 (s, 21*H*, -Si(C*H*(C*H*$_3$)$_2$)$_3$) ppm.

^{13}C-APT-NMR (100 MHz, CD_3CN): δ = 159.70 (s, 2*C*, qu*C*H), 158.53 (s, 2*C*, qu*C*), 149.99 (s, 1*C*, Ph*C*), 147.23 (s, 2*C*, qu*C*), 139.56 (s, 2*C*, qu*C*H), 136.91 (s, 1*C*, py*C*), 135.26 (s, 2*C*, qu*C*), 135.04 (s, 2*C*, qu*C*H), 133.55 (s, 2*C*, qu*C*H or Ph*C*H), 132.90 (s, 2*C*, qu*C*H), 129.55 (s, 2*C*, py*C*), 129.06 (s, 2*C*, qu*C*H), 128.67 (s, 2*C*, qu*C*H or Ph*C*H), 127.72 (s, 1*C*, N*C*CH$_3$), 127.27 (s, 2*C*, N*C*CH$_3$), 126.25 (s, 2*C*, py*C*H), 126.02 (s, 1*C*, Ph*C*), 123.14 (s, 2*C*, qu*C*H or Ph*C*H), 107.18 (s, 1*C*, -*C*CSi), 94.24 (s, 1*C*, -C*C*Si), 18.93 (s, 6*C*, -Si(CH(*C*H$_3$)$_2$)$_3$), 12.03 (s, 3*C*, -Si(*C*H(CH$_3$)$_2$)$_3$), 4.42 (s, 1*C*, NC*C*H$_3$), 3.72 (s, 2*C*, NC*C*H$_3$) ppm.

HR-ESI-MS ($[C_{46}H_{48}N_6RuSi]^{2+}$): calc.: 407.1371; found: 407.1382; error: –1.2 ppm.

$[Ru(dqpPhBr)(dqpPhCCTIPS)]^{2+}$.

Synthesis according to an adopted literature procedure.[10]

AK-235 (500 mg, 0.499 mmol) and dqpPhCCTIPS (295 mg, 0.500 mmol) combined in a microwave vial. 15 mL DMF added, vial sealed and purged with a nitrogen flow for 10 min and placed in a pre-heated oil bath at 120 °C. After following the reaction progress by ^{1}H-NMR, the mixture was cooled to room temperature after 2 days. Mixture put on $NaPF_6$ (aq., 5 g/L, 100 mL) and extracted with CH_2Cl_2 (100 mL, then 3×50 mL). Organic phases were combined, washed with H_2O (200 mL), dried over Na_2SO_4 and filtered before evaporation of the solvent. The crude product was subjected to column chromatography (SiOH, $CH_3CN/H_2O/KNO_{3(aq.,sat.)}$ 40:4:1). After combination and solvent evaporation of clean fractions, CH_2Cl_2 (25 mL) was added and washed with $NaPF_6$ (aq., 5 g/L, 25 mL) and H_2O (25 mL). The organic layer was dried over Na_2SO_4, filtered, and the solvent evaporated to yield the clean product (144 mg, 20%). Further product containing fractions were subjected to later crystallization.

^{1}H-NMR (400 MHz, CD_3CN): δ = 8.14 (m, 4*H*, qu*H*), 8.11 (m, 4*H*, py*H*), 8.10-8.06 (m, 4*H*, qu*H*), 7.95 (d, 2*H*, Ph*H*, *J* = 8.46 Hz), 7.92-7.88 (m, 4*H*, qu*H*), 7.87 (d, 2*H*, Ph*H*, *J* = 8.93 Hz), 7.77 (d, 2*H*, Ph*H*, *J* = 8.60 Hz), 7.71 (d, 4*H*, qu*H*, *J* = 8.10 Hz), 7.67 (d, 2*H*, Ph*H*, *J* = 8.48 Hz), 7.49 (t, 4*H*, qu*H*, *J* = 7.80 Hz), 7.11-7.05 (m, 4*H*, qu*H*), 1.16 (m, 21*H*, -SiC*H*(C*H*$_3$)$_2$) ppm.

^{13}C-APT-NMR (100 MHz, CD_3CN): δ = 159.52 (s, 4*C*, qu*C*H), 158.11 (s, 4*C*, qu*C*), 149.55 (s, 1*C*, py*C*), 149.41 (s, 1*C*, py*C*), 147.56 (s, 4*C*, qu*C*), 138.57 (s, 4*C*, qu*C*H), 137.01 (s, 1*C*, Ph*C*), 136.18 (s, 1*C*, Ph*C*), 134.48 (s, 2*C*, qu*C*H), 134.46 (s, 2*C*, qu*C*H), 133.58 (s, 2*C*, Ph*C*H), 133.42 (s, 2*C*, Ph*C*H), 132.81 (s, 2*C*, py*C*), 132.77 (s, 2*C*, py*C*), 131.63 (s, 4*C*, qu*C*H), 130.34 (s, 2*C*, Ph*C*H), 128.66 (s, 2*C*, Ph*C*H), 127.83 (s, 4*C*, qu*C*H), 127.57 (s, 4*C*, qu*C*), 126.22 (s, 2*C*, py*C*H), 126.20 (s, 2*C*, py*C*H), 125.99 (s, 1*C*, Ph*C*), 125.35 (s, 1*C*, Ph*C*), 123.07 (s, 4*C*, qu*C*H), 107.21 (s, 1*C*, -SiC*C*-), 94.32 (s, 1*C*, -SiC*C*-), 18.94 (s, 6*C*, -*C*H$_3$), 12.05 (s, 3*C*, -*C*H-) ppm.

HR-ESI ($[C_{69}H_{57}BrN_6RuSi]^{2+}$): calc.: 589.1315 m/z, found: 589.1321 m/z; error: 0.0 ppm.

$[Ru(dqpPhBr)(dqpPhPhNDI)]^{2+}$.

Synthesis according to an adopted literature procedure.[10]

$[Ru(dqpPhBr)(CH_3CN)_3]^{2+}$ (AK-235) (41.4 mg, 0.041 mmol) and **dqpPhPhNDI** (44.5 mg, 0.052 mmol) were combined in a microwave vial, and DMF (1 mL) was added. The vial was sealed, and purged with a nitrogen flow for 10 min. Placed in a pre-heated oil bath at 120 °C for 2 days. Cooled to room temperature, put on 50 mL KPF_6 (aq., 5 g/mL, 50 mL), and extracted with CH_2Cl_2 (50 mL), then

CH_2Cl_2/MeOH (90:10, 10 mL). Combined organic phases washed with H_2O (50 mL), and aqueous phase again extracted with CH_2Cl_2/MeOH (90:10, 50 mL). Organic phases were combined and the solvent evaporated. The crude product was dissolved in CH_3CN (4 mL) and dropwised in diethyl ether (40 mL). The precipitate was washed with toluene (3×20 mL), then diethyl ether (3×20 mL). The precipitate was flushed from the frit with CH_2Cl_2 and the solvent evaporated. After ineffective crystallization attempts (in refluxed CH_3CN/toluene 1:10), the crude product was subjected to column chromatography (SiOH, $CH_3CN/H_2O/KNO_{3(aq.,sat.)}$ 40:4:1). After combination of fractions containing clean product, the solvents were evaporated, CH_2Cl_2 (100 mL) added, and the organic phase washed with KPF_6 (aq., 5 g/L, 100 mL), then H_2O (100 mL). After solvent evaporation, the clean product was obtained (36 mg, 50%).

^{1}H-NMR (400 MHz, CD_2Cl_2): δ = 8.78 (m, 4*H*, NDI*H*), 8.13-8.03 (m, 10*H*, qu*H*+py*H*), 8.00 (s, 2*H*, py*H*), 7.98 (d, 2*H*, Ph*H*, *J* = 8.16 Hz), 7.90 (d, 2*H*, Ph*H*, *J* = 8.04 Hz), 7.88-7.80 (m, 6*H*, qu*H*+Ph*H*), 7.75-7.65 (m, 8*H*, qu*H*+Ph*H*), 7.58-7.50 (m, 4*H*, qu*H*), 7.49-7.42 (m, 2*H*, Ph*H*), 7.20-7.11 (m, 4*H*, qu*H*), 4.14 (m, 2*H*, -OC*H*$_2$-), 1.95 (m, 1*H*, -C*H*-), 1.48-1.18 (m, 8*H*, -C*H*$_2$-), 0.95 (m, 3*H*, -C*H*$_3$), 0.90 (m, 3*H*, -C*H*$_3$) ppm. Signals at 7.76 (d, 0.6*H*) and 7.40 (d, 0.6*H*) ppm are remaining impurities fitting to a 1,4-substituted benzene.

^{13}C-APT-NMR (100 MHz, CD_2Cl_2): δ = 163.51 (s, 4*C*, -*C*(=O)), 158.39 (s, 2*C*, qu*C*H), 158.32 (s, 2*C*, qu*C*H), 157.33 (s, 2*C*, qu*C*), 157.25 (s, 2*C*, qu*C*), 157.19 (s, x*C*, Ar*C*), 150.47 (s, 1*C*, Ph*C*), 149.91 (s, 1*C*, Ph*C*), 146.98 (s, 4*C*, qu*C*), 142.63 (s, 1*C*, Ph*C*), 140.79 (s, 1*C*, Ph*C*), 138.35 (s, 2*C*, py*C*H), 135.45 (s, 2*C*, Ar*C*), 135.37 (s, 2*C*, Ar*C*), 135.21 (s, 2*C*, py*C*), 133.58 (s, 2*C*, qu*C*H), 133.48 (s, 2*C*, qu*C*H), 133.09 (s, 2*C*, Ph*C*H), 132.19 (s, 2*C*, Ar*C*), 132.10 (s, 2*C*, Ar*C*), 131.57 (s, *C*, Ar*C*H), 131.43 (s, *C*, Ar*C*H), 131.28 (s, *C*, Ar*C*H), 129.66 (s, x*C*, Ar*C*H), 129.50 (s, x*C*, Ar*C*H), 129.36 (s, x*C*, Ar*C*H), 129.13 (s, x*C*, Ar*C*H), 128.73 (s, x*C*, Ar*C*H), 128.48 (s, x*C*, Ar*C*H), 128.43 (s, x*C*, Ar*C*H), 128.31 (s, x*C*, Ar*C*H), 128.16 (s, x*C*, Ar*C*H), 127.66 (s, x*C*, Ar*C*H), 127.63 (s, x*C*, Ar*C*H), 127.53 (s, 2*C*, Ar*C*), 127.49 (s, 2*C*, Ar*C*), 127.44 (s, 2*C*, Ar*C*), 127.30 (s, 2*C*, Ar*C*), 127.16 (s, 2*C*, Ar*C*), 127.14 (s, 2*C*, Ar*C*), 126.93 (s, 2*C*, Ar*C*), 126.86 (s, *C*, Ar*C*H), 126.00 (s, *C*, Ar*C*H), 125.94 (s, 2*C*, py*C*H), 125.82 (s, *C*, Ar*C*H), 125.51 (s, 2*C*, Ar*C*), 122.83 (s, 2*C*, qu*C*H), 122.75 (s, 2*C*, qu*C*H), 44.84 (s, 1*C*, -*C*H$_2$-), 38.34 (s, 1*C*, -CH-), 31.03 (s, 1*C*, -*C*H$_2$-), 30.06 or 29.00 (s, 1*C*, -*C*H$_2$-), 24.35 (s, 1*C*, -*C*H$_2$-), 23.44 (s, 1*C*, -*C*H$_2$-), 14.25 (s, 1*C*, -*C*H$_3$), 10.73 (s, 1*C*, -*C*H$_3$) ppm.

HR-ESI-MS ($[C_{86}H_{61}BrN_8O_4Ru]^{2+}$): calc.:726.1516 m/z; found: 726.1509 m/z; error: 2.3 ppm.

[Ru(dqpPhCCTMS)(dqpPhCCTIPS)]$^{2+}$.

Synthesis according to an adopted literature procedure.[2]

[Ru(dqpPhBr)(dqpPhCCTIPS)]$^{2+}$ (134 mg, 0.09 mmol), $Pd(PPh_3)_4$ (41 mg, 35 µmol), and copper(I) iodide (7 mg, 39 µmol) were combined in a vial. DMF (9 mL) and triethylamine (9 mL) were added, the vial was sealed and the mixture was purged with a nitrogen flow for 10 min. Trimethylsilyl acetylene (40 µL, 0.28 mmol) was added and the mixture placed in a pre-heated oil bath at 50 °C. The same amount was added after 2 h stirring time, after 6 h stirring time, and after 22 h stirring time, respectively. After 24 h total stirring time, the mixture was cooled to room temperature, the solvent was evaporated under reduced pressure and CH_2Cl_2 (100 mL) was added. The organic phase was washed with $NaPF_6$ (aq., 5 g/L, 2×100 mL) and H_2O (100 mL). After evaporation of the solvent, the resulting solid was washed with heated toluene (50 °C). The solid was dissolved in CH_2Cl_2 (3 mL), precipitated in diethyl ether (30 mL) and washed with diethyl ether (50 mL in total). The solid was flushed from the frit with CH_2Cl_2 and the solvent was evaporated to yield the product (135 mg, quant.).

^{1}H-NMR (400 MHz, CD_3CN): δ = 8.14 (d, 4*H*, qu*H*, *J* = 5.12 Hz), 8.12 (s, 4*H*, py*H*), 8.09 (d, 4*H*, qu*H*, *J* = 8.12 Hz), 7.96 (d, 4*H*, Ph*H*, *J* = 8.12 Hz), 7.90 (d, 4*H*, qu*H*, *J* = 7.36 Hz), 7.72 (d, 4*H*, qu*H*, *J* = 7.68 Hz), 7.67 (d, 2*H*, Ph*H*, *J* = 8.48 Hz), 7.65 (d, 2*H*, Ph*H*, *J* = 8.48 Hz), 7.50 (m, 4*H*, qu*H*), 7.09 (dd, 4*H*, qu*H*, *J* = 8.00 Hz, *J* = 5.16 Hz), 1.16 (s, 21*H*, -Si(C*H*(C*H*$_3$)$_2$)$_3$), 0.27 (s, 9*H*, -Si(C*H*$_3$)) ppm.

^{13}C-APT-NMR (100 MHz, CD_3CN): δ = 159.47 (s, 4*C*, qu*C*H), 158.08 (s, 4*C*, qu*C*), 149.52 (s, 1*C*, Ph*C*), 149.43 (s, 1*C*, Ph*C*), 147.54 (s, 4*C*, qu*C*), 138.55 (s, 4*C*, qu*C*H), 137.07 (s, 1*C*, Ph*C*), 137.01 (s, 1*C*, Ph*C*), 134.44 (s, 4*C*, qu*C*H), 133.56 (s, 2*C*, Ph*C*H), 133.49 (s, 2*C*, Ph*C*H), 132.78 (s, 4*C*, py*C*), 131.61 (s, 4*C*,

qu*C*H), 128.66 (s, 2*C*, Ph*C*H), 128.64 (s, 2*C*, Ph*C*H), 127.82 (s, 4*C*, qu*C*H), 127.55 (s, 4*C*, qu*C*), 126.19 (s, 4*C*, py*C*H), 125.93 (s, 1*C*, py*C*), 125.73 (s, 1*C*, py*C*), 123.06 (s, 4*C*, qu*C*H), 107.23 (s, 1*C*, -*C*CSi), 104.98 (s, 1*C*, -*C*CSi), 97.73 (s, 1*C*, -C*C*Si), 94.25 (s, 1*C*, -*C*CSi), 18.94 (s, 6*C*, -Si(CH(CH_3)$_2$)$_3$), 12.03 (s, 3*C*, -Si(*C*H(CH_3)$_2$)$_3$), 0.23 (s, 3*C*, -Si(*C*H_3)$_3$) ppm.
HR-ESI-MS ([$C_{74}H_{66}N_6RuSi_2$]$^{2+}$): calc.: 598.1960; found: 598.1956; error: 2.2 ppm.

[Ru(dqpPhPhCH_2N_3)(dqpPhCCTIPS)]$^{2+}$.

[Ru(dqpPhBr)(dqpPhCCTIPS)]$^{2+}$ (11.20 mg, 7.62 µmol), 4-(azidomethyl)phenylboronic acid pinacol ester (9.84 mg, 37.98 µmol), RuPhos Pd G3 (4.72 mg, 5.65 µmol), RuPhos (4.07 mg, 8.72 µmol), and potassium phosphate (11.57 mg, 54.51 µmol) were combined in a vial. The vial was sealed with a rubber septum and the atmosphere replaced with nitrogen. THF (3 mL, after 3 freeze pump thaw-cycles) and H_2O (0.1 mL, after 3 freeze pump thaw-cycles) were added and the mixture placed in a pre-heated oil bath at 50 °C for 19 h. After cooling to room temperature, the mixture was put on KPF_6 (aq., 5 g/L, 50 mL) and extracted with CH_2Cl_2/MeOH (95:5, 2×25 mL, then 2×10 mL) and CH_2Cl_2 (2×10 mL). The combined organic phases were washed with H_2O (50 mL) and the aqueous layer extracted with CH_2Cl_2 (10 mL). The organic phases were combined and the solvent evaporated. The crude product was dissolved in CH_3CN (0.8 mL) and dropwised in diethyl ether (10 mL). The mixture was filtered and the precipitate washed with diethyl ether (5×10 mL). The precipitate was flushed from frit with CH_3CN, and the solvent was evaporated to yield the product (10 mg, 86%).
^{1}H-NMR (400 MHz, CD_2Cl_2): δ = 8.15-8.04 (m, 10*H*, qu*H*/py*H*), 8.02 (s, 2*H*, py*H*), 7.94 (d, 2*H*, Ph*H*, *J* = 8.42 Hz), 7.89-7.77 (m, 8*H*, qu*H*/Ph*H*), 7.75-7.64 (m, 8*H*, qu*H*/Ph*H*), 7.55 (dt, 4*H*, qu*H*, *J* = 7.77 Hz, *J* = 2.64 Hz), 7.45 (d, 2*H*, Ph*H*, *J* = 8.20 Hz), 7.21-7.13 (m, 4*H*, qu*H*), 4.42 (s, 2*H*, -C*H*$_2$N$_3$), 1.15 (s, 21*H*, -Si(C*H*(C*H*$_3$)$_2$)$_3$) ppm.
^{13}C-APT-NMR (100 MHz, CD_2Cl_2): δ = 158.33 (s, 2*C*, qu*C*H), 158.30 (s, 2*C*, qu*C*H), 157.28 (s, 2*C*, qu*C*), 157.18 (s, 2*C*, qu*C*), 150.54 (s, 1*C*, Ph*C*), 150.11 (s, 1*C*, Ph*C*), 146.99 (s, 2*C*, qu*C*), 146.98 (s, 2*C*, qu*C*), 143.02 (s, 1*C*, Ph*C*), 139.90 (s, 1*C*, Ph*C*), 138.36 (s, 4*C*, qu*C*H), 135.95 (s, 1*C*, py*C*/Ph*C*), 135.76 (s, 1*C*, py*C*/Ph*C*), 135.07 (s, 1*C*, py*C*/Ph*C*), 133.49 (s, 2*C*, Ph*C*H), 133.39 (s, 4*C*, qu*C*H), 132.19 (s, 2*C*, py*C*), 132.12 (s, 2*C*, py*C*), 131.45 (s, 2*C*, qu*C*H), 131.42 (s, 2*C*, qu*C*H), 129.27 (s, 2*C*, Ph*C*H), 128.46 (s, 2*C*, Ph*C*H), 128.21 (s, 2*C*, Ph*C*H), 127.83 (s, 2*C*, Ph*C*H), 127.63 (s, 4*C*, qu*C*H), 127.57 (s, 2*C*, Ph*C*H), 127.16 (s, 4*C*, qu*C*), 126.28 (s, 1*C*, Ph*C*), 125.96 (s, 2*C*, py*C*H), 125.93 (s, 2*C*, py*C*H), 122.77 (s, 4*C*, qu*C*H), 106.33 (s, 1*C*, -*C*CSi), 94.49 (s, 1*C*, -C*C*Si), 54.76 (s, 1*C*, -C*H*$_2$N$_3$),18.79 (s, 6*C*, -Si(CH(*C*H_3)$_2$)$_3$), 11.66 (s, 3*C*, -Si(*C*H(CH_3)$_2$)$_3$) ppm.
HR-ESI-MS ([C76H63N9RuSi]2+): calc.: 615.7004; found: 615.6996; error: 2.9 ppm.

[Ru(dqpPhB(pin))(dqpPhCCTIPS)]$^{2+}$.

Synthesis according to adopted literature procedure.[9]
[Ru(dqpPhBr)(dqpPhCCTIPS)]$^{2+}$ (0.198 g, 0.135 mmol), bis(pinakolato)diboron (0.071 g, 0.278 mmol), Pd(dppf)Cl_2 (0.012 g, 0.016 mmol), and potassium acetate (0.043 g, 0.442 mmol) were combined in a microwave vial which was sealed and put under reduced pressure for 2 h. Subsequent, the atmosphere inside was replaced with nitrogen and dry DMF (10 mL) was added. The vial was purged with a nitrogen flow for 10 min and placed in a pre-heated oil bath at 80 °C overnight. After cooling to room temperature, the mixture was put on $NaPF_6$ (aq., 5 g/L, 100 mL) and extracted with CH_2Cl_2 (100 mL, then 20 mL). The combined organic phases were washed with H_2O (120 mL) and the solvent was evaporated under reduced pressure. The crude product was dissolved in CH_2Cl_2 (10 mL) and dropwised in cooled diethyl ether (100 mL). The precipitate was filtered, washed with diethyl ether (100 mL, then 3×50 mL), and flushed from the frit with CH_2Cl_2. The solvent was evaporated to yield the product (0.200 g, 98%).

^{1}H-NMR (400 MHz, CD_2Cl_2): δ = 8.14-8.06 (m, 8*H*, qu*H*), 8.03 (d, 4*H*, py*H*), 7.97 (d, 2*H*, Ph*H*, *J* = 8.04 Hz), 7.82 (m, 8*H*, qu*H*+Ph*H*), 7.70 (d, 4*H*, qu*H*, *J* = 8.16 Hz), 7.67 (d, 2*H*, Ph*H*, *J* = 8.38 Hz), 7.54 (dt, 4*H*, qu*H*, *J* = 7.67 Hz, Hz, *J* = 1.53 Hz), 7.16 (m, 4*H*, qu*H*), 1.36 (s, 12*H*, -C*H*$_3$), 1.15 (s, 21*H*, -SiC*H*(C*H*$_3$)$_2$) ppm.
^{13}C-APT-NMR (100 MHz, CD_2Cl_2): δ = 158.35 (s, 2*C*, qu*C*H), 158.24 (s, 2*C*, qu*C*H), 157.25 (s, 4*C*, qu*C*), 150.84 (s, 1*C*, Ph*C*), 150.16 (s, 1*C*, Ph*C*), 146.97 (s, 2*C*, qu*C*), 146.95 (s, 2*C*, qu*C*), 138.38 (s, 2*C*, qu*C*H), 138.36 (s, 2*C*, qu*C*H), 136.11 (s, 2*C*, Ph*C*H), 135.75 (s, 2*C*, py*C*), 133.53 (s, 2*C*, Ph*C*H), 133.45 (s, 2*C*, Ph*C*H), 133.38 (s, 4*C*, qu*C*H), 132.12 (s, 4*C*, py*C*), 131.45 (s, 4*C*, qu*C*H), 127.66 (s, 2*C*, Ph*C*H), 127.58 (s, 4*C*, qu*C*H), 127.16 (s, 2*C*, qu*C*), 127.14 (s, 2*C*, qu*C*), 126.82 (s, 4*C*, qu*C*H), 126.27 (s, 1*C*, Ph*C*), 126.14 (s, 2*C*, py*C*H), 125.95 (s, 2*C*, py*C*H), 122.82 (s, 2*C*, qu*C*H), 122.72 (s, 2*C*, qu*C*H), 106.33 (s, -SiC*C*-), 94.46 (s, 1*C*, -Si*C*C-), 84.62 (s, 2*C*, -BO*C*-), 25.07 (s, 2*C*, -*C*H$_3$), 25.05 (s, 2*C*, -*C*H$_3$), 18.79 (s, 6*C*, -*C*H$_3$), 11.66 (s, 3*C*, -*C*H-) ppm.
HR-ESI ([$C_{75}H_{69}BN_6O_2RuSi$]$^{2+}$): calc.: 613.2203 m/z, found: 613.2203 m/z, error: 0.0 ppm.

[Ru(dqpPhPhNDI)(dqpPhCCTIPS)]$^{2+}$.
NDIPhI (18.76 mg, 32.32 µmol), RuPhos Pd G3 (16.22 mg, 19.39 µmol), RuPhos (13.66 mg, 29.27 µmol), and potassium phosphate (13.59 mg, 64.02 µmol) were combined in a vial which was sealed with a rubber septum. The atmosphere was replaced by nitrogen and THF (1 mL, after 3 freeze pump thaw-cycles) and H_2O (0.1 mL, after 3 freeze pump thaw-cycles) were added. The vial was placed in a pre-heated oil bath at 50 °C. After 1 h, **[Ru(dqpPhB(pin))(dqpPhCCTIPS)]$^{2+}$** (9.62 mg, 6.34 µmol) under a nitrogen atmosphere and dissolved in THF (2 mL, after 3 freeze pump thaw-cycles) was added and the resulting mixture stirred for 17 h in the 50 °C oil bath. The mixture was cooled to room temperature and put on KPF_6 (aq., 5 g/L, 100 mL). The aqueous layer was extracted with CH_2Cl_2/MeOH (90:10, 100 mL, then 95:5, 20 mL). The combined organic phases were washed with H_2O (100 mL) and the solvent was evaporated under reduced pressure. The crude product was dissolved in CH_3CN (0.8 mL) and dropwised in diethyl ether (10 mL). The precipitate was filtered off and washed with diethyl ether (3×10 mL). The precipitate was flushed from frit with CH_3CN, and the solvent was evaporated to yield the product (10 mg, 86%).
^{1}H-NMR (400 MHz, CD_2Cl_2): δ = 8.80 (s, 4*H*, NDI*H*), 8.16-8.05 (m, 10*H*, qu*H*/py*H*), 8.03 (s, 2*H*, py*H*), 7.98 (d, 2*H*, Ph*H*, *J* = 8.40 Hz), 7.92 (d, 2*H*, Ph*H*, *J* = 8.40 Hz), 7.89 (d, 2*H*, Ph*H*, *J* = 8.40 Hz), 7.87-7.78 (m, 6*H*, qu*H*/Ph*H*), 7.72 (d, 4*H*, qu*H*, *J* = 8.22 Hz), 7.68 (d, 2*H*, Ph*H*, *J* = 8.33 Hz), 7.56 (dt, 4*H*, qu*H*, *J* = 7.76 Hz, *J* = 3.04 Hz), 7.47 (d, 2*H*, Ph*H*, *J* = 8.38 Hz), 7.19 (m, 4*H*, qu*H*), 4.15 (m, 2*H*, -NC*H*$_2$-), 1.95 (m, 1*H*, -C*H*-), 1.47-1.28 (m, 8*H*, -C*H*$_2$-), 1.16 (s, 21*H*, -Si(C*H*(C*H*$_3$)$_2$)$_3$), 0.95 (t, 3*H*, -C*H*$_3$, *J* = 7.42 Hz), 0.90 (t, 3*H*, -C*H*$_3$, *J* = 7.03 Hz) ppm.
^{13}C-APT-NMR (100 MHz, CD_2Cl_2): δ = 163.55 (s, 4*C*, -*C*(O)-), 158.39 (s, 4*C*, qu*C*H), 157.33 (s, 4*C*, qu*C*), 150.61 (s, 1*C*, Ph*C*), 150.21 (s, 1*C*, Ph*C*), 147.05 (s, 4*C*, qu*C*), 142.76 (s, 1*C*, Ph*C*), 140.85 (s, 1*C*, Ph*C*), 138.41 (s, 4*C*, qu*C*H), 135.79 (s, 1*C*, Ph*C*), 135.45 (s, 4*C*, pyC), 133.53 (s, 4*C*, qu*C*H), 133.42 (s, 2*C*, Ph*C*H),132.20 (s, 4*C*, qu*C*), 131.60 (s, x*C*, qu*C*H/NDI*C*H), 131.49 (s, x*C*, qu*C*H/NDI*C*H), 131.32 (s, x*C*, qu*C*H/NDI*C*H), 129.70 (s, 2*C*, Ph*C*H), 128.80 (s, 2*C*, Ph*H*), 128.54 (s, 2*C*, Ph*H*), 128.33 (s, 2*C*, Ph*H*), 127.68 (s, 4*C*, qu*C*H), 127.62 (s, 2*C*, Ph*H*), 127.23 (s, 4*C*, NDI*C*), 127.00 (s, 2*C*, py*C*), 126.36 (s, 1*C*, Ph*C*), 126.08 (s, 2*C*, py*C*H), 125.99 (s, 2*C*, py*C*H), 122.83 (s, 4*C*, qu*C*H), 106.37 (s, 1*C*, -*C*CSi), 94.58 (s, 1*C*, -C*C*Si), 44.90 (s, 1*C*, -N*C*H$_2$-), 38.40 (s, 1*C*, -*C*H-), 31.09 (s, 1*C*, -*C*H$_2$-), 29.05 (s, 1*C*, -*C*H$_2$-), 24.41 (s, 1*C*, -*C*H$_2$-), 23.46 (s, 1*C*, -*C*H$_2$-), 18.82 (s, 6*C*, -Si(CH(*C*H$_3$)$_2$)$_3$), 14.25 (s, 1*C*, -*C*H$_3$), 11.72 (s, 3*C*, -Si(*C*H(CH$_3$)$_2$)$_3$), 10.77 (s, 1*C*, -*C*H$_3$) ppm.
HR-ESI-MS ([[$C_{97}H_{82}N_8O_4RuSi$]$^{2+}$): calc. 776.2630; found: 776.2631; error: 1.7 ppm.

[Ru(dqpPhPhTARA)(dqpPhCCTIPS)]$^{2+}$.
TARABr (24.07 mg, 68.32 µmol), RuPhos Pd G3 (8.22 mg, 9.84 µmol), RuPhos (7.35 mg, 15.75 µmol), and potassium phosphate (27.70 mg, 130.50 µmol) were combined in a vial which was sealed with a

rubber septum. The atmosphere was replaced by nitrogen and THF (2 mL, after 3 freeze pump thaw-cycles) and H_2O (0.25 mL, after 3 freeze pump thaw-cycles) were added. The vial was placed in a pre-heated oil bath at 50 °C. After 1 h, the mixture was added to a solution of **[Ru(dqpPhB(pin))(dqpPhCCTIPS)]$^{2+}$** (20.59 mg, 13.58 µmol) in THF (3 mL, after 3 freeze pump thaw-cycles) under a nitrogen atmosphere.The resulting mixture was stirred for 24 h in the 50 °C oil bath. The mixture was cooled to room temperature and put on KPF_6 (aq., 5 g/L, 100 mL). The aqueous layer was extracted with CH_2Cl_2 (100 mL, then 3×20 mL). The combined organic phases were washed with H_2O (100 mL), the aqueous layer was extracted with CH_2Cl_2 (2×20 mL) and the solvent of the combined organic layers was evaporated under reduced pressure. The crude product was dissolved in CH_3CN (2 mL) and dropwised in diethyl ether (20 mL). diethyl ether (20 mL) was added, the precipitate was filtered off and washed with diethyl ether (5×20 mL). The precipitate was flushed from frit with CH_3CN, and the solvent was evaporated. The crude product was subjected to flash column chromatography (SiOH, CH_2Cl_2/MeOH 98:2 to 90:10) to yield the product (7.9 mg, 35%).

^{1}H-NMR (600 MHz, CD_2Cl_2): δ = 8.12-8.06 (m, 10*H*, qu*H*+py*H*), 8.02 (s, 2*H*, py*H*), 7.89 (d, 2*H*, Ph*H*, *J* = 8.40 Hz), 7.85-7.77 (m, 8*H*, qu*H*+Ph*H*), 7.71 (d, 4*H*, qu*H*, *J* = 8.22 Hz), 7.68 (d, 2*H*, Ph*H*, *J* = 8.16 Hz), 7.57-7.52 (m, 6*H*, qu*H*+TARA*H*), 7.20-7.14 (m, 4*H*, qu*H*), 7.11 (d, 4*H*, TARA*H*, *J* = 7.98 Hz), 7.06 (d, 2*H*, TARA*H*, *J* = 8.34 Hz), 7.02 (d, 4*H*, TARA*H*, *J* = 7.92 Hz), 2.32 (s, 6*H*, -C*H*$_3$), 1.15 (m, 21*H*, -C*H*$_3$) ppm.

^{13}C-APT-NMR (150 MHz, CD_2Cl_2): δ = 158.33 (s, 2*C*, qu*C*H), 158.30 (s, 2*C*, qu*C*H), 157.28 (s, 2*C*, qu*C*), 157.10 (s, 2*C*, qu*C*), 150.66 (s, 1*C*, Ph*C*), 150.14 (s, 1*C*, Ph*C*), 147.00 (s, 4*C*, py*C*), 145.22 (s, 2*C*, TARA*C*), 143.39 (s, 1*C*, Ph*C*), 138.37 (s, 2*C*, qu*C*H), 138.35 (s, 2*C*, qu*C*H), 135.74 (s, 2*C*, py*C*), 133.82 (s, 1*C*, TARA*C*), 133.72 (s, 2*C*, TARA*C*), 133.50 (s, 2*C*, Ph*C*H), 133.40 (s, 4*C*, qu*C*H),132.24 (s, 2*C*, qu*C*), 132.15 (s, 2*C*, qu*C*), 132.02 (s, 1*C*, TARA), 131.45 (s, 2*C*, qu*C*H), 131.41 (s, 2*C*, qu*C*H), 130.35 (s, 4*C*, TARA*C*H), 128.03 (s, 2*C*, Ph*C*H), 127.89 (s, 2*C*, TARA*C*H), 127.64 (s, 2*C*, Ph*C*H), 127.63 (s, 2*C*, Ph*C*H), 127.56 (s, 4*C*, qu*C*H), 127.17 (s, 4*C*, qu*C*), 126.31 (s, 1*C*, Ph*C*), 125.95 (s, 2*C*, py*C*H), 125.78 (s, 2*C*, py*C*H), 125.42 (s, 4*C*, TARA*C*H), 122.78 (s, 2*C*, qu*C*H), 122.73 (s, 2*C*, qu*C*H), 122.22 (s, 2*C*, TARA*C*H), 106.31 (s, 1*C*, -*C*CSi), 94.54 (s, 1*C*, -C*C*Si) 20.93 (s, 2*C*, -*C*H$_3$), 18.80 (s, 6*C*, -*C*H$_3$), 11.67 (s, 3*C*, -Si*C*H-) ppm.

HR-ESI-MS ([$C_{89}H_{75}N_7RuSi$]$^{2+}$): calc.: 685.7455 m/z, found: 685.7444 m/z, error: 1.6 ppm.

[Ru(dqpPhTARA)(dqpPhCCNDI)]$^{2+}$.

[Ru(dqpPhPhTARA)(dqpPhCCTIPS)]$^{2+}$ (5.90 mg, 3.55 µmol) was placed in a vial which was sealed with a rubber septum. The atmosphere was replaced with nitrogen and dry THF (2 mL) was added. The mixture was cooled in a ice/water bath under stirring. Tetrabutylammonium fluoride (0.05 M in THF, 0.35 mL, 17.50 µmol) was added and the resulting mixture stirred for 1 h in the cooling bath. The mixture was subsequently put on NH_4PF_6 (aq., 5 g/L, 50 mL) and the auqeous phase extracted with CH_2Cl_2 (50 mL, then 3 × 25 mL). The combined organic phases were combined, washed with H_2O (125 mL) and the solvent was evaporated. The crude product was directly used for the following step.

Crude **[Ru(dqpPhPhTARA)(dqpPhCCH)]$^{2+}$** (5.34 mg, 3.55 µmol), **NDIPhI** (11.22 mg, 19.33 µmol), copper(I) iodide (0.51 mg, 2.68 µmol), RuPhos Pd G3 (1.47 mg, 1.76 µmol), and RuPhos (1.02 mg, 2.19 µmol) were combined in a vial. The vial was sealed with a rubber septum, the atmosphere replaced with nitrogen and dry THF (3 mL) and dry triethylamine (1 mL) were added. The mixture was placed in a pre-heated oil bath at 50 °C for 40 h under stirring. After cooling to room temperature, the mixture was put on EDTA disodium salt solution (100 mL, aq., 0.05 M) and the aqueous layer extracted with CH2Cl2 (100 mL, then 20 mL). The combined organic phases were washed with KPF_6 (aq., 5 g/L, 100 mL) and the aqueous layer extracted with CH_2Cl_2 (20 mL). The combined organic phases were washed with H_2O (100 mL) and the aqueous layer extracted with CH_2Cl_2 (20 mL). The organic phases were combined and the solvent evaporated under reduced pressure. The crude product was subjected to flash column chromatography (40 g SiOH, CH_2Cl_2/MeOH 98:2 to 90:10). Product containing fractions were combined and the solvent evaporated under reduced pressure. The resulting solid was washed

with EtOH (5×2 mL), then diethyl ether (3×2 mL), again EtOH (3×5 mL) and diethyl ether (3×5 mL) and filtered after each washing step. The solid was separately flushed through the filter with CH_2Cl_2 and the solvent was evaporated to yield the product (5.4 mg, 78%).

^{1}H-NMR (600 MHz, CD_2Cl_2): δ = 8.79 (s, 4*H*, NDI*H*), 8.13-8.05 (m, 12*H*, qu*H*+py*H*+Ph*H*), 7.91-7.86 (m, 4*H*, qu*H*), 7.86-7.82 (m, 4*H*, qu*H*), 7.81 (d, 2*H*, Ph*H*, *J* = 2.96 Hz), 7.80 (d, 2*H*, Ph*H*, *J* = 3.05 Hz), 7.78 (d, 2*H*, Ph*H*, *J* = 8.33 Hz), 7.72 (d, 4*H*, qu*H*, *J* = 8.27 Hz), 7.59-7.52 (m, 6*H*, qu*H*+TARA*H*), 7.38 (d, 2*H*, Ph*H*, *J* = 8.33 Hz), 7.21-7.16 (m, 4*H*, qu*H*), 7.12 (d, 4*H*, TARA*H*, *J* = 8.25 Hz), 7.06 (d, 2*H*, TARA*H*, *J* = 8.57 Hz), 7.02 (d, 4*H*, TARA*H*, *J* = 8.33 Hz), 4.20-4.10 (m, 2*H*, -NCH_2-), 2.33 (s, 6*H*, -CH_3), 1.94 (m, 1*H*, -C*H*-), 1.44-1.28 (m, 8*H*, -CH_2-), 0.95 (t, 3*H*, -CH_3, *J* = 7.43 Hz), 0.89 (m, 3*H*, -CH_3) ppm.

^{13}C-APT-NMR (150 MHz, CD_2Cl_2): δ=163.53 (s, 2*C*, -*C*(O)-)), 163.40 (s, 2*C*, -*C*(O)-), 158.39 (s, 2*C*, qu*C*H), 158.35 (s, 2*C*, qu*C*H), 157.35 (s, 2*C*, qu*C*), 157.13 (s, 2*C*, qu*C*), 150.75 (s, $C_{qu.}$), 149.06 (s, 1*C*, TARA*C*), 147.06 (s, 4*C*, qu*C*), 145.25 (s, $C_{qu.}$), 143.50 (s, $C_{qu.}$), 138.39 (s, 4*C*, Ph*C*H), 133.79 (s, $C_{qu.}$), 133.54 (s, 2*C*, qu*C*H), 133.44 (s, 2*C*, qu*C*H), 133.22 (s, 4*C*, qu*C*), 133.01 (s, 2*C*, Ph*C*H), 132.30 (s, $C_{qu.}$), 132.21 (s, $C_{qu.}$), 131.64 (s, $C_{qu.}$), 131.51 (s, 2*C*, qu*C*H), 131.46 (s, 2*C*, qu*C*H), 131.31 (s, 4*C*, NDI*C*H), 130.39 (s, 4*C*, TARA*C*H), 129.45 (s, 2*C*, Ph*C*H), 128.03 (s, $C_{qu.}$), 127.91 (s, 4*C*, qu*C*H), 127.78 (s, 2*C*, TARA*C*H), 127.70 (s, 4*C*, qu*C*H), 127.66 (s, 2*C*, Ph*C*H), 127.63 (s, 2*C*, Ph*C*H), 127.24 (s, $C_{qu.}$), 126.90 (s, $C_{qu.}$), 126.04 (s, 2*C*, py*C*H), 125.83 (s, 2*C*, py*C*H), 125.48 (s, 4*C*, TARA*C*H), 122.82 (s, 2*C*, qu*C*H), 122.79 (s, 2*C*, qu*C*H), 122.22 (s, 2*C*, TARA*C*H), 44.90 (s, 1*C*, -N*C*H_2-), 38.39 (s, 1*C*, -*C*H-), 31.08 (s, 1*C*, -*C*H_2-), 29.04 (s, 1*C*, -*C*H_2-), 23.46 (s, 1*C*, -*C*H_2-), 23.10 (s, 1*C*, -*C*H_2-), 20.95 (s, 2*C*, -*C*H_3), 14.26 (s, 1*C*, -*C*H_3), 10.76 (s, 1*C*, -*C*H_3) ppm.

HR-ESI-MS ($[C_{108}H_{79}N_9O_4Ru]^{2+}$): calc.: 833.7644 m/z, found: 833.7625 m/z, error: 4.0 ppm.

NMR data

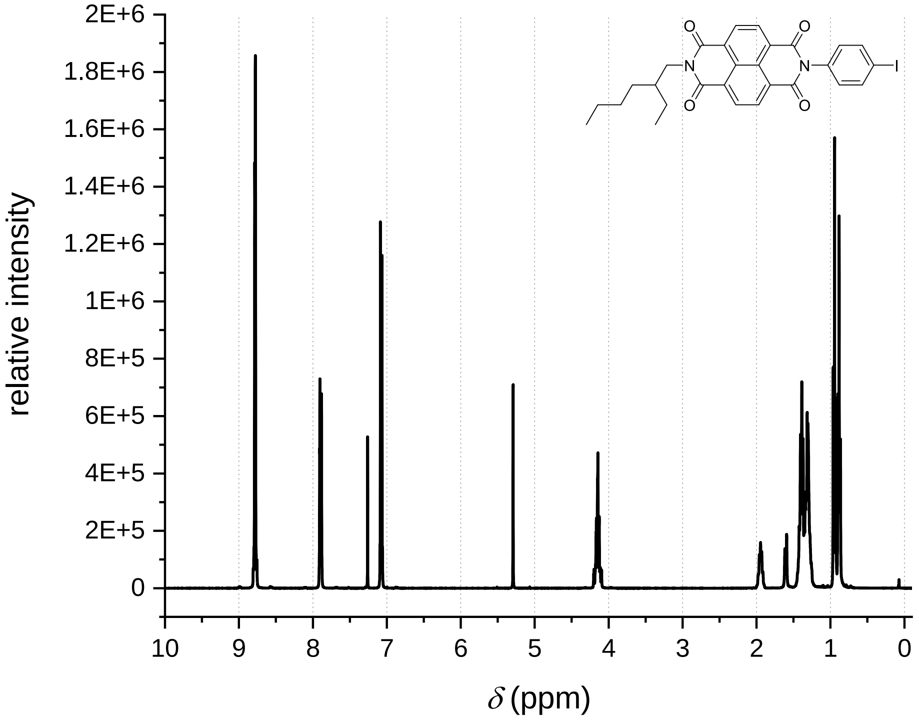

Figure S1. **^{1}H-NMR spectrum of NDIPhI (400 MHz, $CDCl_3$).**

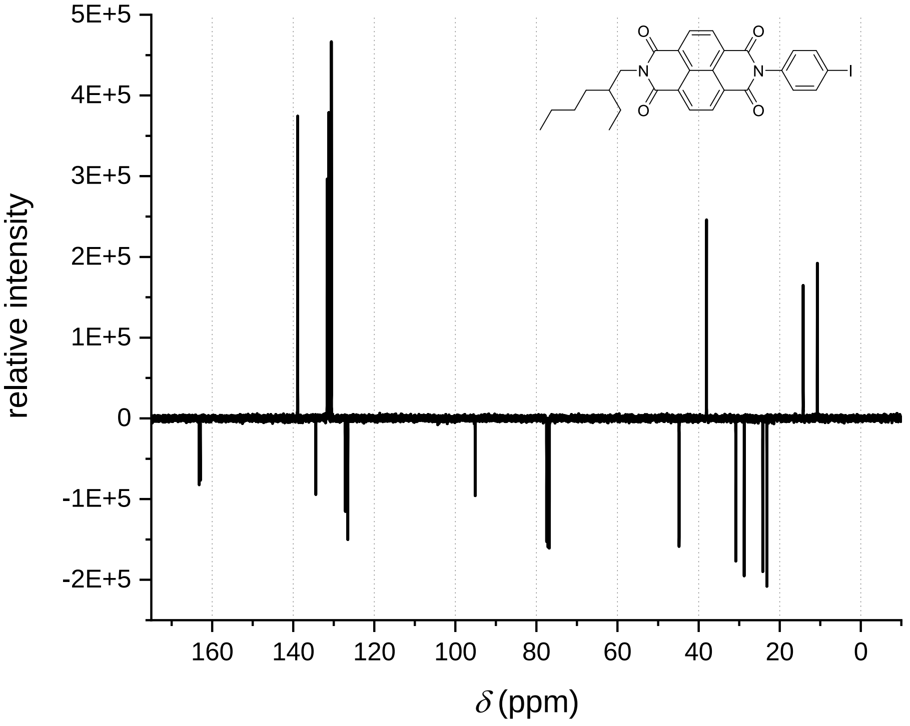

Figure S2. **$^{13}C\{^{1}H\}$-APT-NMR spectrum of NDIPhI (100 MHz, $CDCl_3$).**

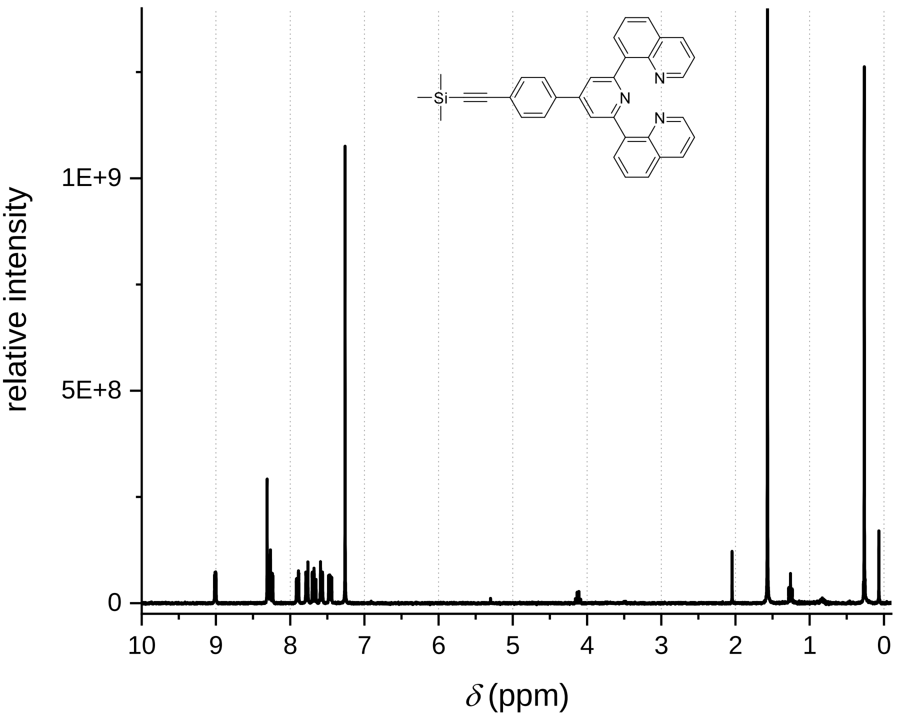

Figure S3. **^{1}H-NMR spectrum of dqpPhCCTMS (300 MHz, $CDCl_3$).**

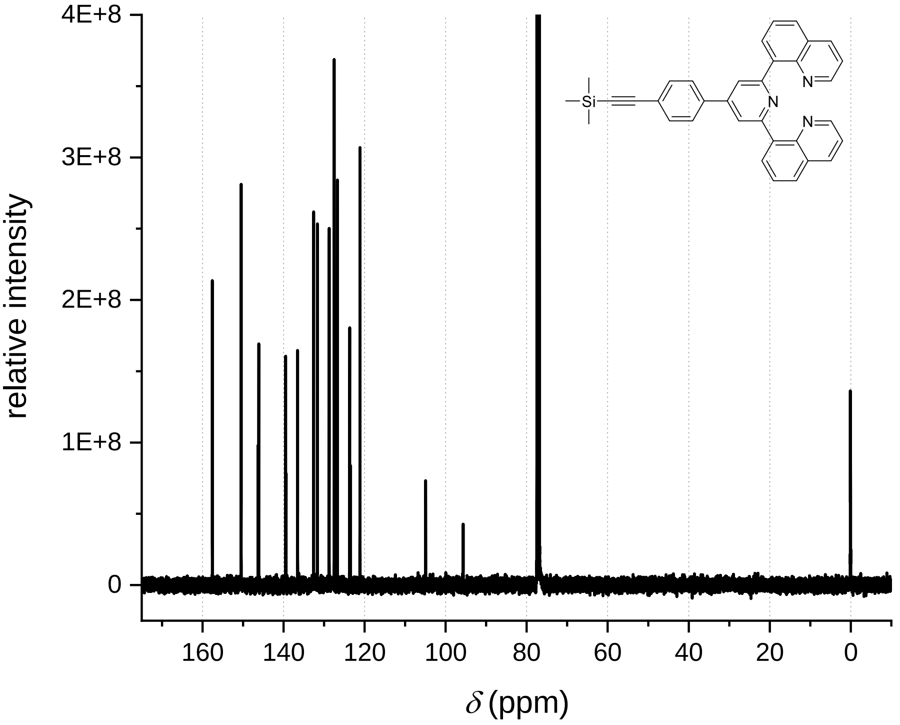

Figure S4. **^{13}C{^{1}H}-NMR spectrum of dqpPhCCTMS (100 MHz, $CDCl_3$).**

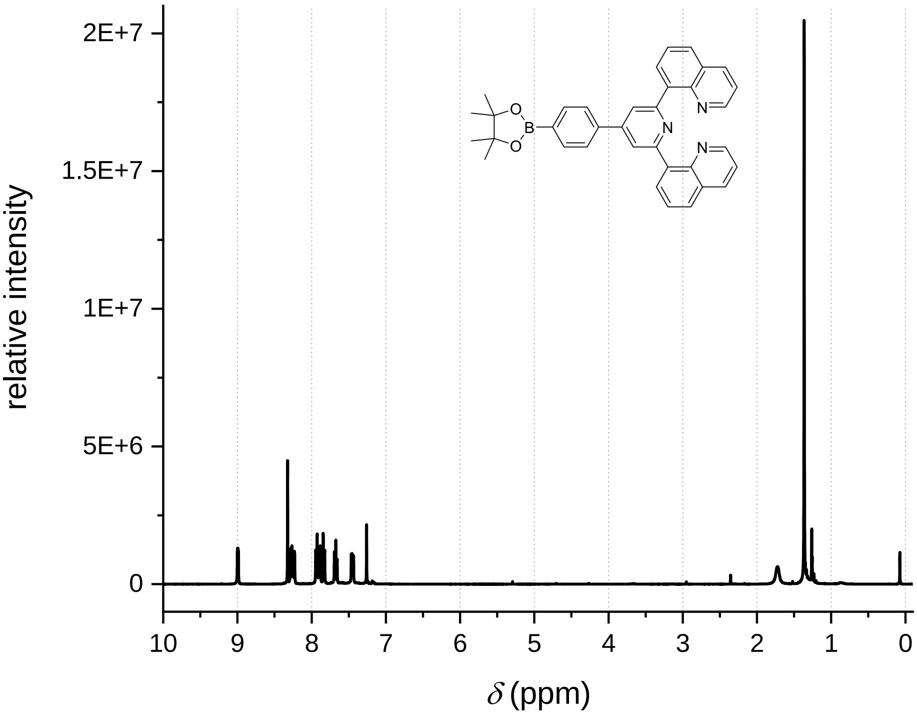

Figure S5. **^{1}H-NMR spectrum of dqpPhB(pin) (400 MHz, $CDCl_3$).**

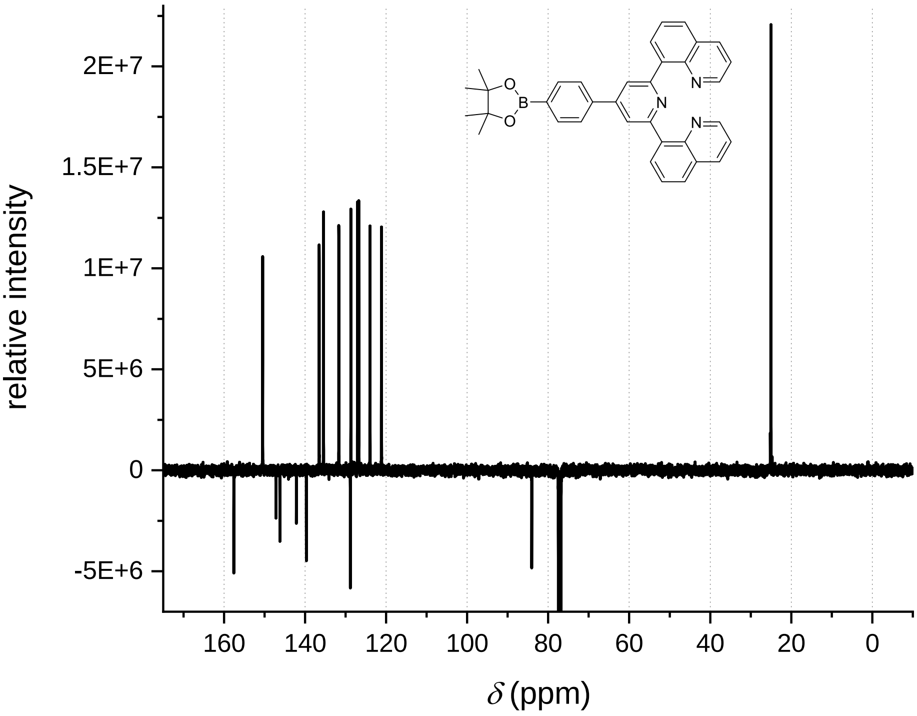

Figure S6. **$^{13}C\{^{1}H\}$-APT-NMR spectrum of dqpPhB(pin) (100 MHz, $CDCl_3$).**

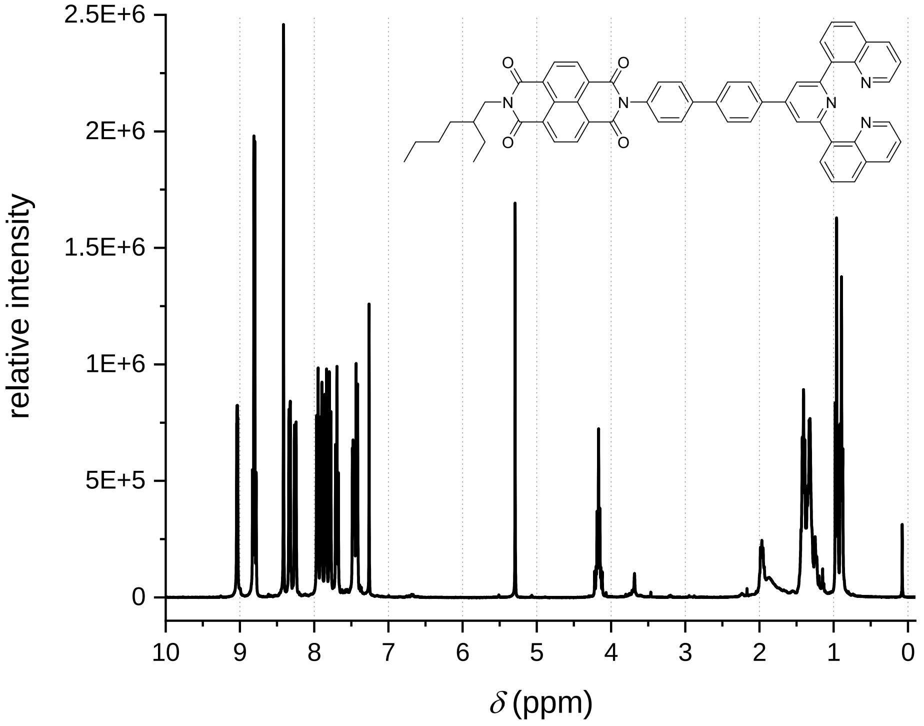

Figure S7. **^{1}H-NMR spectrum of dqpPhPhNDI (400 MHz, $CDCl_3$).**

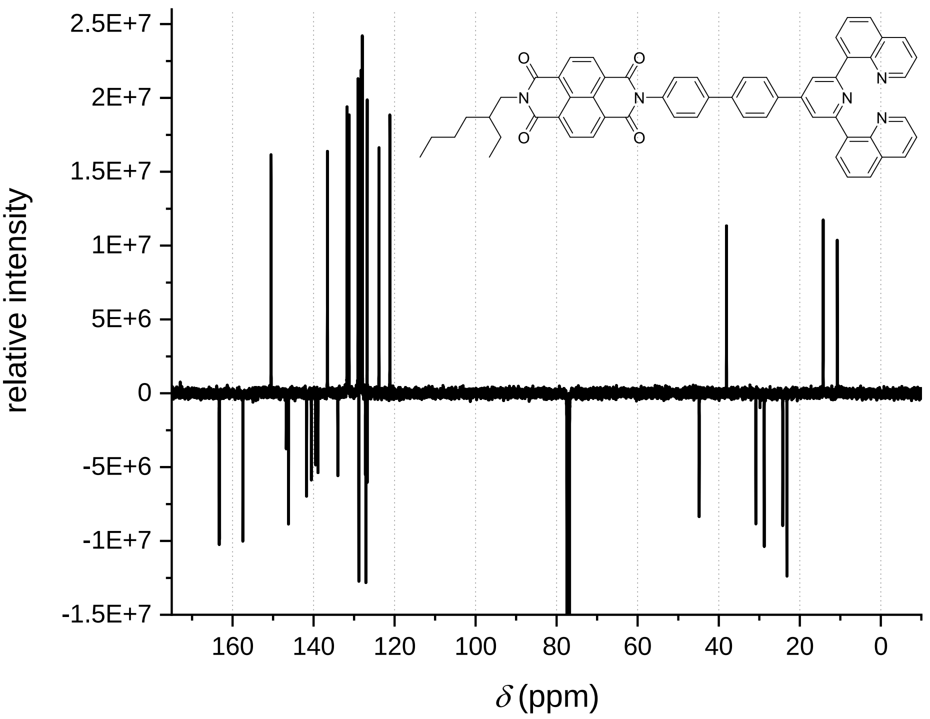

Figure S8. **$^{13}C\{^1H\}$-APT-NMR spectrum of dqpPhPhNDI (100 MHz, $CDCl_3$).**

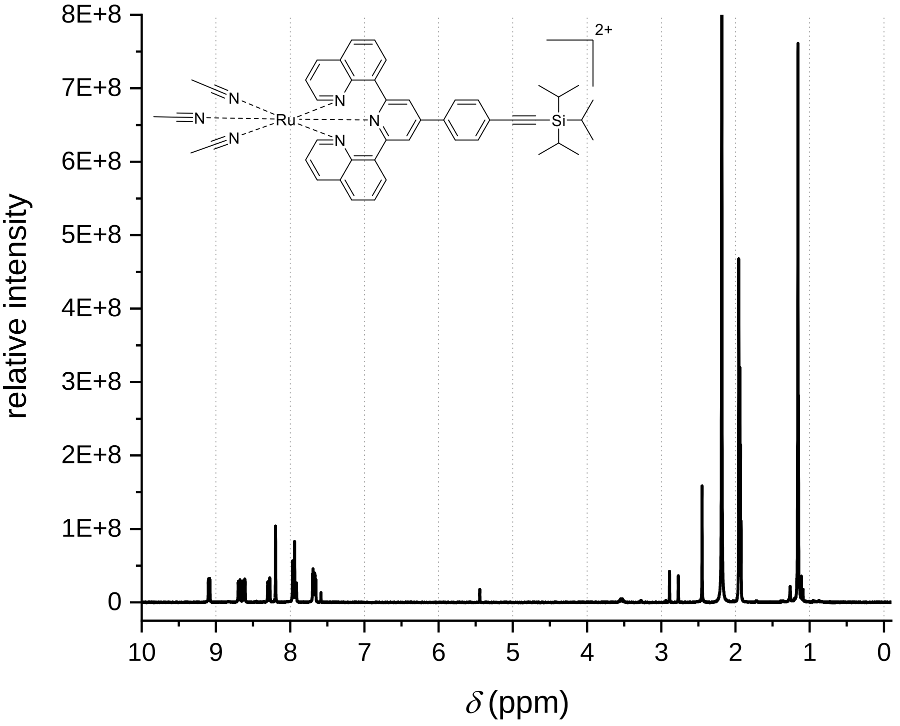

Figure S9. **^{1}H-NMR spectrum of $[Ru(dqpPhCCTIPS)(CH_3CN)_3]^{2+}$ (300 MHz, CD_3CN).**

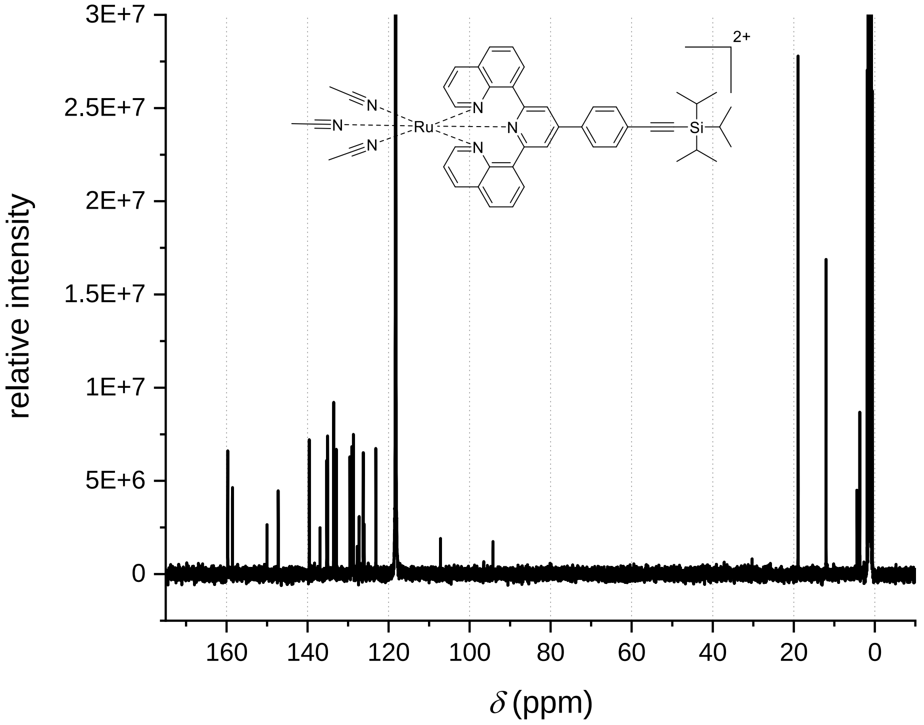

Figure S10. **$^{13}C\{^{1}H\}$-NMR spectrum of $[Ru(dqpPhCCTIPS)(CH_3CN)_3]^{2+}$ (100 MHz, CD_3CN).**

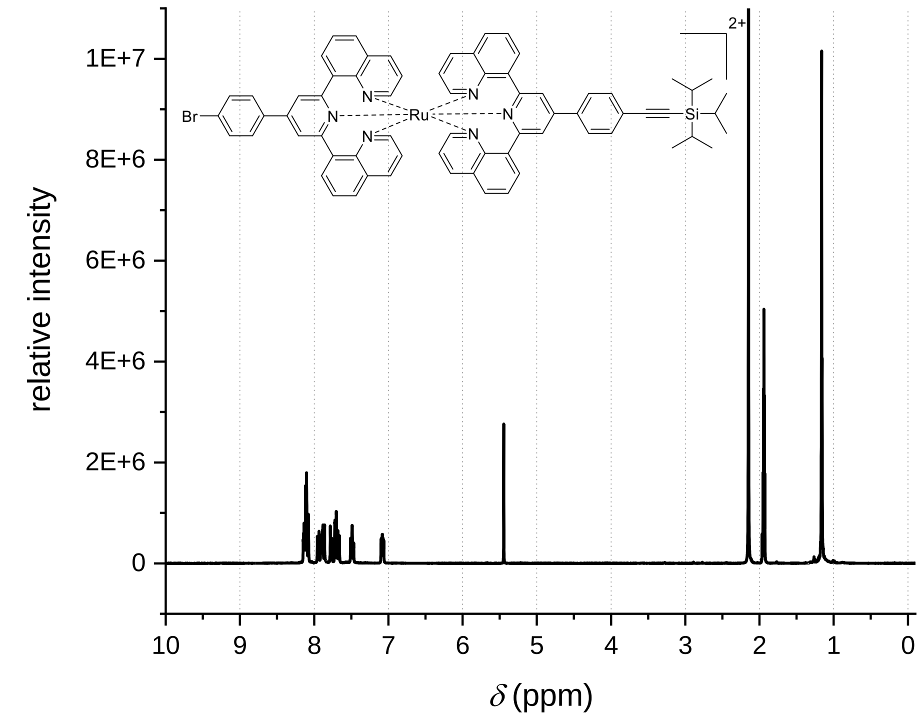

Figure S11. ^{1}H-NMR spectrum of $[Ru(dqpPhBr)(dqpPhCCTIPS)]^{2+}$ (400 MHz, CD_3CN).

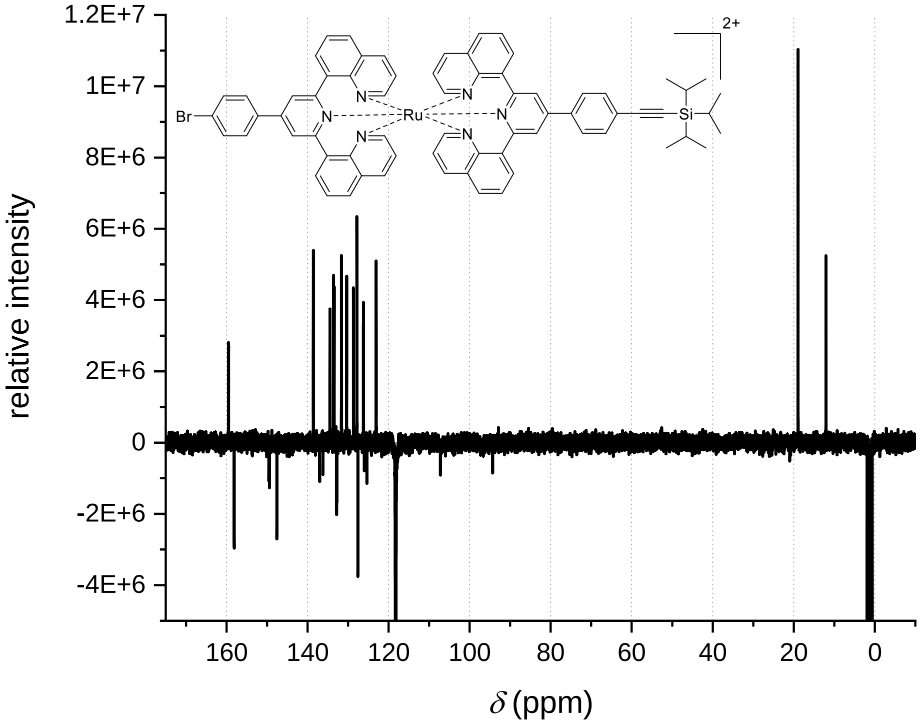

Figure S12. $^{13}C\{^{1}H\}$-APT-NMR spectrum of $[Ru(dqpPhBr)(dqpPhCCTIPS)]^{2+}$ (100 MHz, CD_3CN).

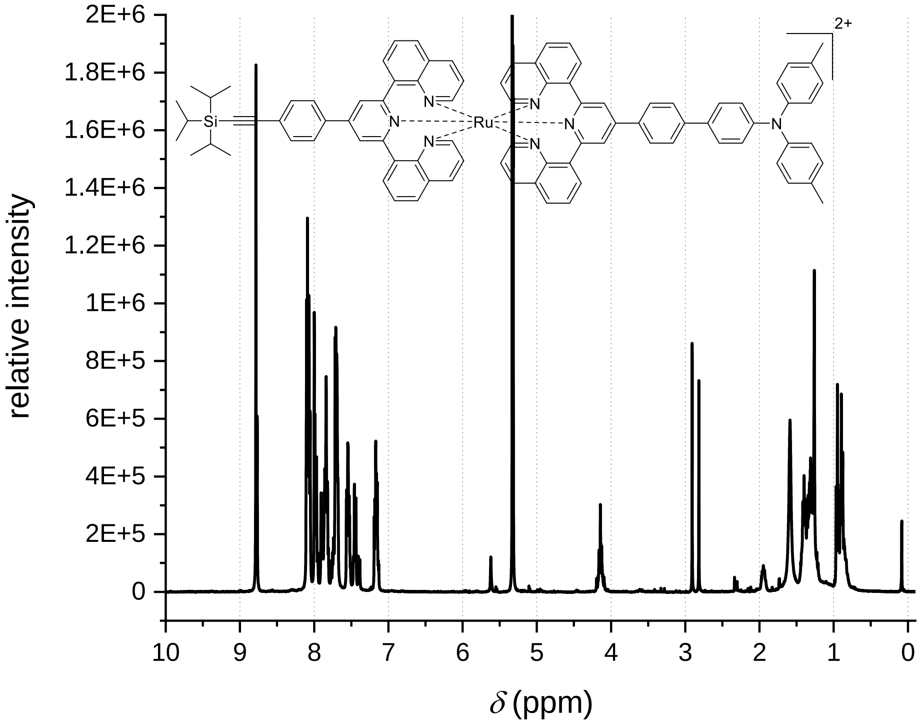

Figure S13. ^{1}H-NMR spectrum of $[Ru(dqpPhPhNDI)(dqpPhBr)]^{2+}$ (400 MHz, CD_2Cl_2).

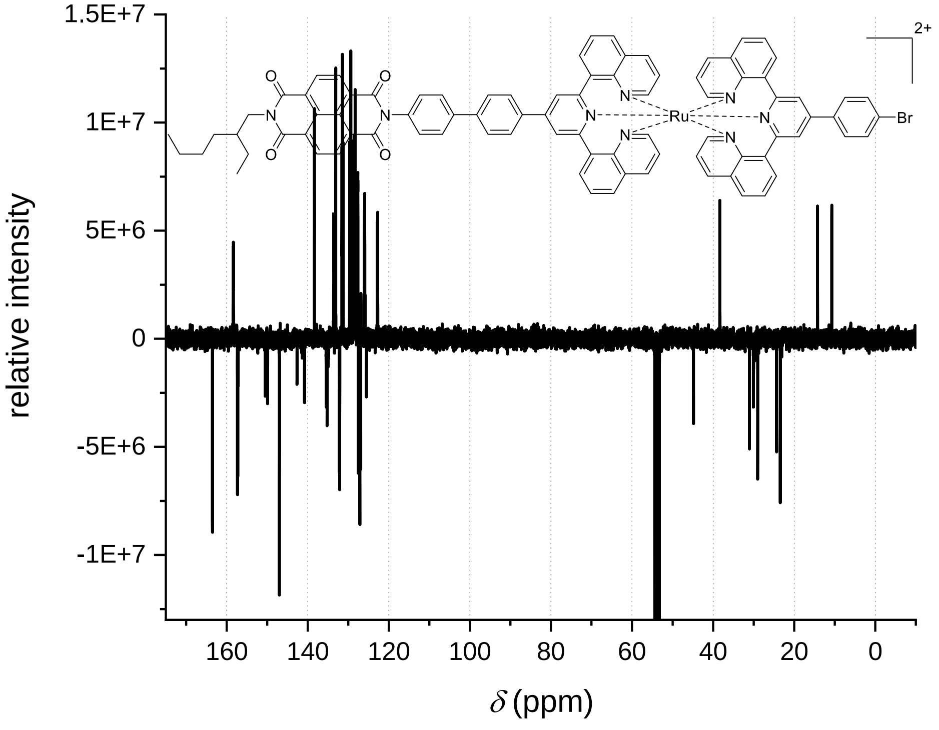

Figure S14. $^{13}C\{^{1}H\}$-APT-NMR spectrum of $[Ru(dqpPhPhNDI)(dqpPhBr)]^{2+}$ (100 MHz, CD_2Cl_2).

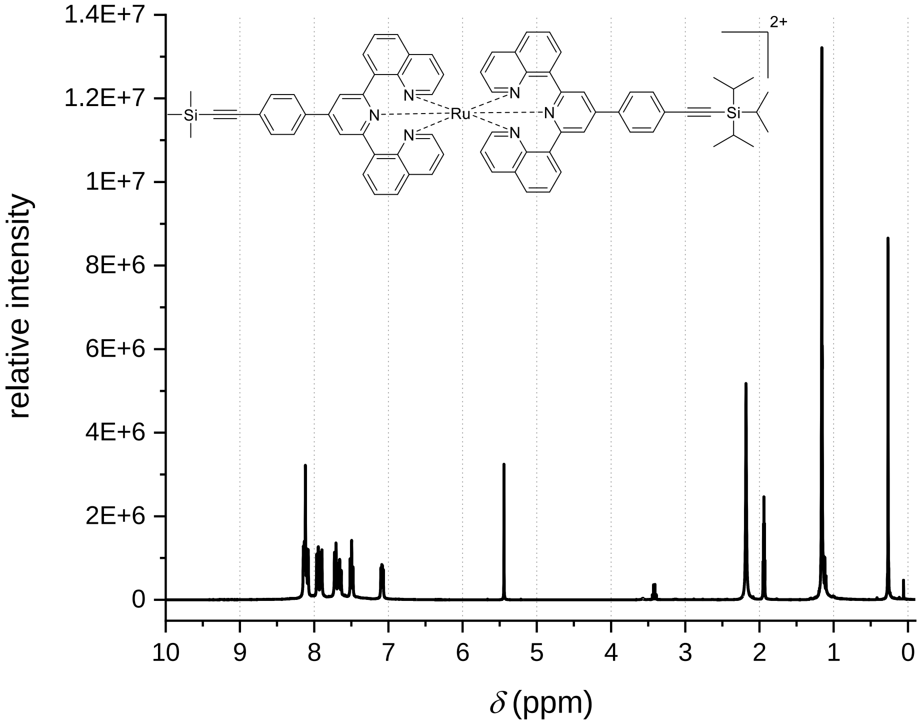

Figure S15. **^{1}H-NMR spectrum of [Ru(dqpPhCCTMS)(dqpPhCCTIPS)]$^{2+}$ (400 MHz, CD_3CN).**

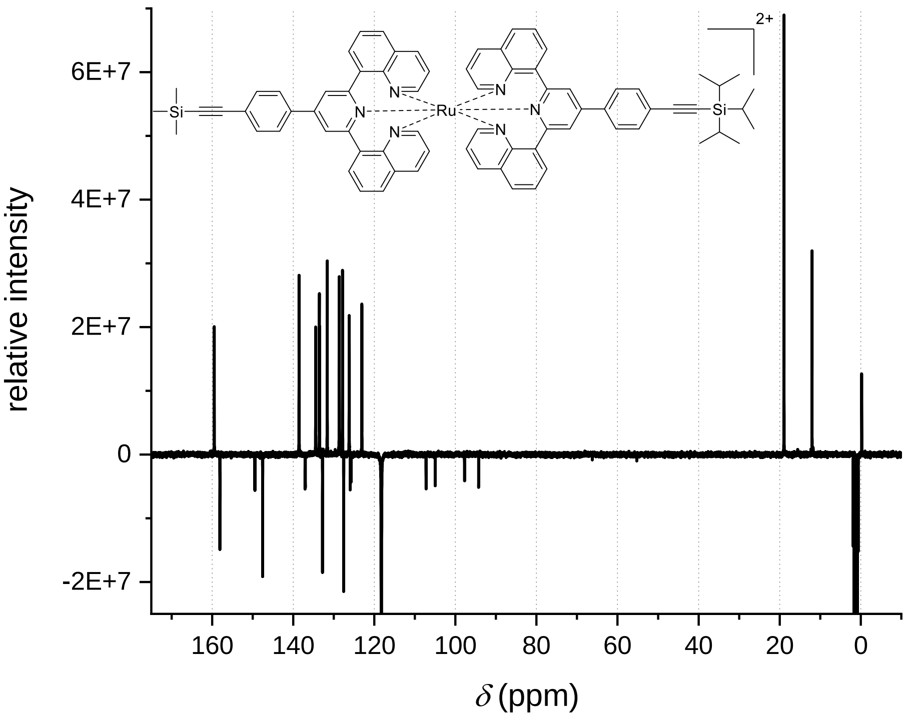

Figure S16. **^{13}C{^{1}H}-APT-NMR spectrum of [Ru(dqpPhCCTMS)(dqpPhCCTIPS)]$^{2+}$ (100 MHz, CD_3CN).**

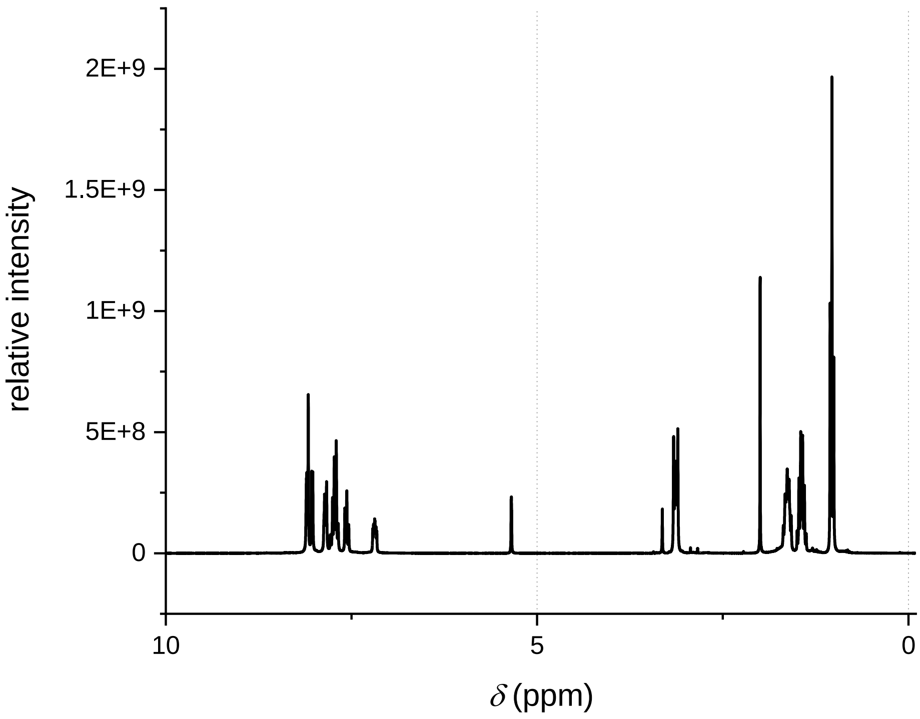

Figure S17. ^{1}H-NMR spectrum of the crude product mixture after deprotection of $[Ru(dqpPhBr)(dqpPhCCTIPS)]^{2+}$ with remainings of $^{n}Bu_4NF$ (300 MHz, CD_2Cl_2).

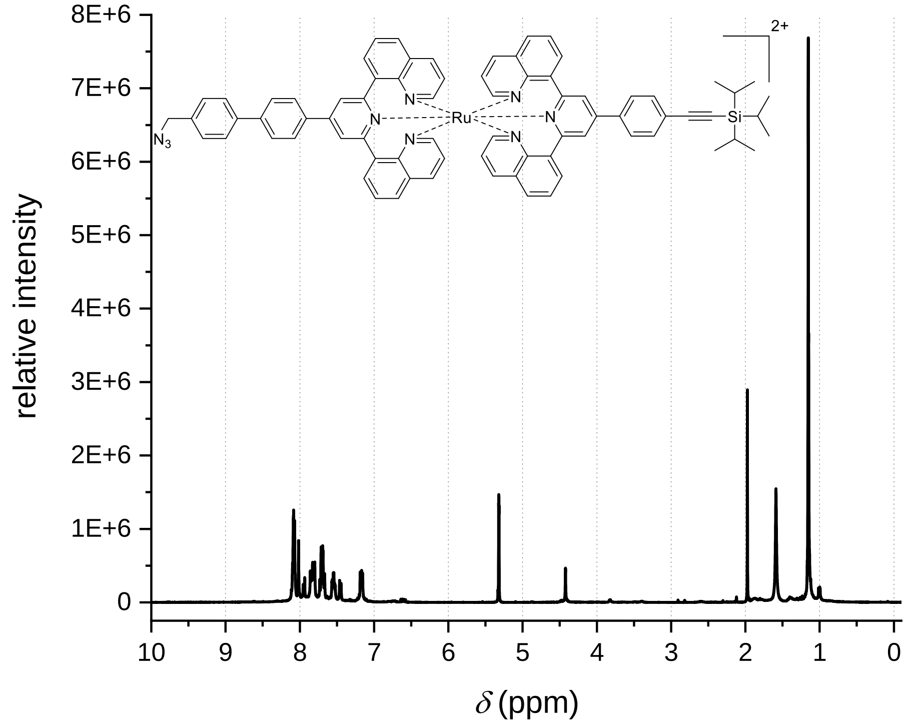

Figure S18. **^{1}H-NMR spectrum of $[Ru(dqpPhPhCH_2N_3)(dqpPhCCTIPS)]^{2+}$ (400 MHz, CD_2Cl_2).**

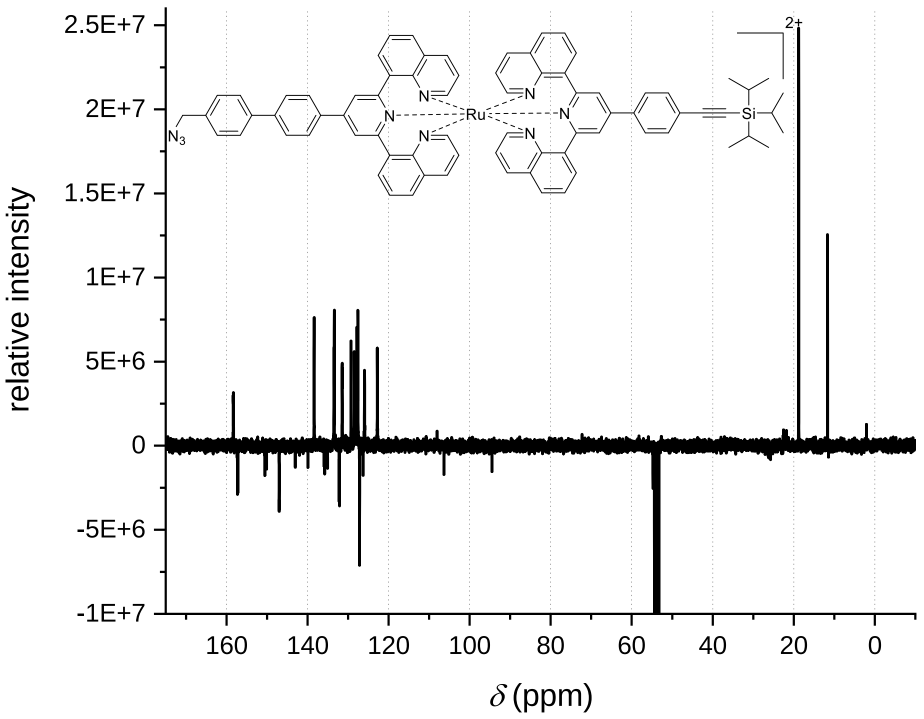

Figure S19. **$^{13}C\{^{1}H\}$-APT-NMR spectrum of $[Ru(dqpPhPhCH_2N_3)(dqpPhCCTIPS)]^{2+}$ (100 MHz, CD_2Cl_2).**

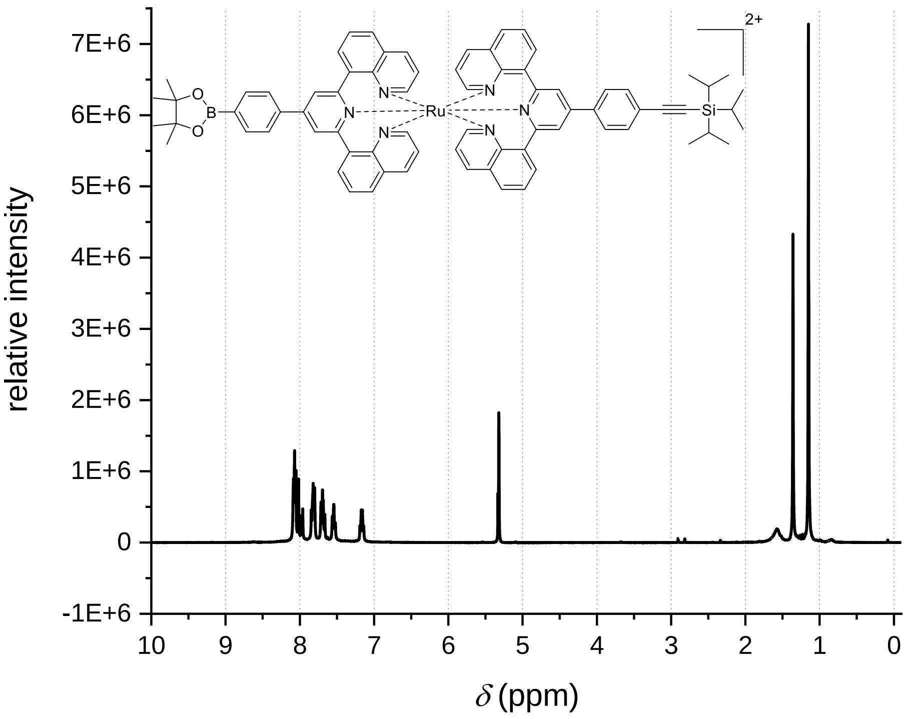

Figure S20. **^{1}H-NMR spectrum of [Ru(dqpPhB(pin))(dqpPhCCTIPS)]$^{2+}$ (400 MHz, CD_2Cl_2).**

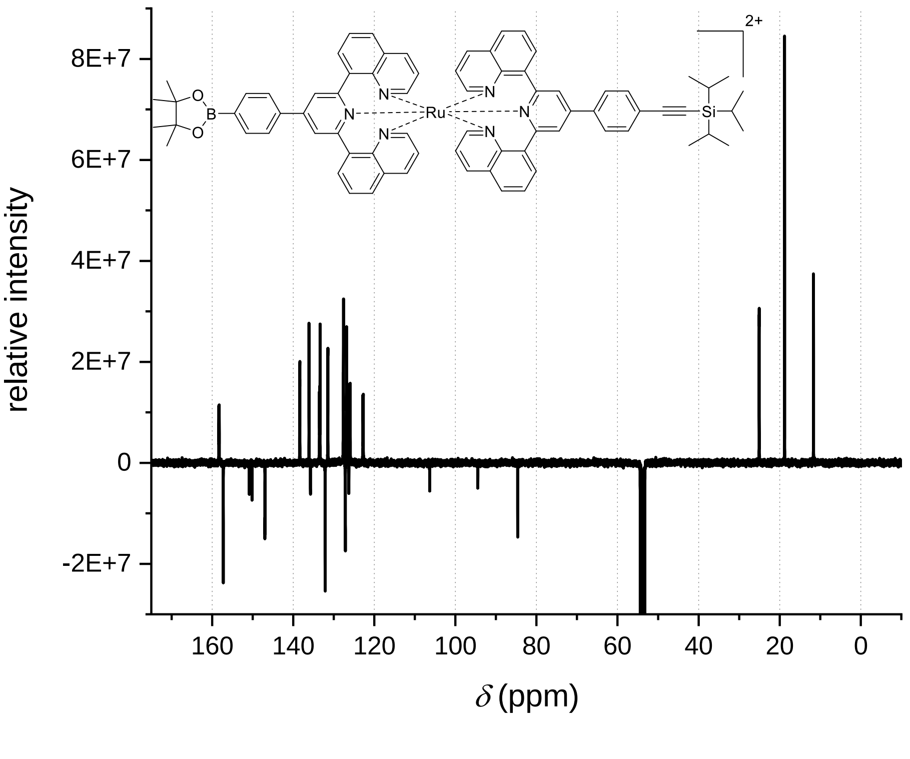

Figure S21. **$^{13}C\{^{1}H\}$-APT-NMR spectrum of [Ru(dqpPhB(pin))(dqpPhCCTIPS)]$^{2+}$ (100 MHz, CD_2Cl_2).**

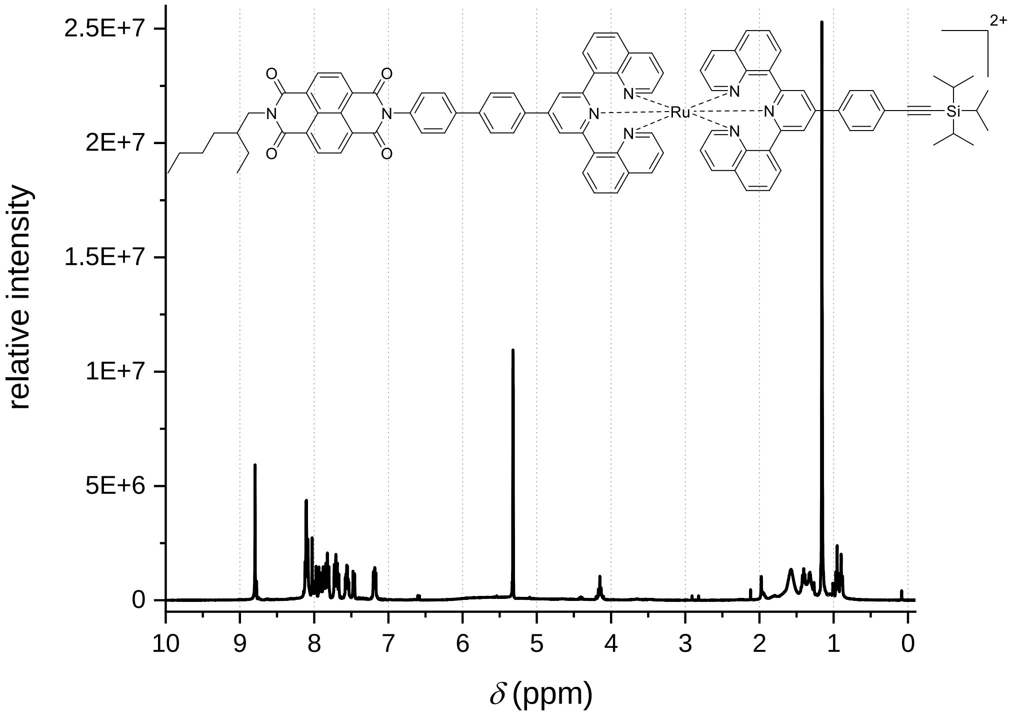

Figure S22. ^{1}**H-NMR spectrum of [Ru(dqpPhPhNDI)(dqpPhCCTIPS)]**$^{2+}$ **(400 MHz,** CD_2Cl_2**).**

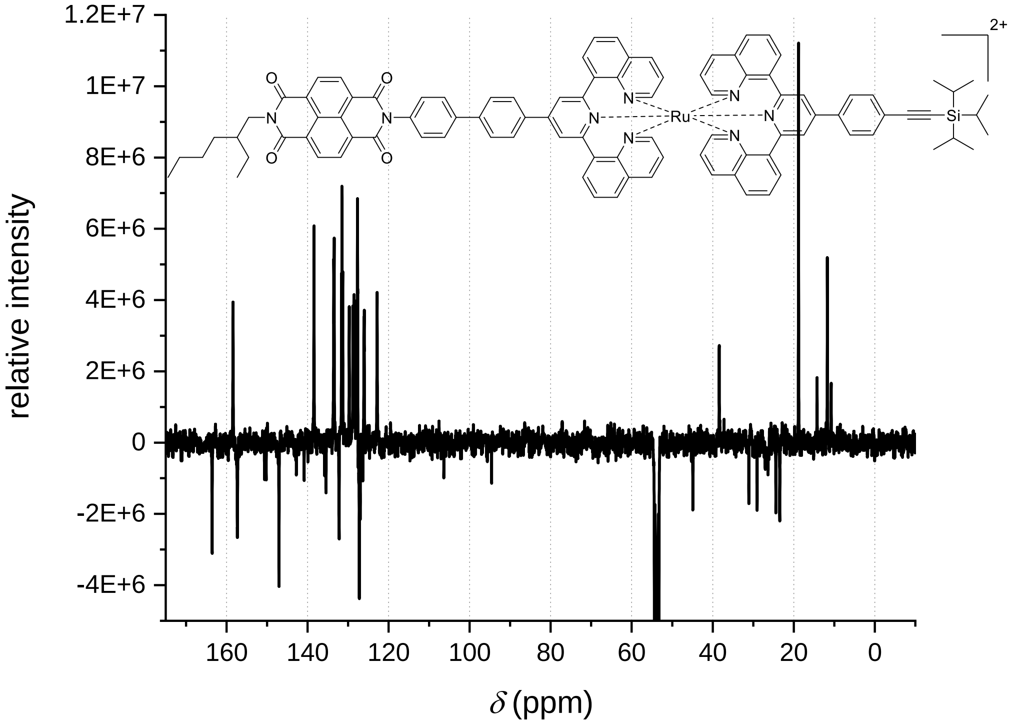

Figure S23. ^{13}**C{**^{1}**H}-APT-NMR spectrum of [Ru(dqpPhPhNDI)(dqpPhCCTIPS)]**$^{2+}$ **(100 MHz,** CD_2Cl_2**).**

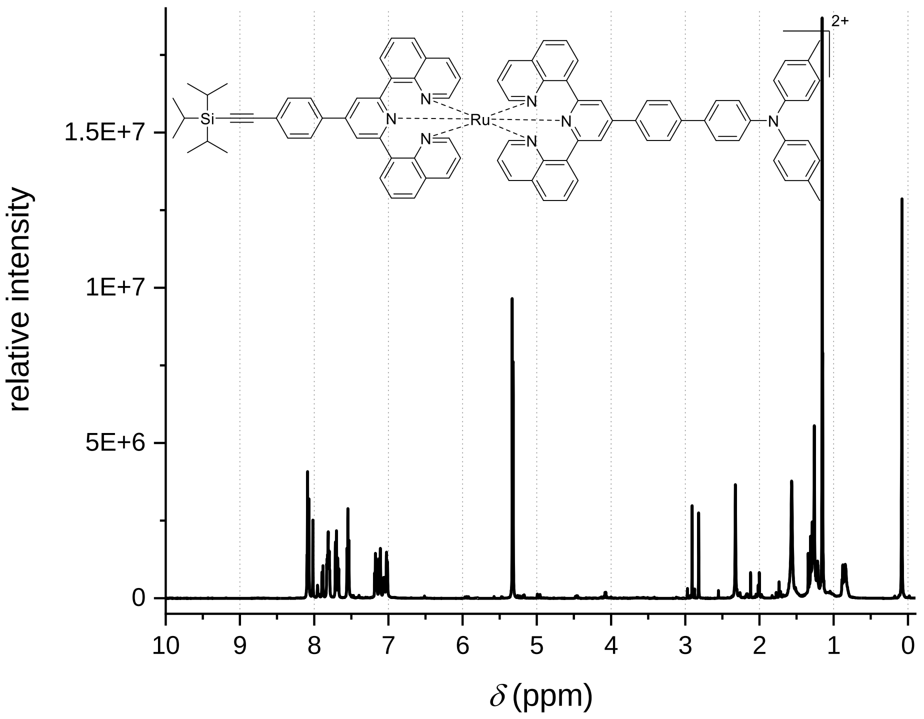

Figure S24. ^{1}H-NMR spectrum of $[Ru(dqpPhCCTIPS)(dqpPhTARA)]^{2+}$ (600 MHz, CD_2Cl_2).

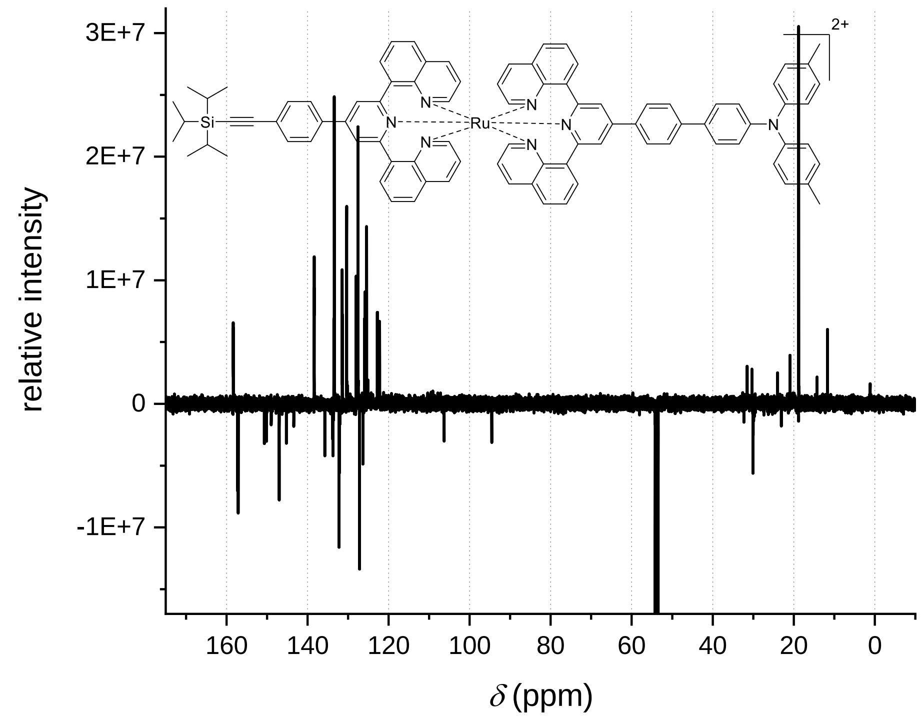

Figure S25. $^{13}C\{^1H\}$-APT-NMR spectrum of $[Ru(dqpPhCCTIPS)(dqpPhTARA)]^{2+}$ (100 MHz, CD_2Cl_2).

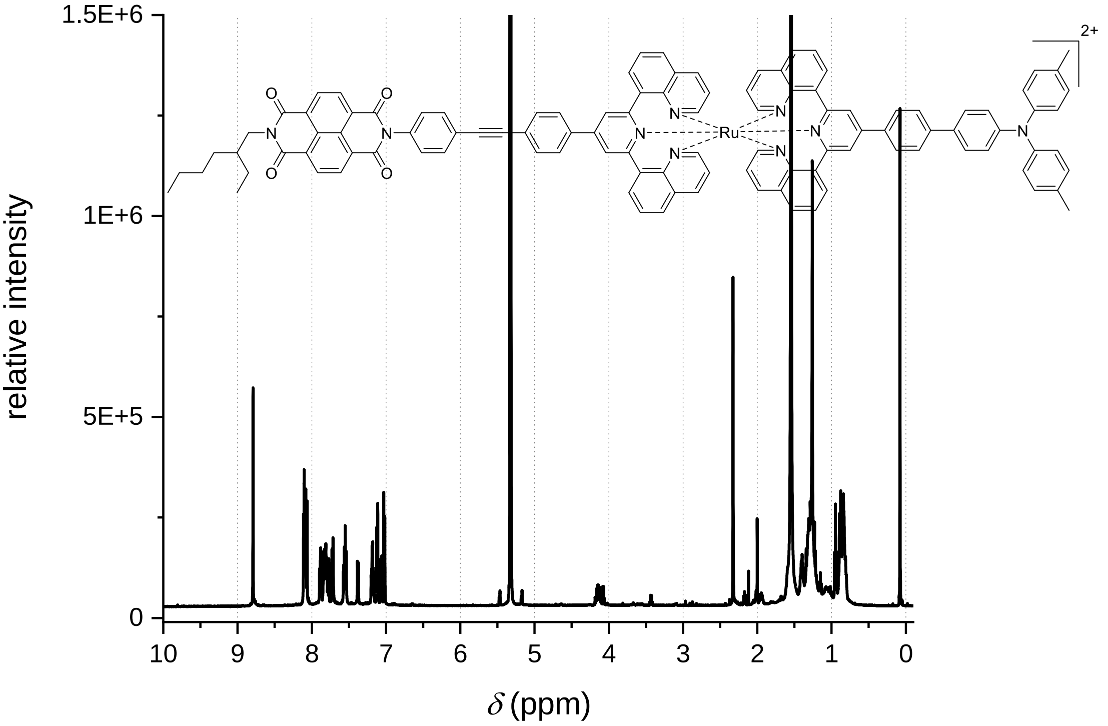

Figure S26. ^{1}H-NMR spectrum of [Ru(dqpPhCCPhNDI)(dqpPhTARA)]$^{2+}$ (600 MHz, CD_2Cl_2).

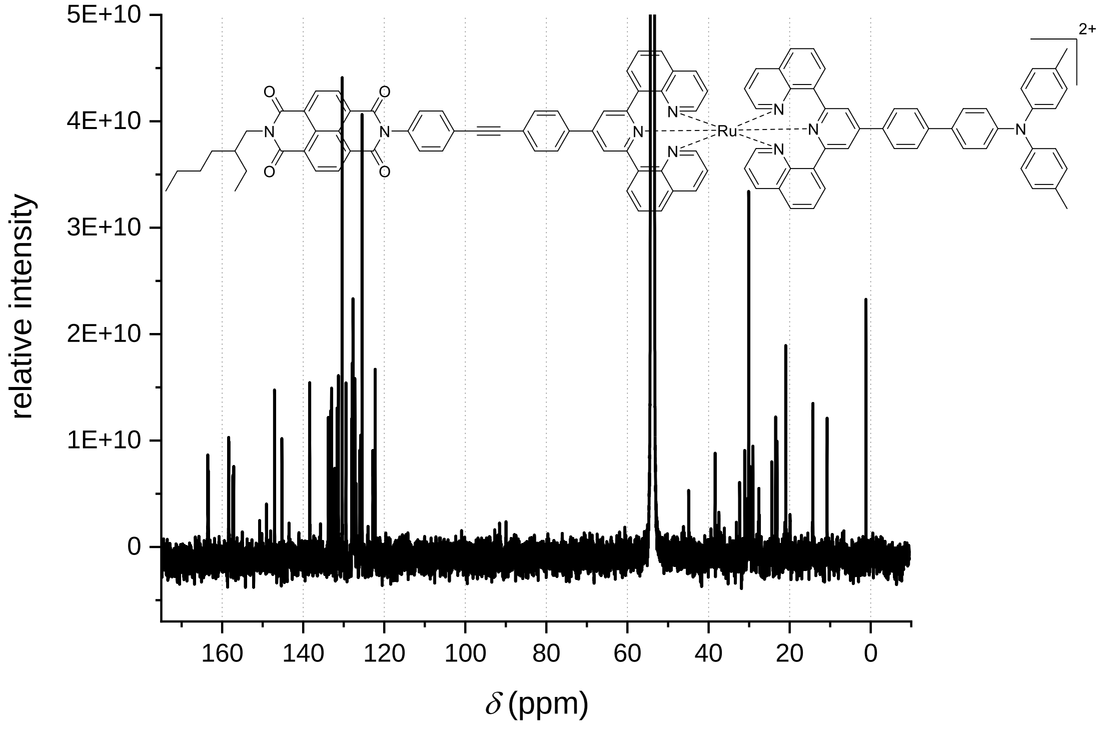

Figure S27. $^{13}C\{^{1}H\}$-NMR spectrum of [Ru(dqpPhCCPhNDI)(dqpPhTARA)]$^{2+}$ (125 MHz, CD_2Cl_2).

ESI-ToF MS data

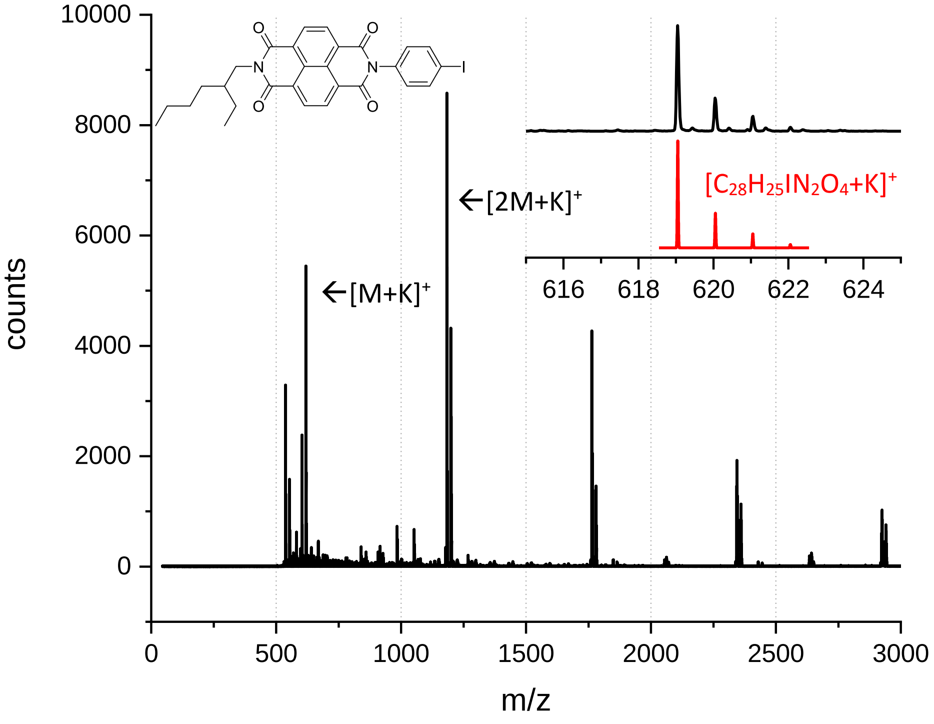

Figure S28. ESI-ToF mass spectrum of NDIPhI. Inset: Section of the mass spectrum with calculated isotope pattern of main species (red).

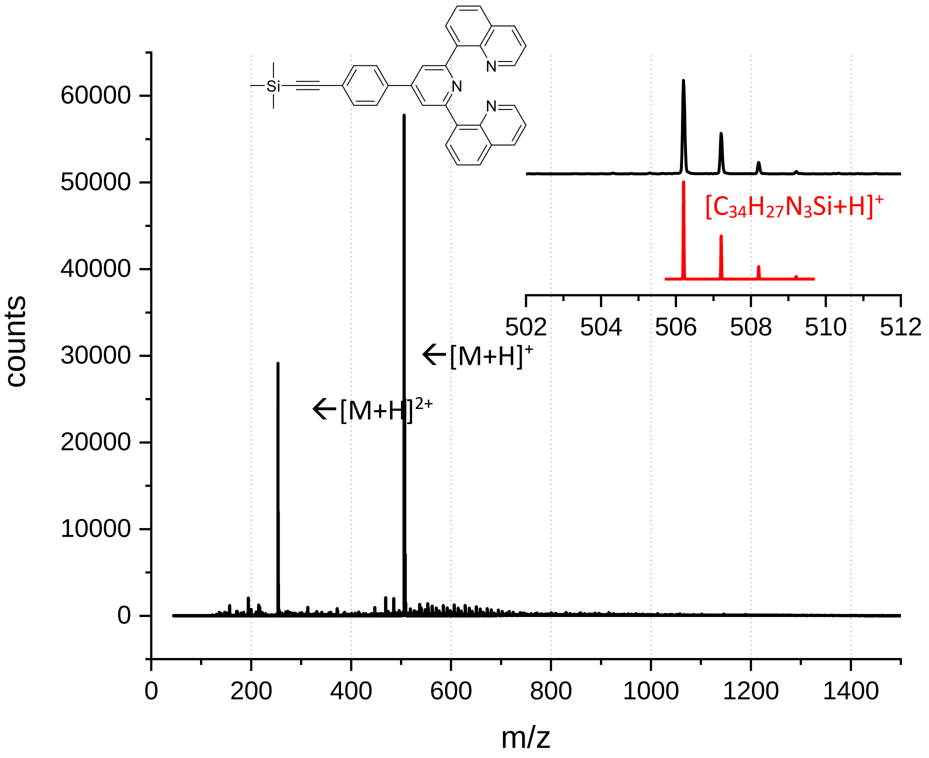

Figure S29. ESI-ToF mass spectrum of dqpPhCCTMS. Inset: Section of the mass spectrum with calculated isotope pattern of main species (red).

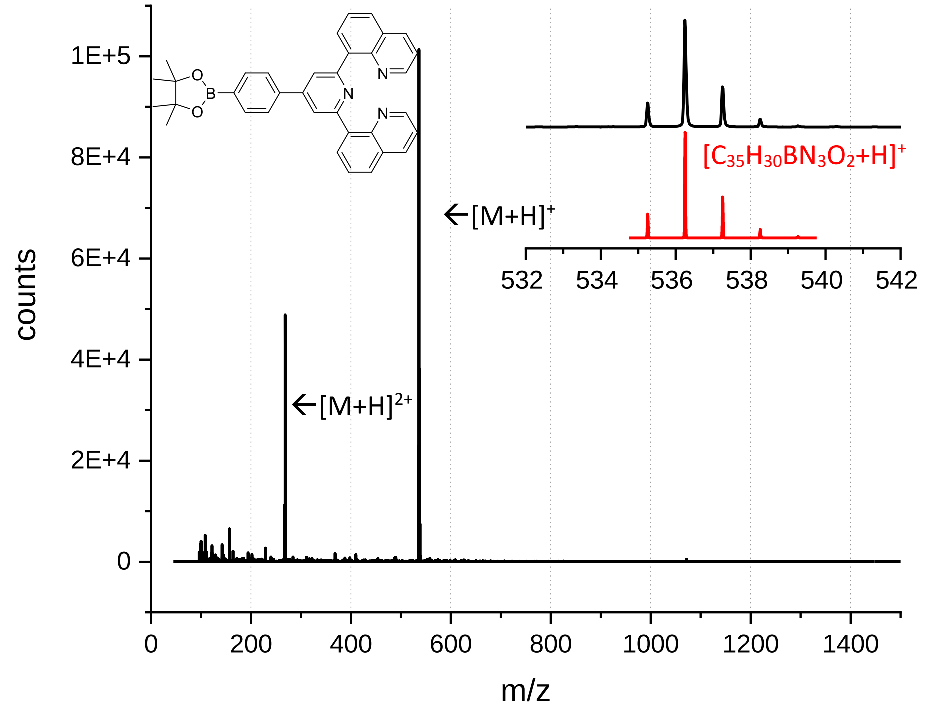

Figure S30. ESI-ToF mass spectrum of dqpPhB(pin). Inset: Section of the mass spectrum with calculated isotope pattern of main species (red).

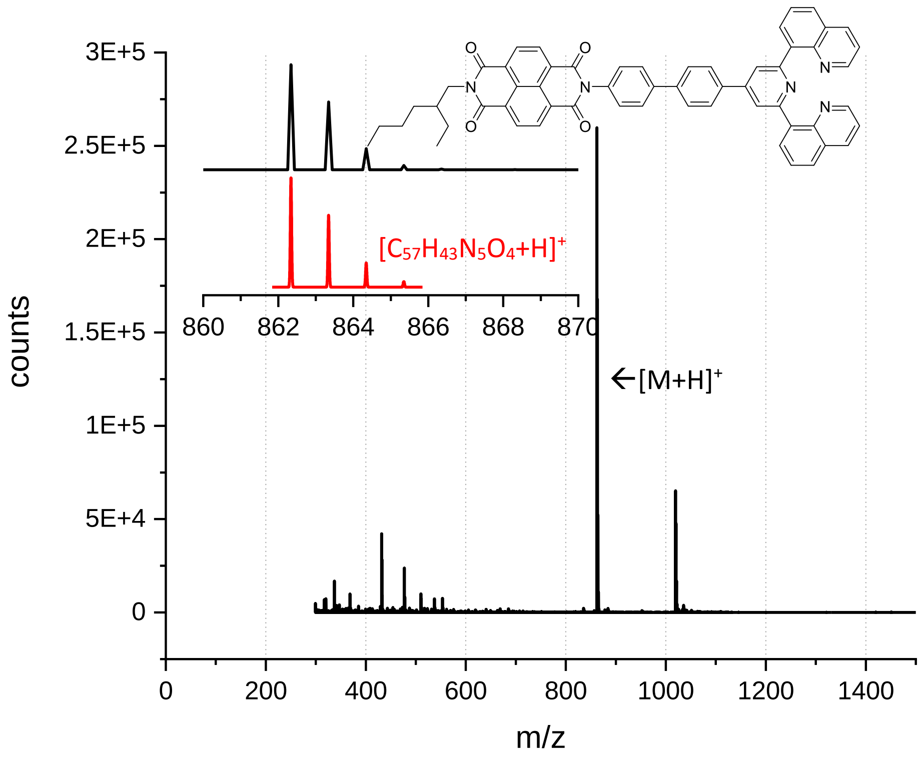

Figure S31. ESI-ToF mass spectrum of dqpPhPhNDI. Inset: Section of the mass spectrum with calculated isotope pattern of main species (red).

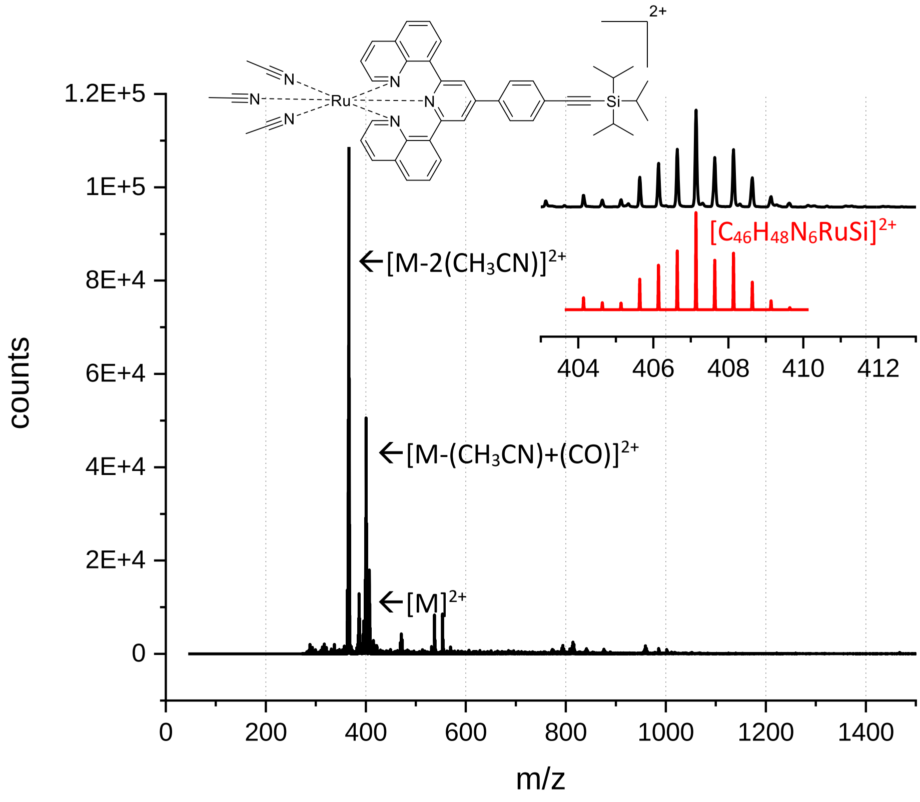

Figure S32. ESI-ToF mass spectrum of $[Ru(dqpPhCCTIPS)(CH_3CN)_3]^{2+}$. Inset: Section of the mass spectrum with calculated isotope pattern of main species (red).

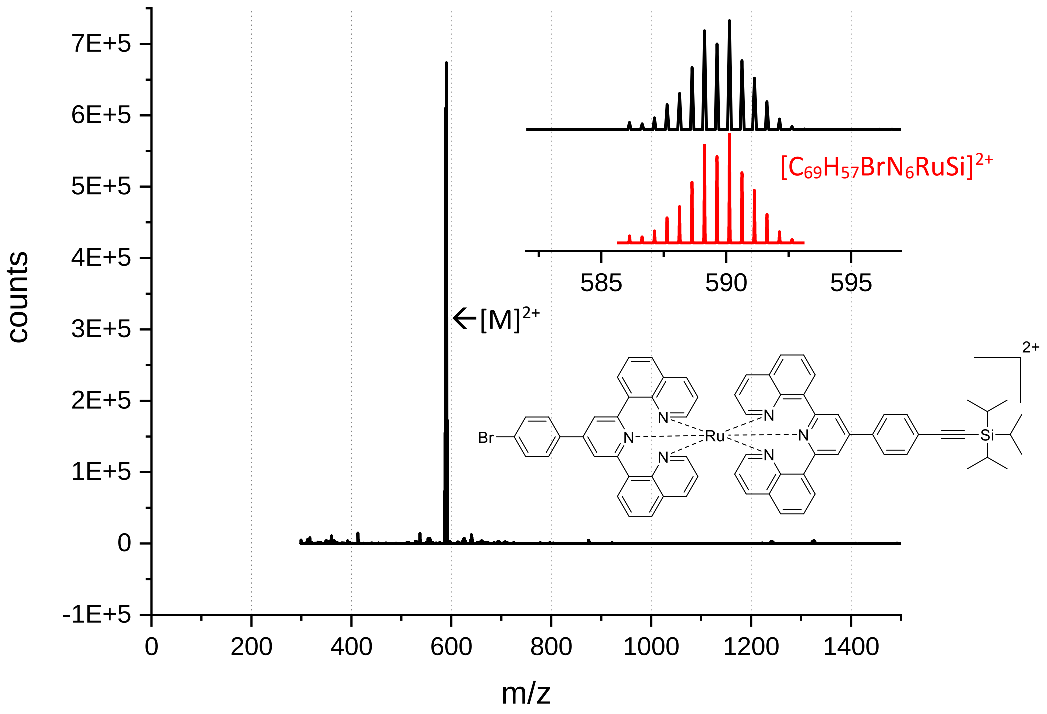

Figure S33. ESI-ToF mass spectrum of [Ru(dqpPhBr)(dqpPhCCTIPS)]$^{2+}$. Inset: Section of the mass spectrum with calculated isotope pattern of main species (red).

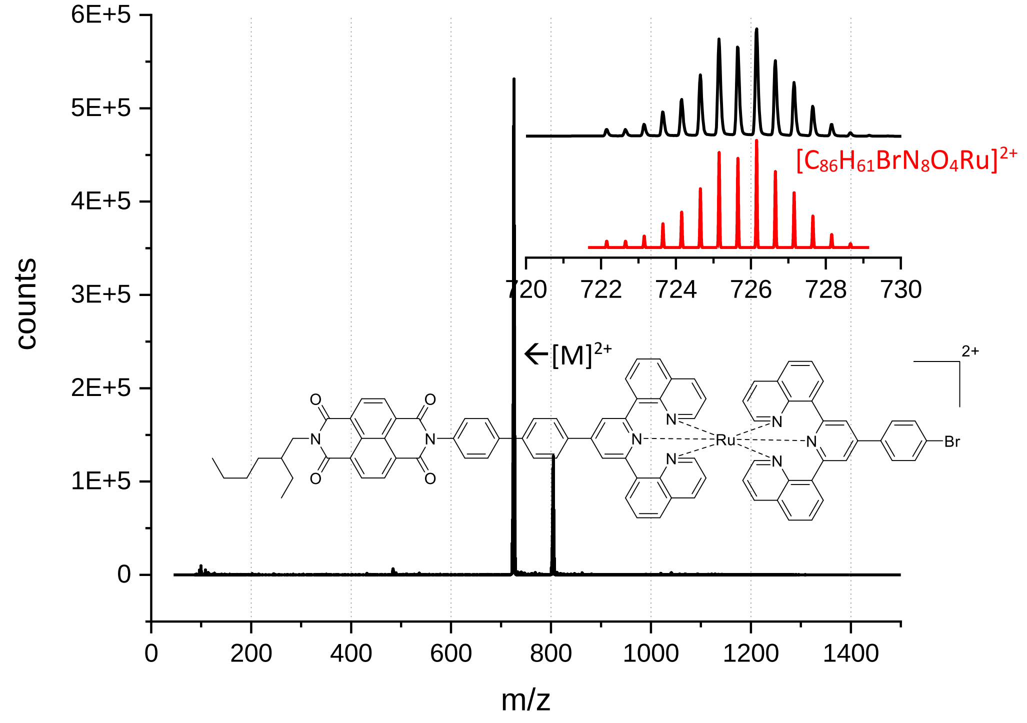

Figure S34. ESI-ToF mass spectrum of [Ru(dqpPhPhNDIBr)(dqpPhBr)]$^{2+}$. Inset: Section of the mass spectrum with calculated isotope pattern of main species (red).

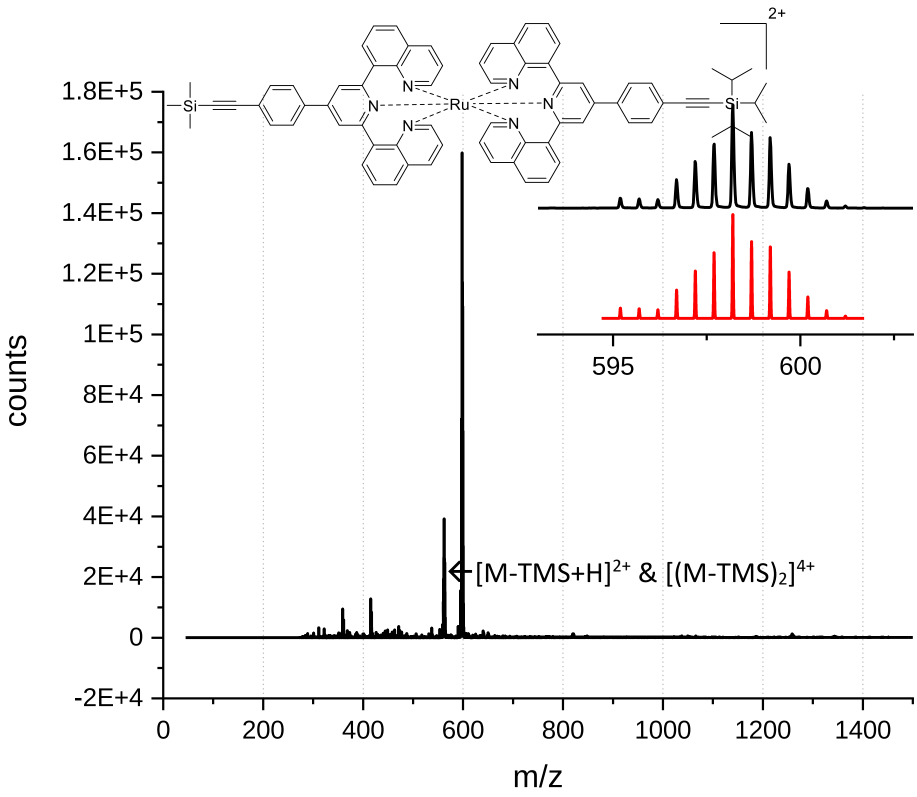

Figure S35. ESI-ToF mass spectrum of $[Ru(dqpPhCCTMS)(dqpPhCCTIPS)]^{2+}$. Inset: Section of the mass spectrum with calculated isotope pattern of main species (red).

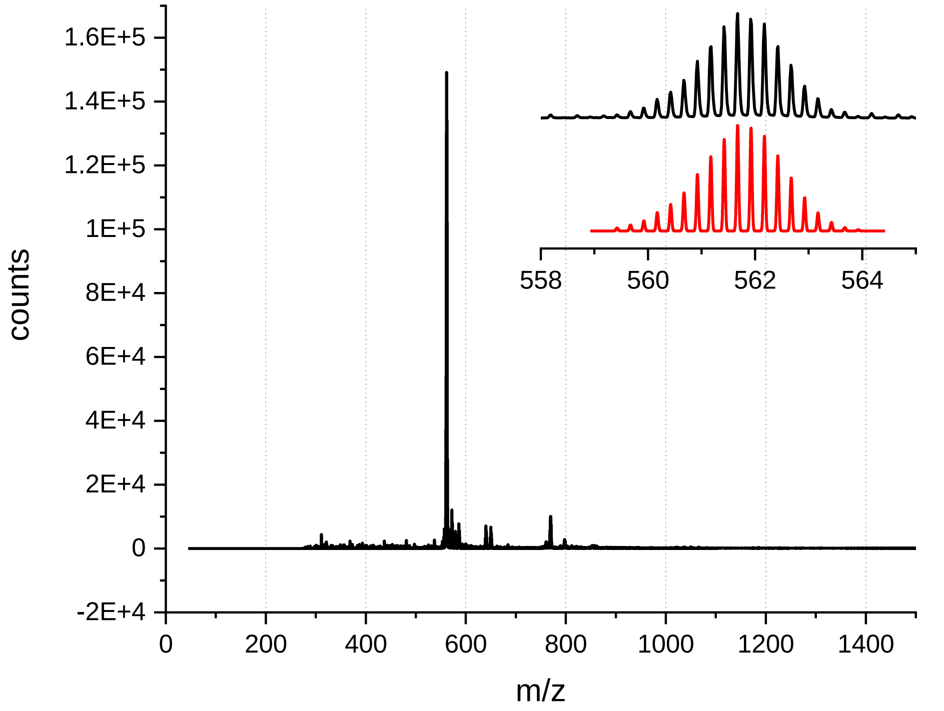

Figure S36. ESI-ToF mass spectrum of the crude product mixture after deprotection of [Ru(dqpPhCCTMS)(dqpPhCCTIPS)]$^{2+}$ with K_2CO_3. Inset: Section of the mass spectrum with calculated isotope pattern of main species (red).

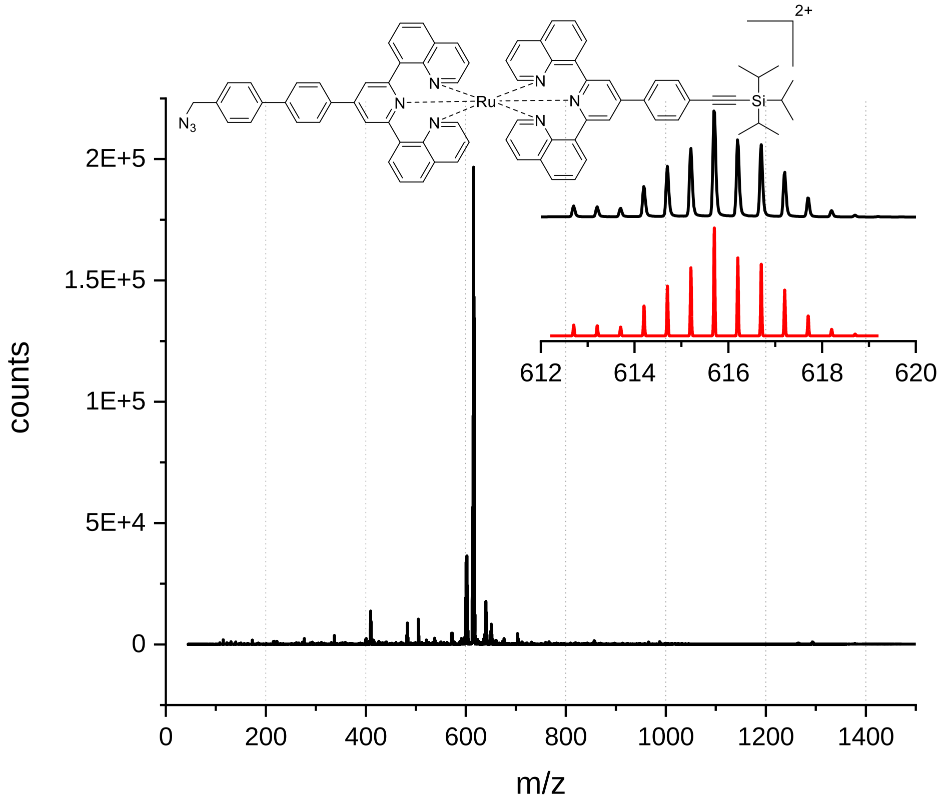

Figure S37. ESI-ToF mass spectrum of [Ru(dqpPhPhCH$_2$N$_3$)(dqpPhCCTIPS)]$^{2+}$. Inset: Section of the mass spectrum with calculated isotope pattern of main species (red).

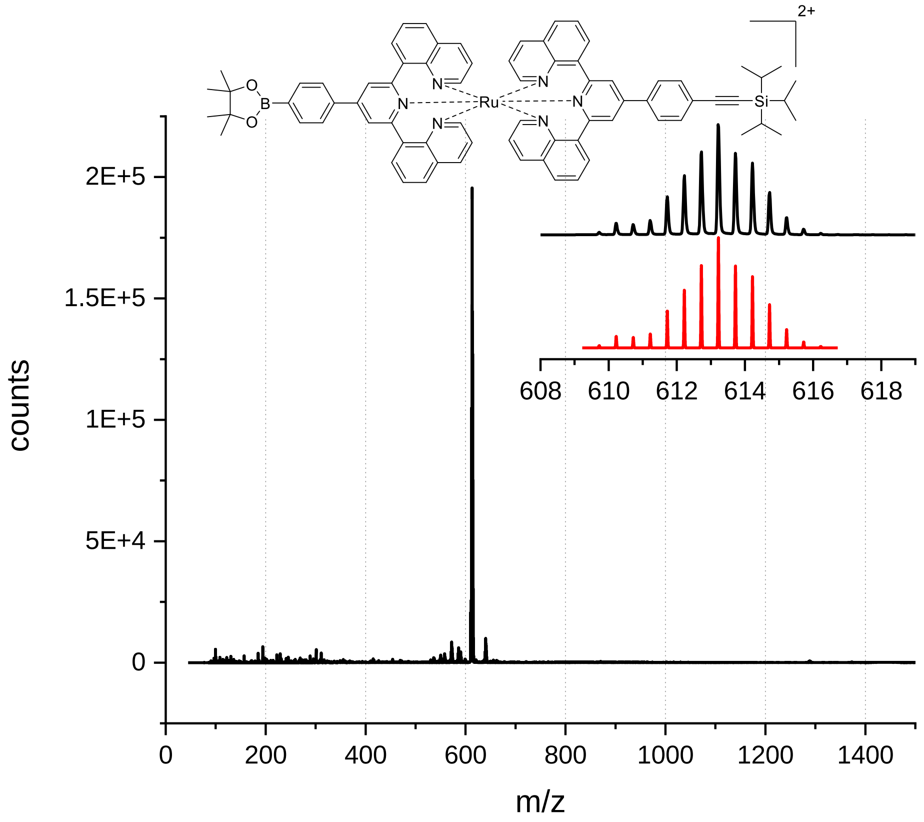

Figure S38. ESI-ToF mass spectrum of [Ru(dqpPhB(pin))(dqpPhCCTIPS)]$^{2+}$. Inset: Section of the mass spectrum with calculated isotope pattern of main species (red).

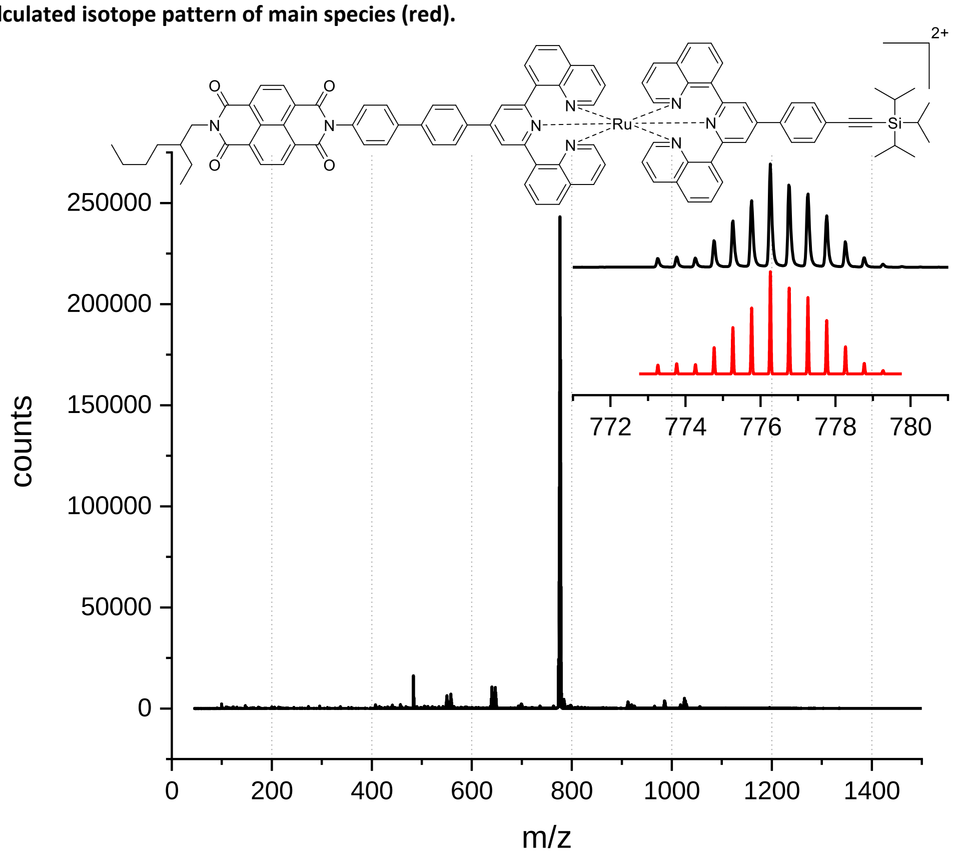

Figure S39. ESI-ToF mass spectrum of [Ru(dqpPhPhNDI)(dqpPhCCTIPS)]$^{2+}$. Inset: Section of the mass spectrum with calculated isotope pattern of main species (red).

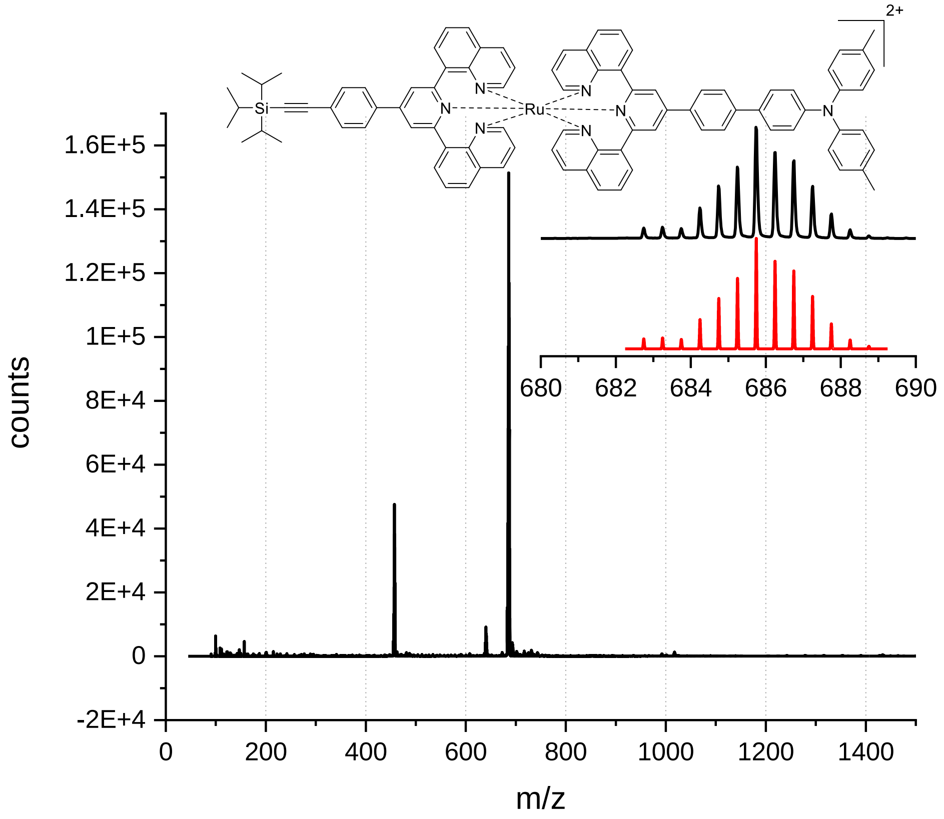

Figure S40. ESI-ToF mass spectrum of [Ru(dqpPhPhTARA)(dqpPhCCTIPS)]$^{2+}$. Inset: Section of the mass spectrum with calculated isotope pattern of main species (red).

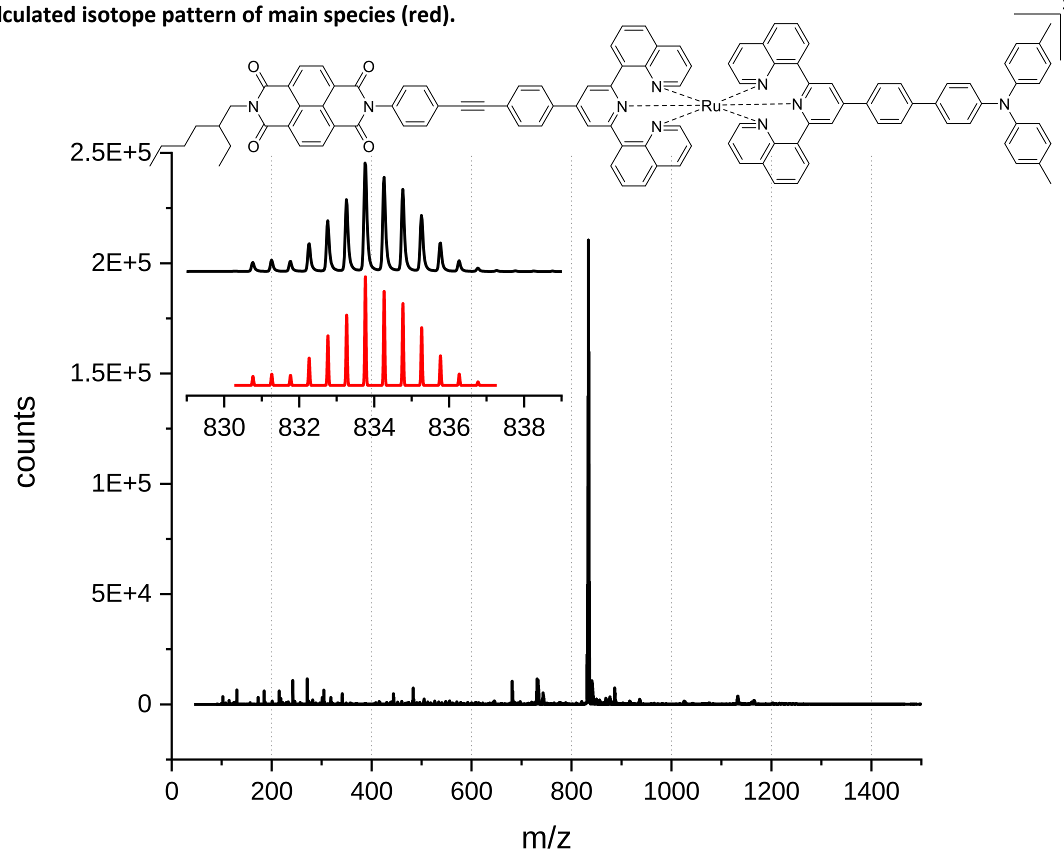

Figure S41. ESI-ToF mass spectrum of [Ru(dqpPhPhTARA)(dqpPhCCPhNDI)]$^{2+}$. Inset: Section of the mass spectrum with calculated isotope pattern of main species (red).

SEC data

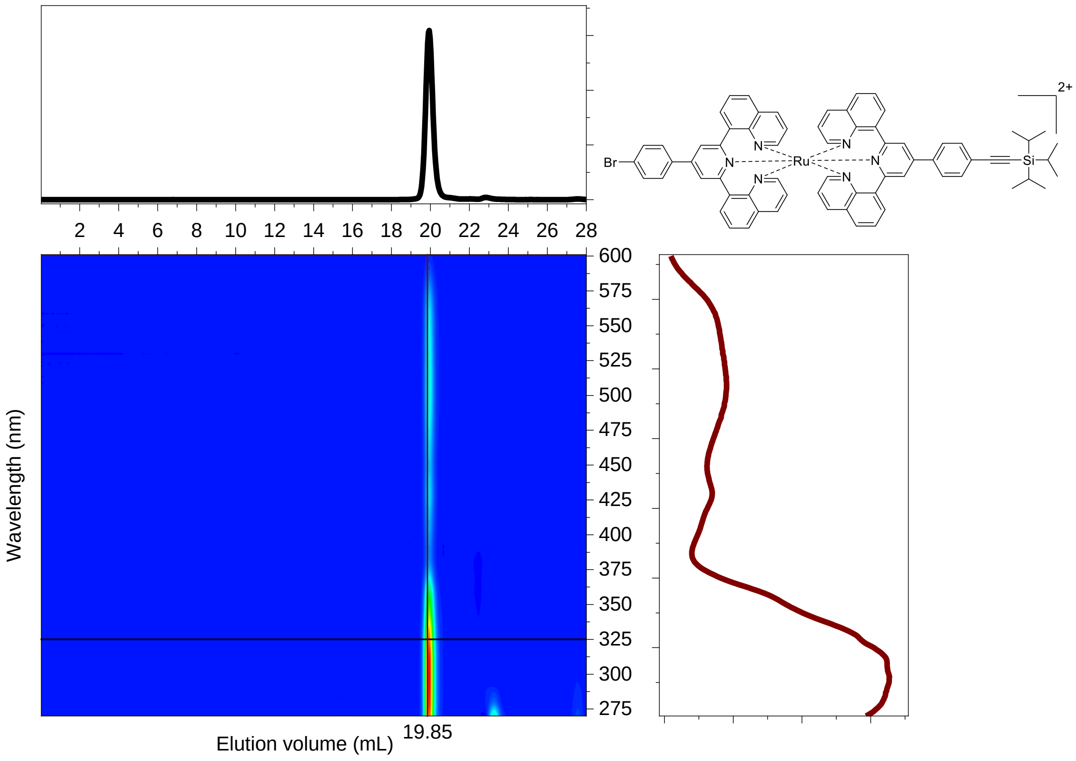

Figure S42. SEC elugram of [Ru(dqpPhBr)(dqpPhCCTIPS)]$^{2+}$ with extracted elugram trace at 325 nm (top) and extracted UV/Vis-data at 19.85 mL (right) (DMAc + 0.08 wt% NH_4PF_6).

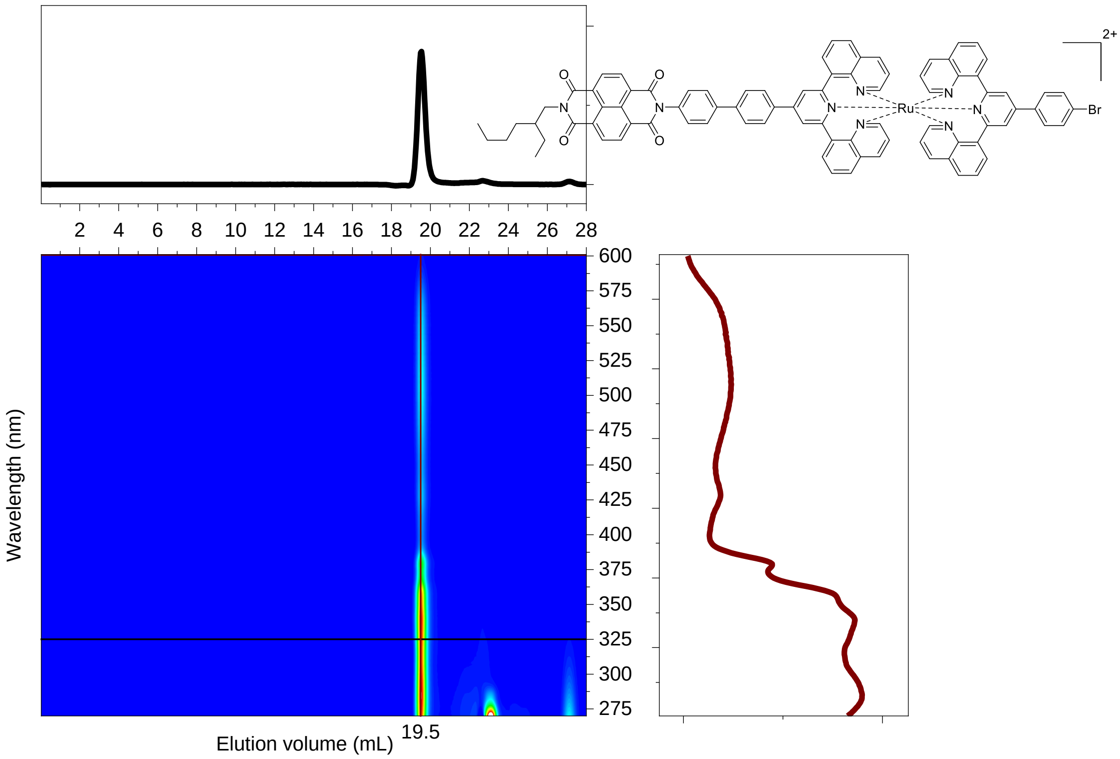

Figure S43. SEC elugram of [Ru(dqpPhPhNDI)(dqpPhBr)]$^{2+}$ with extracted elugram trace at 325 nm (top) and extracted UV/Vis-data at 19.50 mL (right) (DMAc + 0.08 wt% NH_4PF_6).

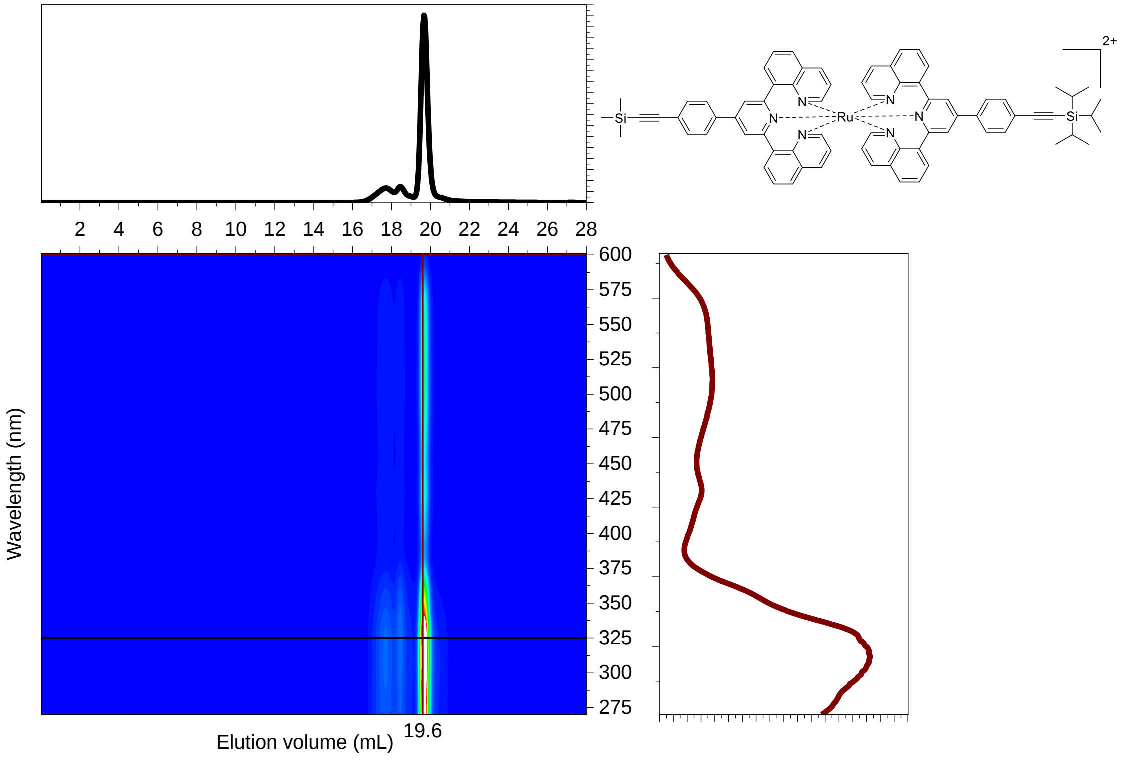

Figure S44. SEC elugram of [Ru(dqpPhCCTMS)(dqpPhCCTIPS)]$^{2+}$ with extracted elugram trace at 325 nm (top) and extracted UV/Vis-data at 19.6 mL (right) (DMAc + 0.08 wt% NH_4PF_6).

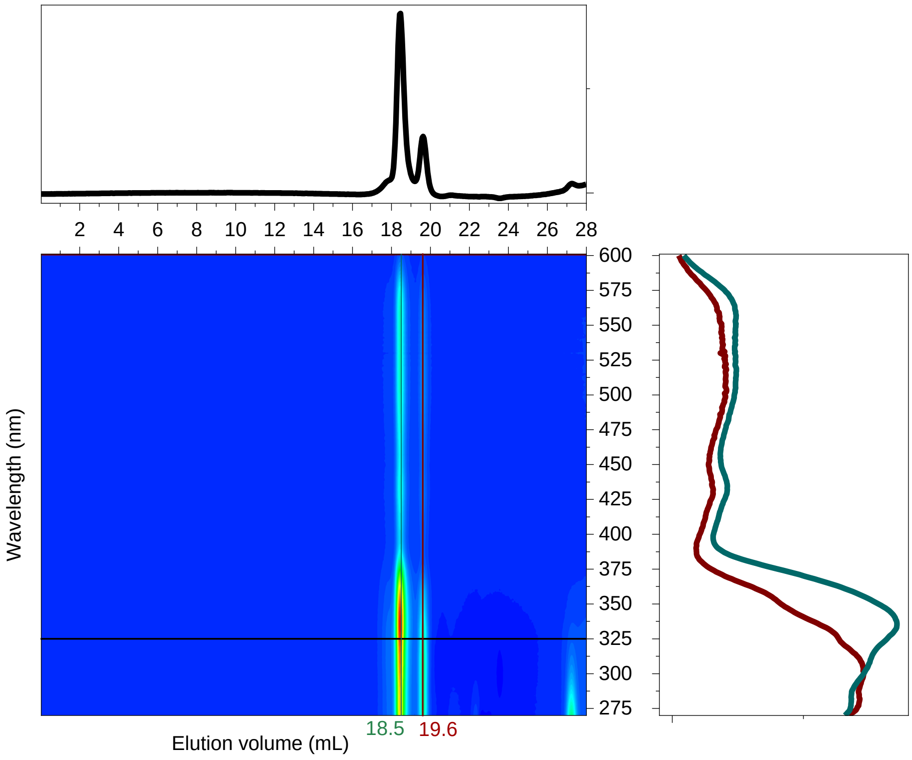

Figure S45. SEC elugram of the crude product mixture after deprotection of [Ru(dqpPhCCTMS)(dqpPhCCTIPS)]$^{2+}$ with K_2CO_3 with extracted elugram trace at 325 nm (top) and extracted UV/Vis-data at 19.6 mL (red) and 18.5 mL (green, right) (DMAc + 0.08 wt% NH_4PF_6).

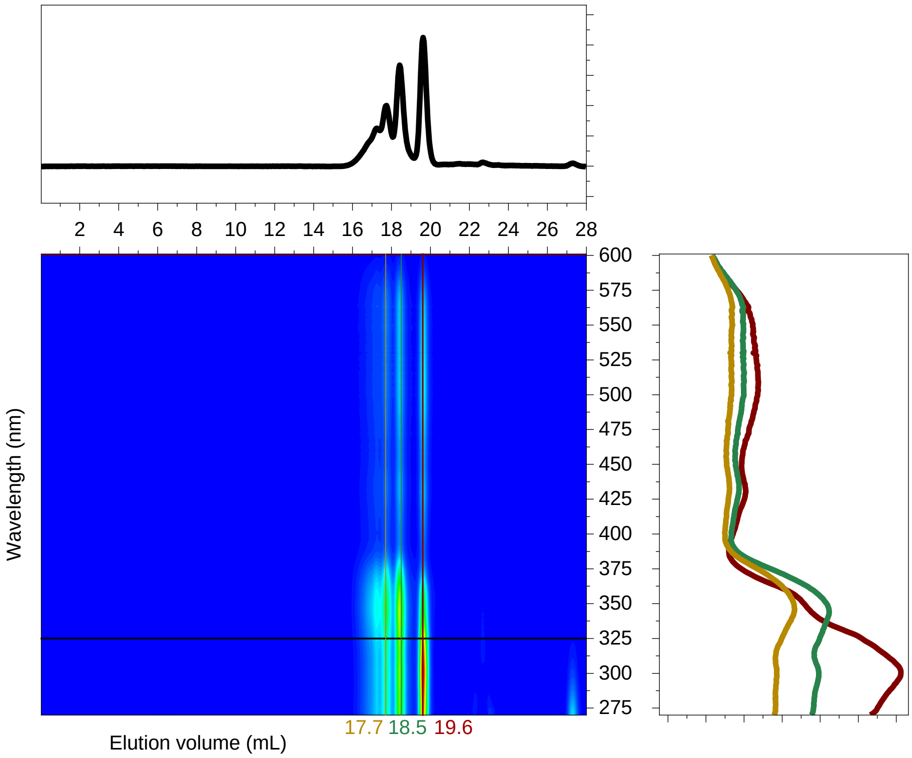

Figure S46. SEC elugram of the crude product mixture after deprotection of [Ru(dqpPhCCTMS)(dqpPhCCTIPS)]$^{2+}$ with $^{n}Bu_4NF$ with extracted elugram trace at 325 nm (top) and extracted UV/Vis-data at 19.6 mL (red), 18.5 mL (green), and 17.7 mL (yellow, right) (DMAc + 0.08 wt% NH_4PF_6).

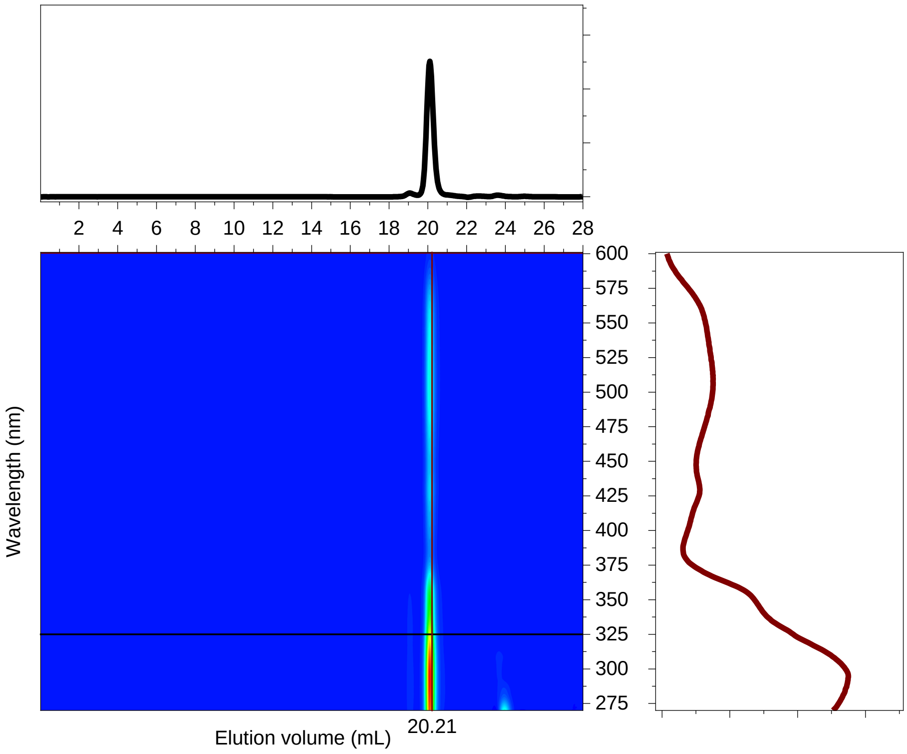

Figure S47. SEC elugram of the crude product mixture after deprotection of [Ru(dqpPhBr)(dqpPhCCTIPS)]$^{2+}$ with $^{n}Bu_4NF$ with extracted elugram trace at 325 nm (top) and extracted UV/Vis-data at 20.21 mL (right) (DMAc + 0.08 wt% NH_4PF_6).

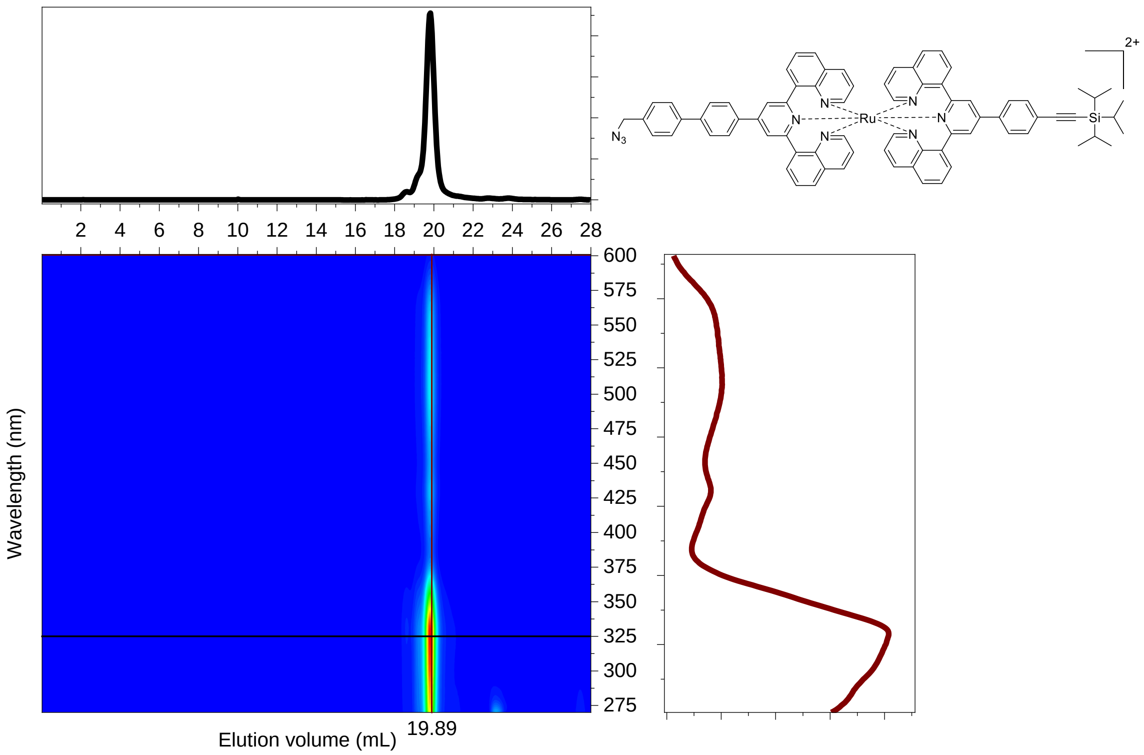

Figure S48. SEC elugram of [Ru(dqpPhPhCH$_2$N$_3$)(dqpPhCCTIPS)]$^{2+}$ with extracted elugram trace at 325 nm (top) and extracted UV/Vis-data at 19.89 mL (right) (DMAc + 0.08 wt% NH$_4$PF$_6$).

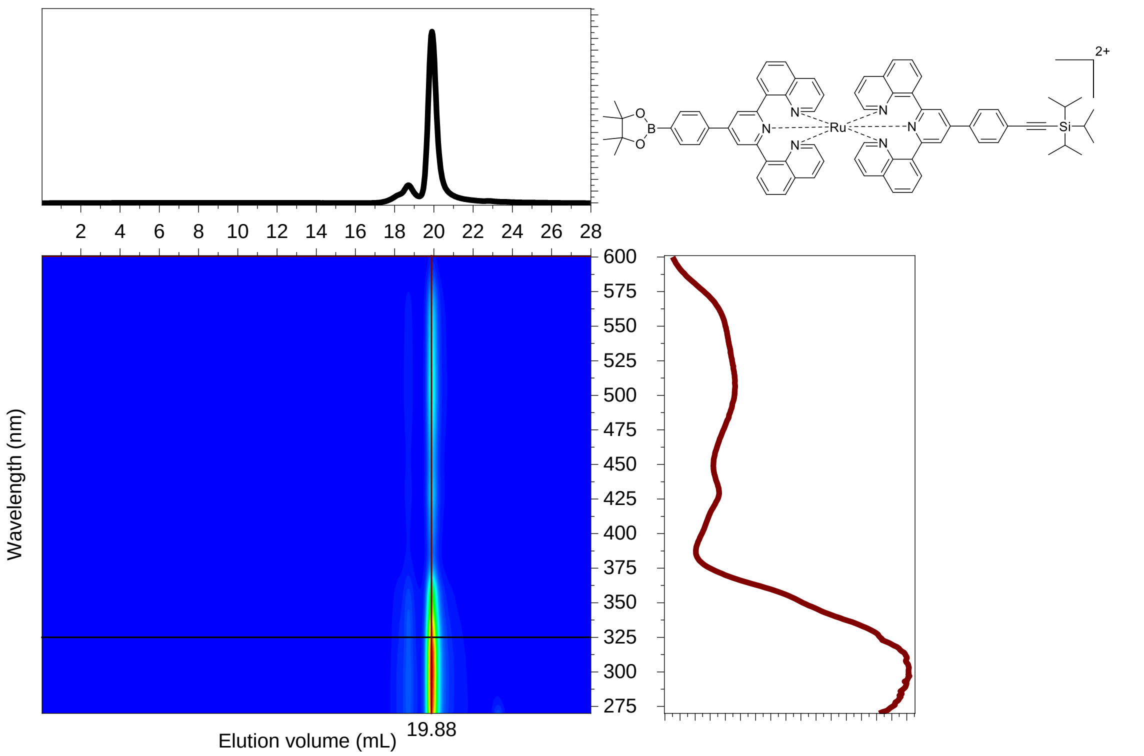

Figure S49. SEC elugram of [Ru(dqpPhB(pin))(dqpPhCCTIPS)]$^{2+}$ with extracted elugram trace at 325 nm (top) and extracted UV/Vis-data at 19.88 mL (right) (DMAc + 0.08 wt% NH$_4$PF$_6$).

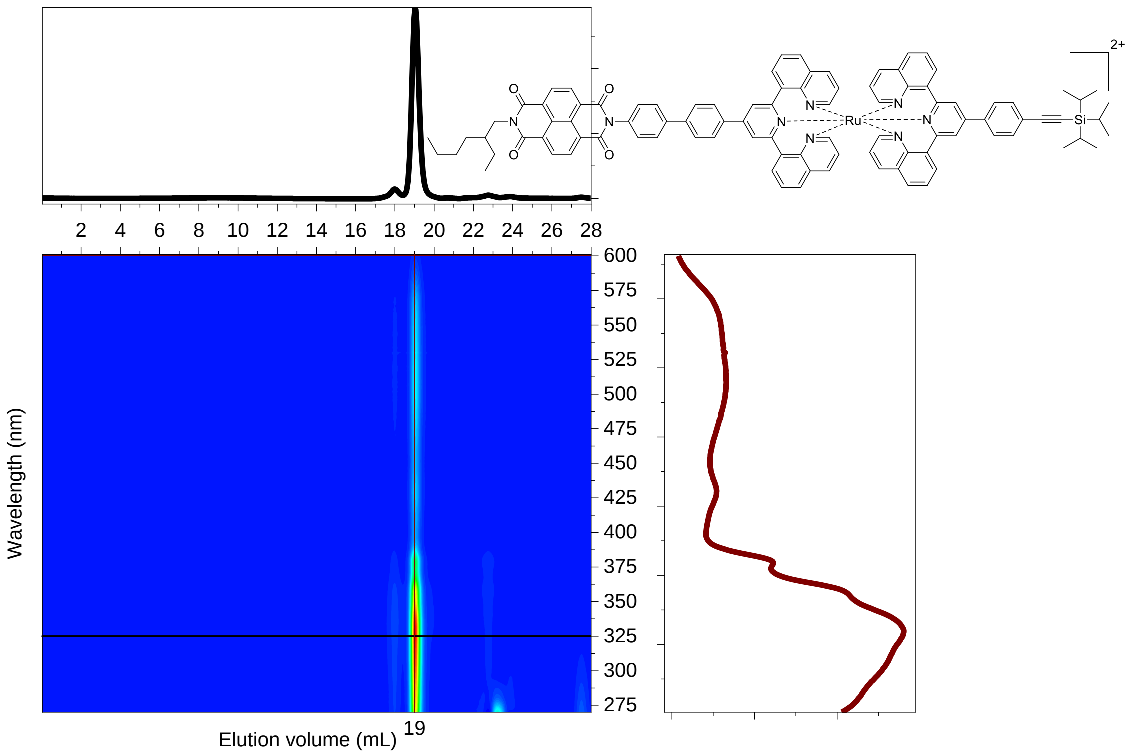

Figure S50. SEC elugram of [Ru(dqpPhPhNDI)(dqpPhCCTIPS)]$^{2+}$ with extracted elugram trace at 325 nm (top) and extracted UV/Vis-data at 19.00 mL (right) (DMAc + 0.08 wt% NH_4PF_6).

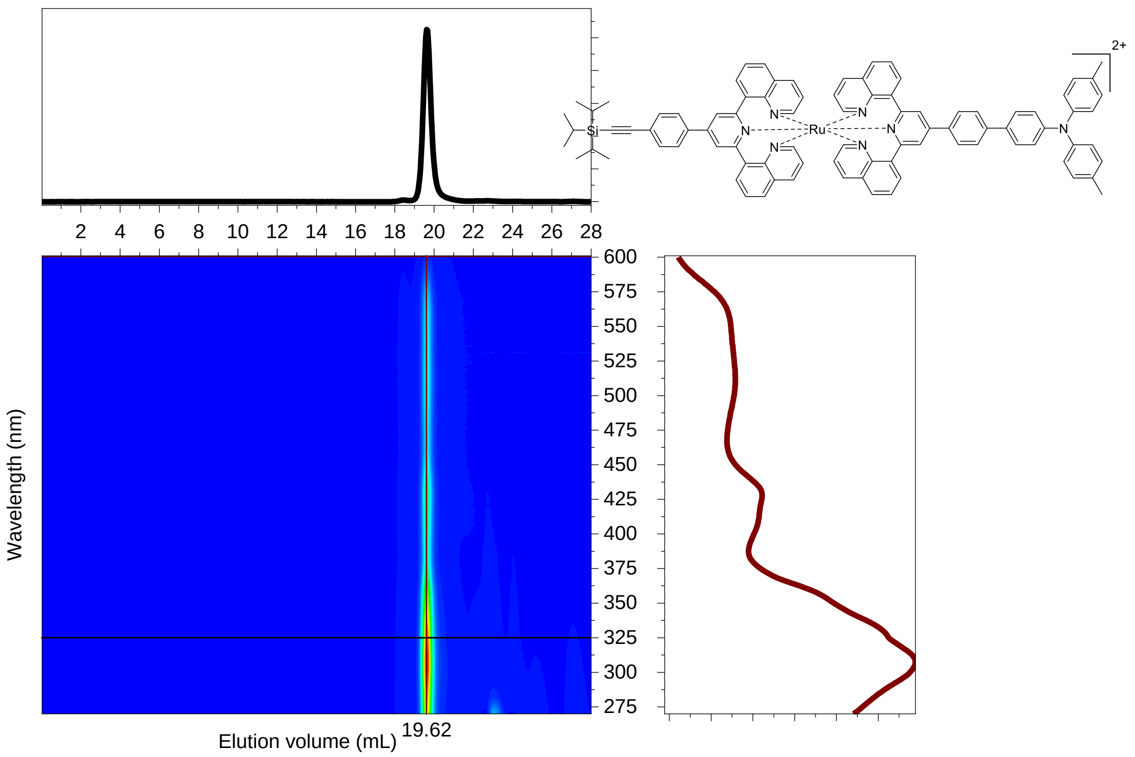

Figure S51. SEC elugram of [Ru(dqpPhCCTIPS)(dqpPhTARA)]$^{2+}$ with extracted elugram trace at 325 nm (top) and extracted UV/Vis-data at 19.62 mL (right) (DMAc + 0.08 wt% NH_4PF_6).

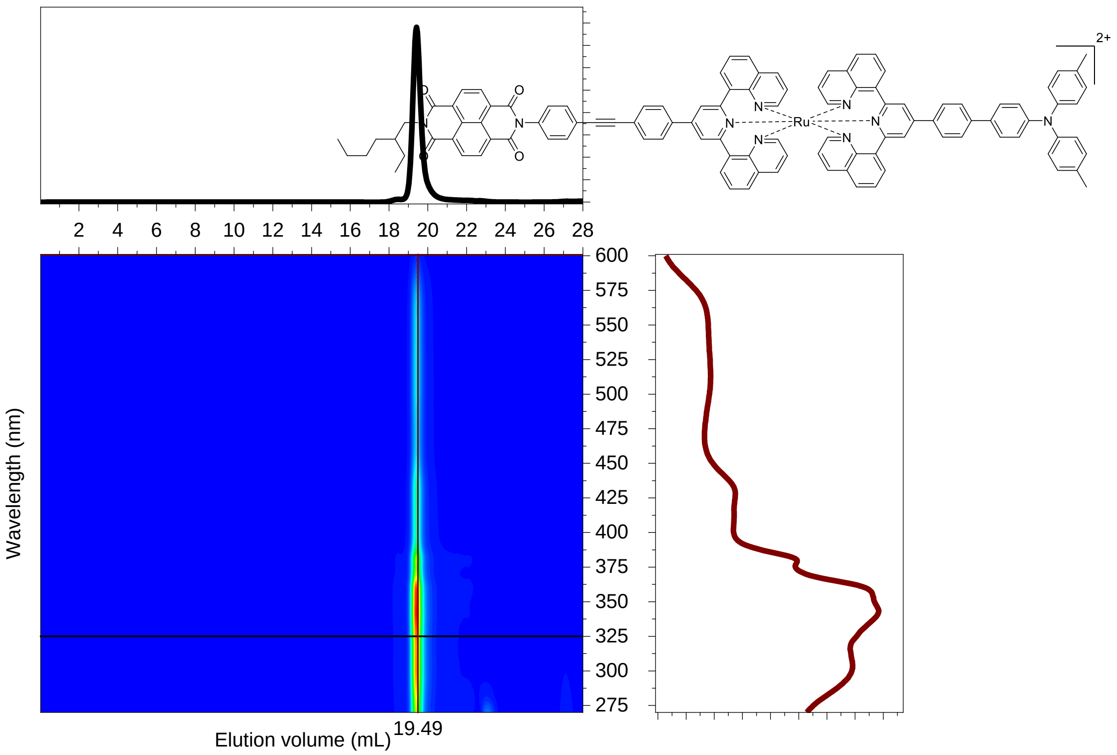

Figure S52. SEC elugram of [Ru(dqpPhTARA)(dqpPhCCPhNDI)]$^{2+}$ with extracted elugram trace at 325 nm (top) and extracted UV/Vis-data at 19.49 mL (right) (DMAc + 0.08 wt% NH_4PF_6).

Absorption and emission spectra

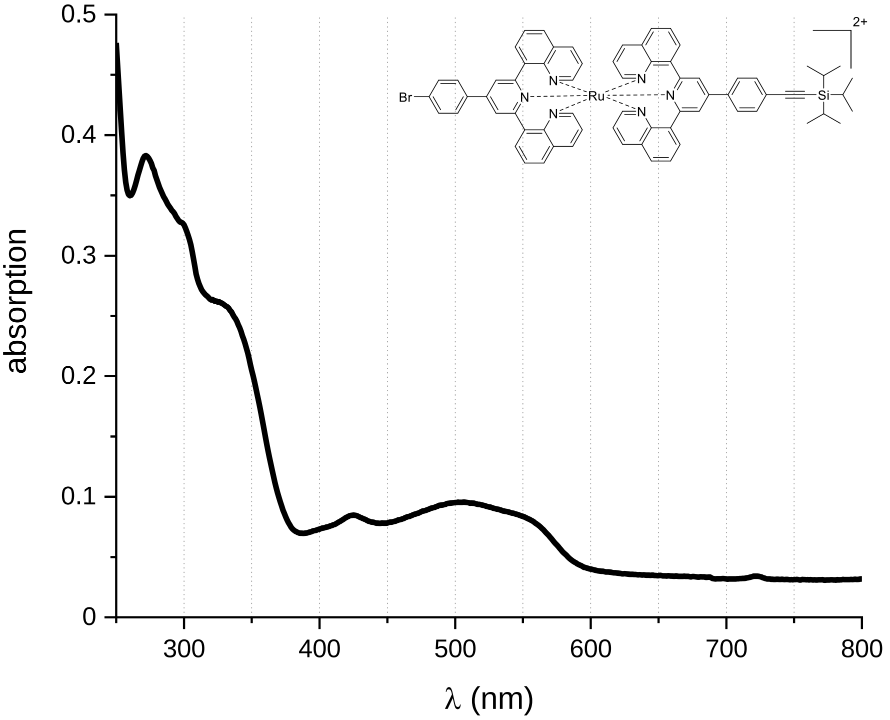

Figure S53. Absorption spectrum of [Ru(dqpPhBr)(dqpPhCCTIPS)]$^{2+}$ in dry CH_2Cl_2 *vs*. pure CH_2Cl_2.

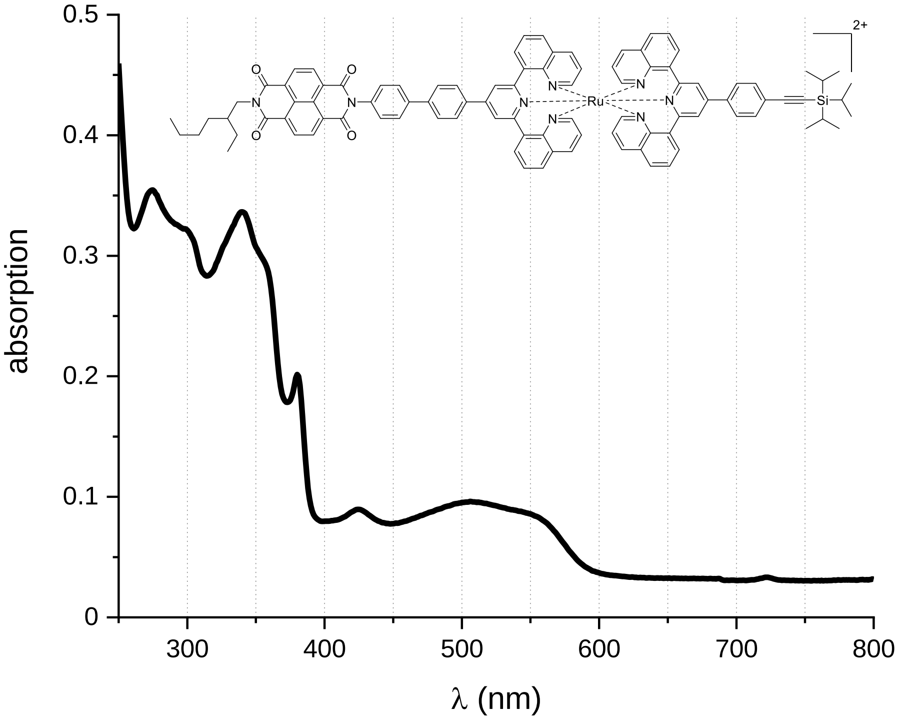

Figure S54. Absorption spectrum of [Ru(dqpPhPhNDI)(dqpPhCCTIPS)]$^{2+}$ in dry CH_2Cl_2 *vs*. pure CH_2Cl_2.

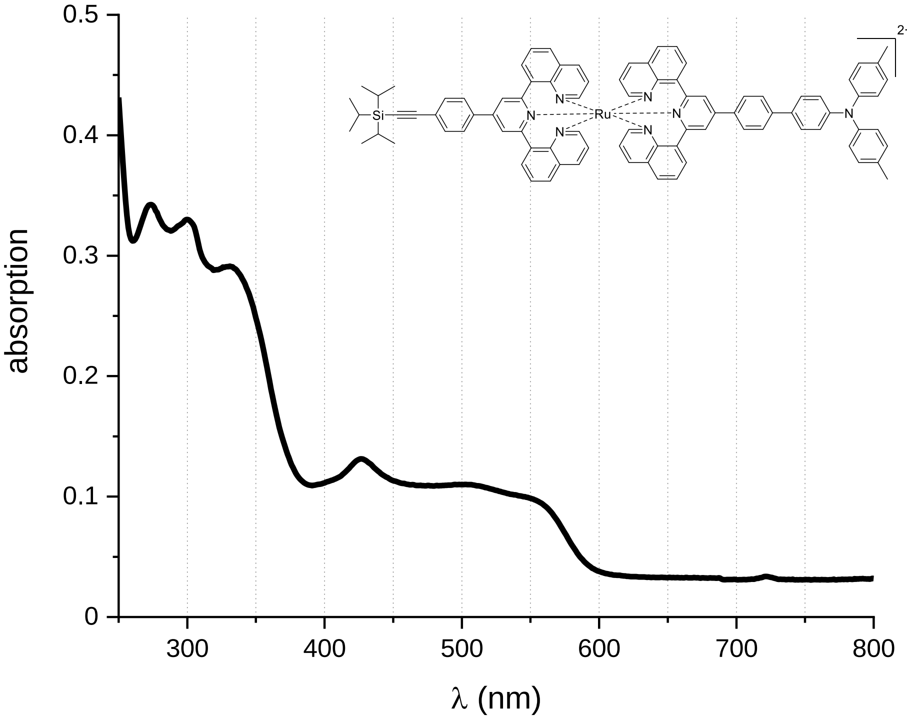

Figure S55. Absorption spectrum of [Ru(dqpPhCCTIPS)(dqpPhTARA)]$^{2+}$ in dry CH_2Cl_2 *vs*. pure CH_2Cl_2.

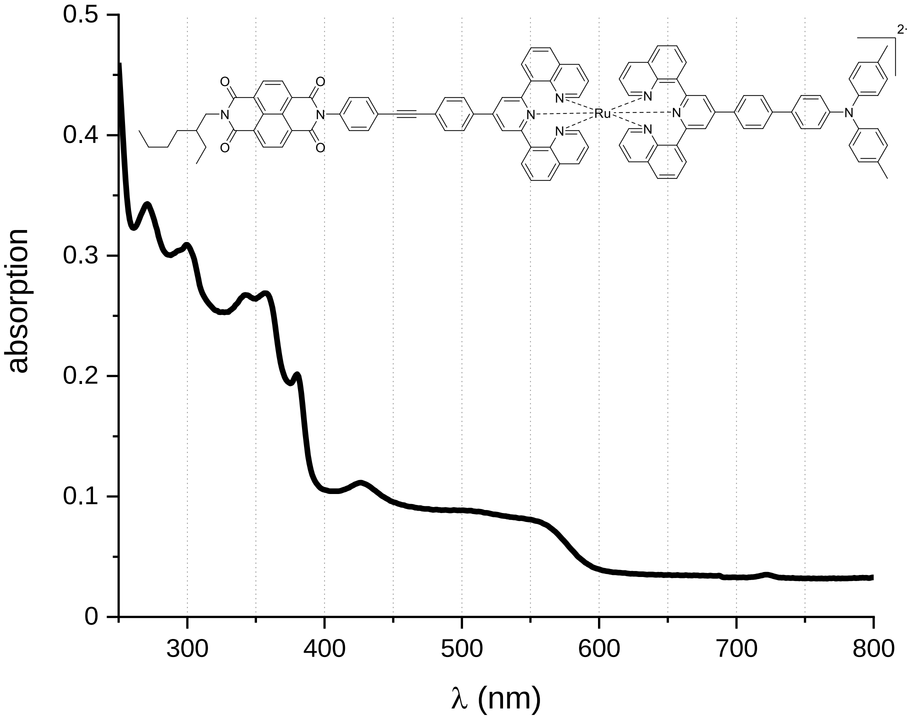

Figure S56. Absorption spectrum of [Ru(dqpPhCCPhNDI)(dqpPhTARA)]$^{2+}$ in dry CH_2Cl_2 *vs*. pure CH_2Cl_2.

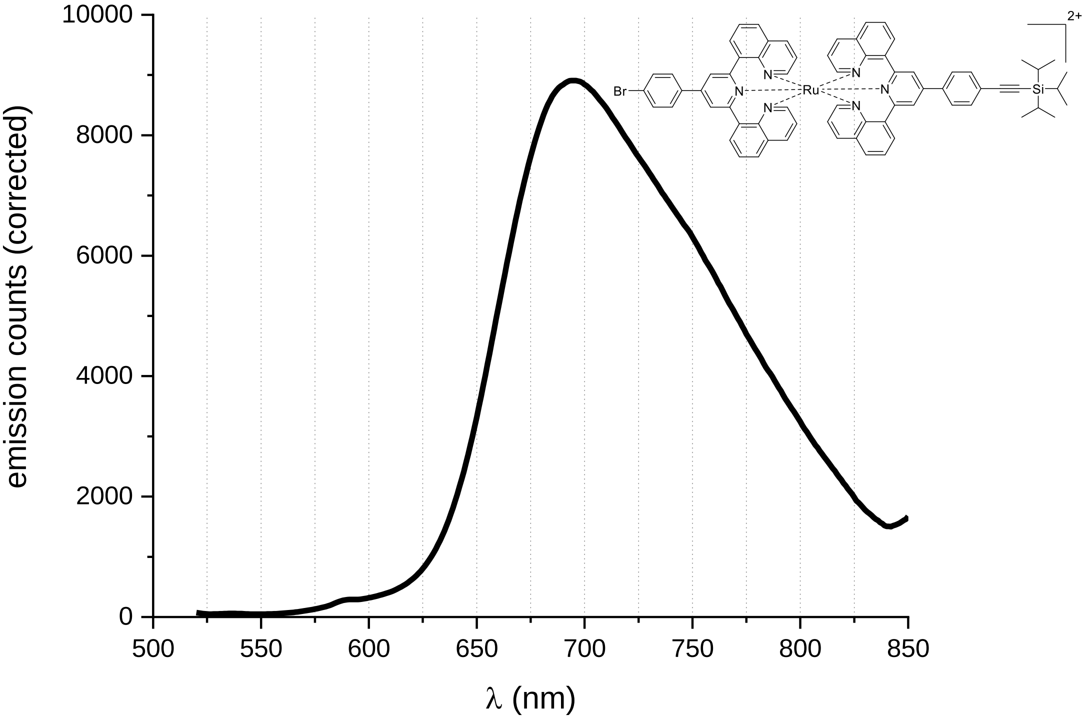

Figure S57. Emission spectrum of [Ru(dqpPhBr)(dqpPhCCTIPS)]$^{2+}$ in dry CH_2Cl_2 with excitation at 500 nm. Counts are corrected by dividing by the absorption at 500 nm.

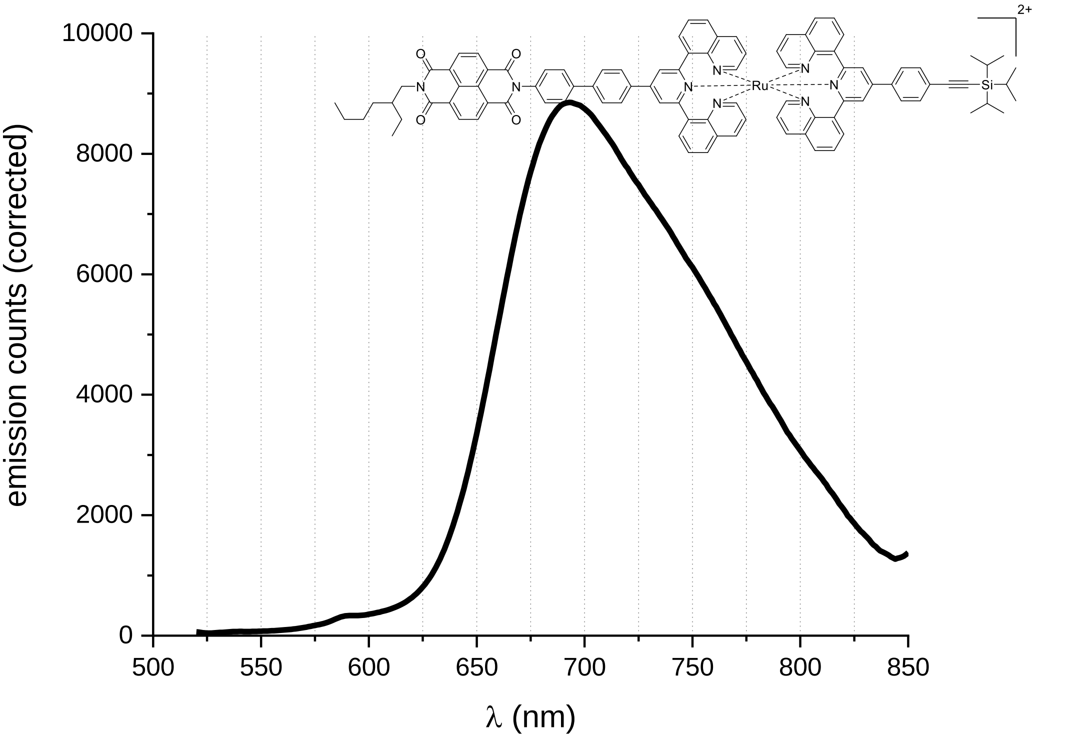

Figure S58. Emission spectrum of [Ru(dqpPhPhNDI)(dqpPhCCTIPS)]$^{2+}$ in dry CH_2Cl_2 with excitation at 500 nm. Counts are corrected by dividing by the absorption at 500 nm.

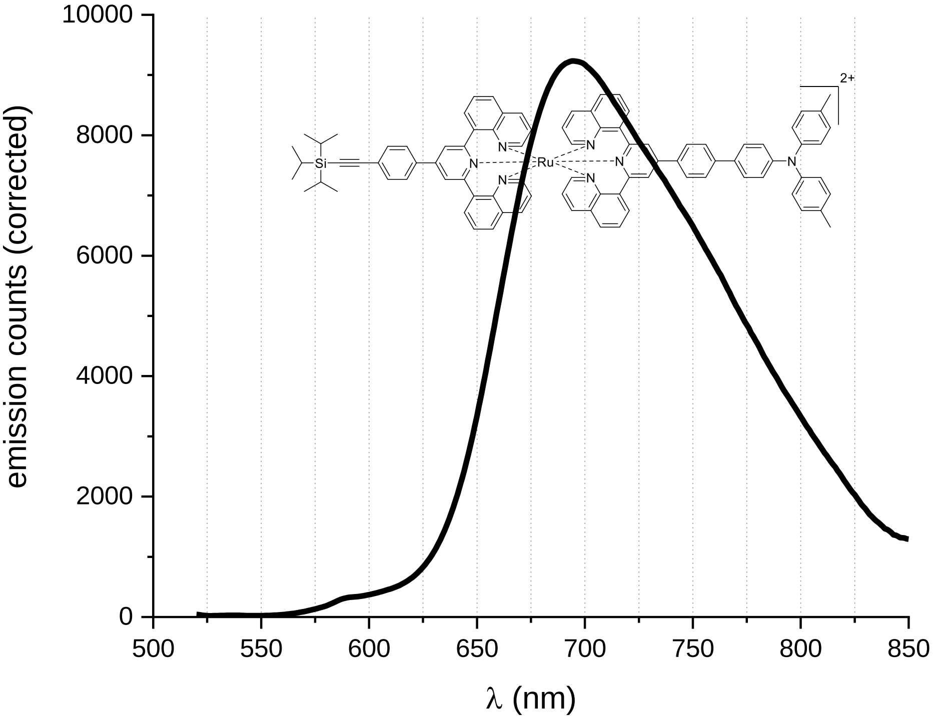

Figure S59. Emission spectrum of [Ru(dqpPhCCTIPS)(dqpPhTARA)]$^{2+}$ in dry CH_2Cl_2 with excitation at 500 nm. Counts are corrected by dividing by the absorption at 500 nm.

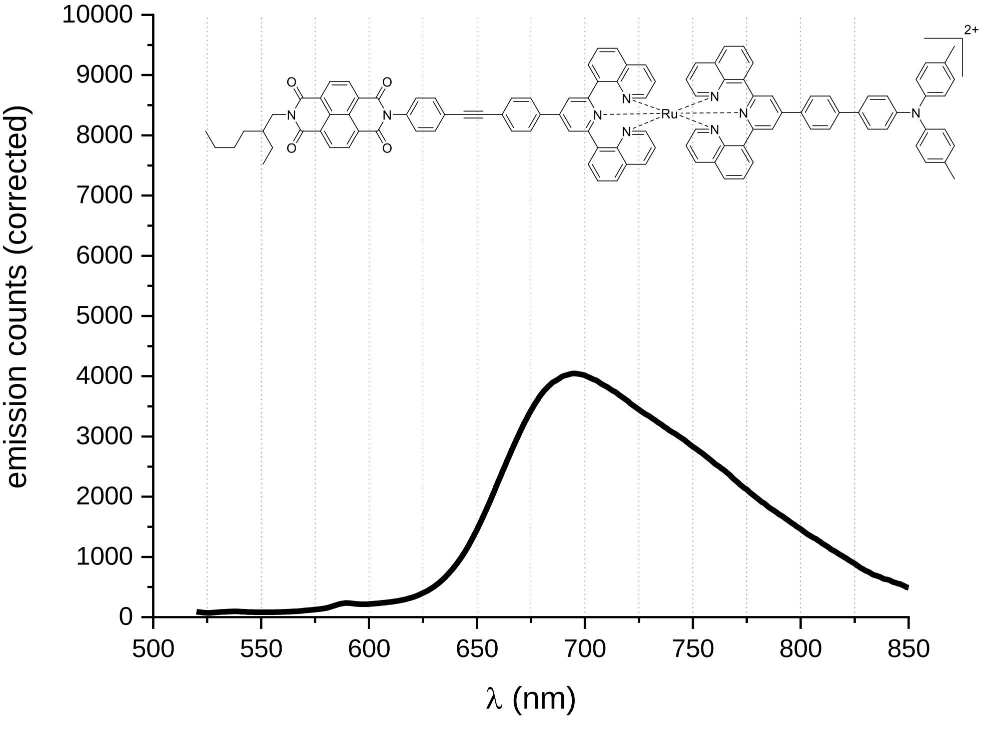

Figure S60. Emission spectrum of [Ru(dqpPhCCPhNDI)(dqpPhTARA)]$^{2+}$ in dry CH_2Cl_2 with excitation at 500 nm. Counts are corrected by dividing by the absorption at 500 nm.

References

1. M. Jaeger, L. Eriksson, J. Bergquist and O. Johansson, Synthesis and Characterization of 2,6-Di(quinolin-8-yl)pyridines. New Ligands for Bistridentate Ru_{II} Complexes with Microsecond Luminescent Lifetimes. *J. Org. Chem.*, **2007**, *72*, 10227-10230.
2. T. Schlotthauer, R. Schroot, S. Glover, L. Hammarstroem, M. Jaeger and U. S. Schubert, A multidonor–photosensitizer–multiacceptor triad for long-lived directional charge separation. *Phys. Chem. Chem. Phys.*, **2017**, *19*, 28572-28578.
3. M. P. Santoni, A. K. Pal, G. S. Hanan, A. Proust and B. Hasenknopf, Paramagnetic Ru(III) complexes of tridentate ligands: Characterization of useful intermediates for heteroleptic Ru(II) complexes. *Inorg. Chem. Commun.*, **2011**, *14*, 399-402.
4. C. Friebe, H. Goerls, M. Jaeger and U. S. Schubert, Linear Metallopolymers from Ruthenium(II)-2,6-di(quinolin-8-yl)pyridine Complexes by Electropolymerization – Formation of Redox-Stable and Emissive Films. *Eur. J. Inorg. Chem.*, **2013**, *2013*, 4191-4202.
5. J. K. Lee, S. Ko and Z. N. Bao, In Situ Hetero End-functionalized Polythiophene and Subsequent “Click” Chemistry with DNA. *Macromol. Rapid Commun.*, **2012**, *33*, 938-942.
6. N. C. Bruno, M. T. Tudge and S. L. Buchwald, Design and Preparation of New Palladium Precatalysts for C–C and C–N Cross-Coupling Reactions. *Chem. Sci.*, **2013**, *4,* 916-920.
7. K. Tambara, N. Ponnuswamy, G. Hennrich and G. D. Pantos, Microwave-assisted Synthesis of Naphthalenemonoimides and N-Desymmetrized Naphthalenediimides. *J. Org. Chem.*, **2011**, *76*, 3338-3347.
8. Z. Zhang, H. Wang, J. J. Shi, Y. P. Xu, L. Wang, S. Shihadeh, F. J. Zhao, X. Q. Hao, P. S. Wang, C. L. Liu, M. Wang and X. P. Li, Stepwise Self-assembly and Dynamic Exchange of Supramolecular Nanocages Based on Terpridine Building Blocks. *Macromol. Rapid Commun.*, **2018**, *39*, 1800404.
9. C. J. Aspley and J. A. G. Williams, Palladium-catalysed Cross-Coupling Reactions of Ruthenium Bis-Terpyridyl Complexes: Strategies for the Incorporation and Exploitation of Boronic Acid Functionality. *New J. Chem.*, **2001**, *25*, 1136-1147.
10. T. Schlotthauer, B. Suchland, H. Goerls, G. A. Parada, L. Hammarstroem, U. S. Schubert and M. Jaeger, Aryl-decorated Ru_{II} Polypyridyl-type Photosensitizer Approaching NIR Emission with Microsecond Excited State Lifetimes. *Inorg. Chem.*, **2016**, *55*, 5405-5416.
